# Harnessing Naturally Occurring Tumor Immunity: A Clinical Vaccine Trial in Prostate Cancer

**DOI:** 10.1371/journal.pone.0012367

**Published:** 2010-09-01

**Authors:** Mayu O. Frank, Julia Kaufman, Suyan Tian, Mayte Suárez-Fariñas, Salina Parveen, Nathalie E. Blachère, Michael J. Morris, Susan Slovin, Howard I. Scher, Matthew L. Albert, Robert B. Darnell

**Affiliations:** 1 Laboratory of Molecular Neuro-Oncology, Rockefeller University, New York, New York, United States of America; 2 Center for Clinical and Translational Science, The Rockefeller University, New York, New York, United States of America; 3 Howard Hughes Medical Institute and Laboratory of Molecular Neuro-Oncology, The Rockefeller University, New York, New York, United States of America; 4 Genitourinary Oncology Service, Department of Medicine, Memorial Sloan-Kettering Cancer Center, New York, New York, United States of America; 5 Department of Neuro-Oncology, Memorial Sloan-Kettering Cancer Center, New York, New York, United States of America; Karolinska Institutet, Sweden

## Abstract

**Background:**

Studies of patients with paraneoplastic neurologic disorders (PND) have revealed that apoptotic tumor serves as a potential potent trigger for the initiation of naturally occurring tumor immunity. The purpose of this study was to assess the feasibility, safety, and immunogenicity of an apoptotic tumor-autologous dendritic cell (DC) vaccine.

**Methods and Findings:**

We have modeled PND tumor immunity in a clinical trial in which apoptotic allogeneic prostate tumor cells were used to generate an apoptotic tumor-autologous dendritic cell vaccine. Twenty-four prostate cancer patients were immunized in a Phase I, randomized, single-blind, placebo-controlled study to assess the safety and immunogenicity of this vaccine. Vaccinations were safe and well tolerated. Importantly, we also found that the vaccine was immunogenic, inducing delayed type hypersensitivity (DTH) responses and CD4+ and CD8+ T cell proliferation, with no effect on FoxP3+ regulatory T cells. A statistically significant increase in T cell proliferation responses to prostate tumor cells *in vitro* (p = 0.002), decrease in prostate specific antigen (PSA) slope (p = 0.016), and a two-fold increase in PSA doubling time (p = 0.003) were identified when we compared data before and after vaccination.

**Conclusions:**

An apoptotic cancer cell vaccine modeled on naturally occurring tumor immune responses in PND patients provides a safe and immunogenic tumor vaccine. (ClinicalTrials.gov number NCT00289341).

**Trial Registration:**

ClinicalTrials.gov NCT00289341

## Introduction

Tumor immunity in patients with paraneoplastic neurologic disorders (PND) have been studied with the hope of uncovering principles that can be applied to the general population of cancer patients[1], [2]. These studies demonstrated tumor antigen-specific CD8^+^ T cells in the peripheral blood of PND patients[3], [4], but also generated a paradox. PND antigens are normally expressed in the brain, and are ectopically expressed in tumors, but are not expressed in dendritic cells (DCs) that are necessary to prime naïve T cell responses.[5] Based on observations made with lupus antigens[6], we hypothesized that apoptotic cells might serve as an effective means of antigen transfer into DCs and presentation on MHC I for the activation of CD8^+^ T cells[7]. This proved to be correct for both tumor[3] and viral antigens[8], and it is likely that phagocytosis of apoptotic cells serve as a general means by which the immune system surveys antigens throughout life. Insights from studying natural tumor immunity in PND provide a compelling base upon which to model clinical studies[2], [9], [10].

Several observations support the suggestion that apoptotic tumor cells may serve as a potent source of antigen for stimulating host immune responses *in vivo*. Theoretically, all potential tumor antigens within an apoptotic tumor cell, and multiple epitopes from each antigen, can be cross-presented by DCs, where processed antigen may be placed on all (typically six) MHC I alleles. This offers considerable advantages over peptide-pulsed DC protocols, in which the investigator must have knowledge of the tumor antigen and must choose specific MHC I-restricted peptides for antigen stimulation. Antigen presentation from apoptotic cells has been estimated to be 10,000–50,000 more efficient than free peptide in loading the MHC molecules of a DC[11], [12]. Apoptotic material is processed by a natural pathway: apoptotic cells are internalized by DC-restricted receptors, including the α_v_β_5_ integrin receptor[13], then processed within DCs by distinct pathways[14]. These DCs then generate both MHC I and MHC II peptide epitopes[13], leading to the activation of both CD8^+^ and CD4^+^ T cells. Since the ability of DCs cross-presenting apoptotic cells to activate effector CD8^+^ T cells requires signals from CD4 helper cells[15], the ability of apoptotic material to be loaded on both MHC I and MHC II molecules is of particular importance in considering its potential in immunotherapy.

DCs presenting apoptotic tumor cells stimulate T cell responses in animals and *in vitro* [16]. Clinically, several studies have used killed tumor cells in vaccine trials, including glial[17]–[21], prostate[22], melanoma[23], breast[24], ovarian[25] and pediatric solid tumor cells[26] (reviewed in [9], [16], [27]). These studies have not focused on apoptotic death per se, but rather have killed tumor cells by various means (e.g. freeze-thawing, or large amounts of UVB and gamma irradiation), leading to incompletely characterized mixtures of necrotic and apoptotic cell death. Interpretation of these studies is complicated by controversy regarding the immunogenic potency of different forms of dead cells[28]. Nonetheless, some trials have indicated the potential for immunologic and clinical responses to autologous DC presenting dead tumor cells[19], [23]. Here we set out to more precisely test the relationship between induction of PND-like tumor immune responses and development of a clinical vaccine. We induced apoptotic death of LNCaP prostate cancer cells, and allowed them to be phagocytosed by autologous DCs generated from prostate cancer patients' peripheral blood monocytes; such DCs were previously shown to stimulate both CD8^+^ and CD4^+^ T cells *in vitro* [29], and in a B16 mouse melanoma model DCs cross-presenting apoptotic tumor were effective in preventing tumor growth (Blachere et al., unpublished data). Here we report the safety and immunogenicity of DC/apoptotic LNCaP prostate tumor cells in a controlled study of 24 prostate cancer patients.

## Results

### Study Population

**Figure 1:**
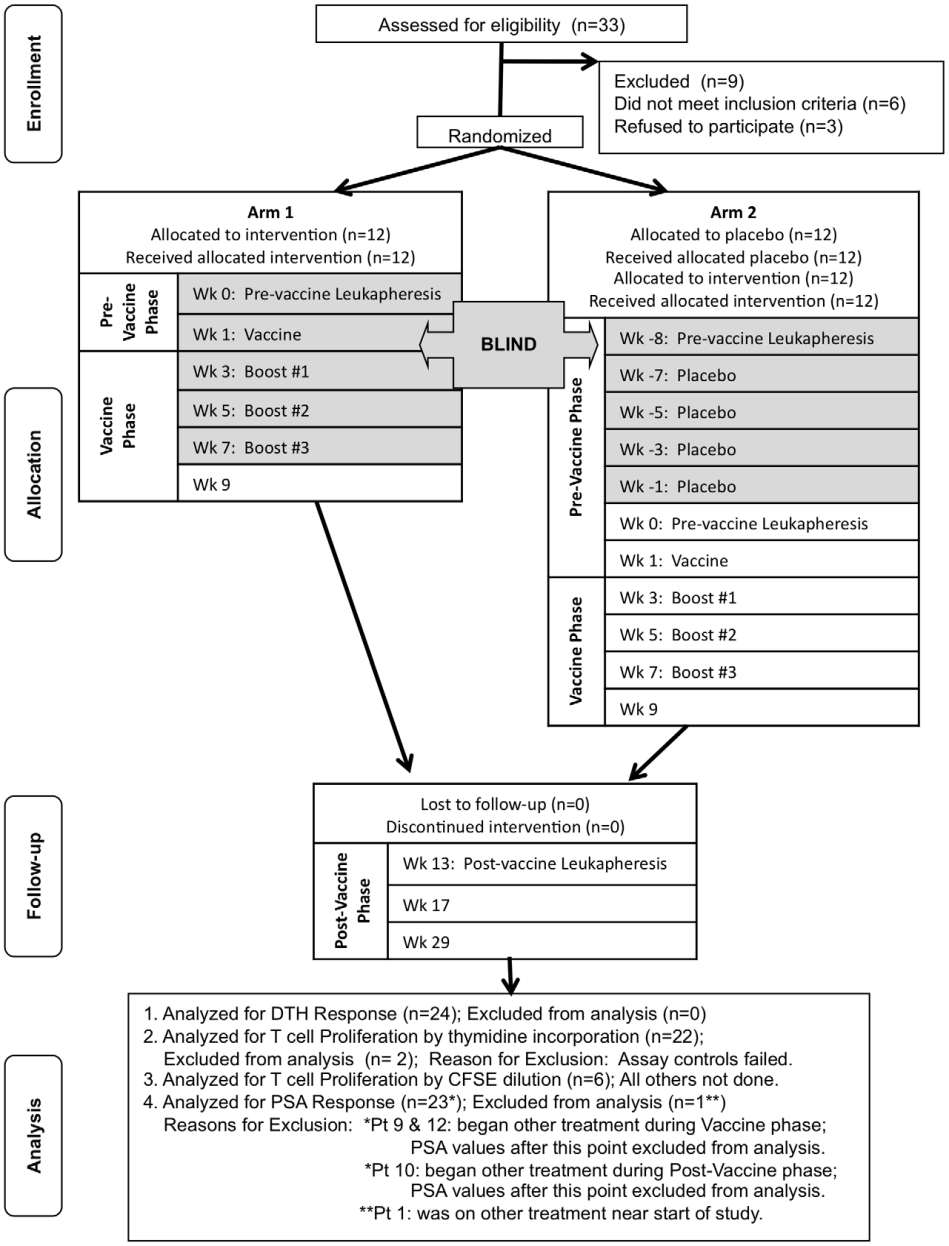
Study Design (CONSORT Diagram). Patients were screened and randomized into 1 of 2 arms, each with 12 patients. Patients in both arms were blind during the vaccine/placebo phases until Week 8. Patients in Arm 1 continued into the post-vaccine phase while patients in Arm 2 crossed over into the vaccine phase before entering post-vaccine phase.

Twenty-four consecutive eligible patients were vaccinated with DC/LNCaP, together with control vaccinations. A total of 28.4–78.9 million (average 50.3 million) DCs cross-presenting apoptotic LNCaP tumor cells were given per patient, divided over 4 doses, each 2 weeks apart (Figure 1). At the time of study entry, 11 of 12 patients in Arm 1 and 10 of 12 patients in Arm 2 had only biochemical relapse with no other evidence of metastatic disease (Table 1). The mean age of patients was 62.5±6.7 and 65.7±9.2 years and the mean Gleason score at study entry was 7.25 and 7.17 in Arms 1 and 2, respectively.

**Table 1:** Patient characteristics.

Pt	Age	Previous Treatments	Clinical Status	Gleason Score	PSA at Study Entry
1	55	Leuprolide Acetate, Bicalutamide, Nilutamide	CM/HR	8	0.69
2	80	RRP, Leuprolide Acetate, Bicalutamide	BCR/HR	7	2.71
3	65	RRP, Salvage RT	BCR	7	0.79
4	58	RRP	BCR	6	0.49
5	64	RRP	BCR	7	0.59
6	75	RRP	CM	9	0.57
7	81	RT	BCR	6	8.66
8	54	RRP, Salvage RT	BCR	6	0.46
9	60	RRP, IL-2 and J-591 Ab Study, Testosterone Gel/ Leuprolide or Goserelin Acetate/Docetaxel Study	BCR	7	7.56
10	57	Brachytherapy, Salvage RRP	BCR	7	6.38
11	56	RRP, Salvage RT, Leuprolide Acetate, Bicalutamide	BCR/HR	8	5.24
12	69	RRP, Salvage RT, Goserelin Acetate	BCR/HR	7	0.86
13	63	RRP, Salvage, RT, Testosterone Gel/Leuprolide or Goserelin Acetate/Docetaxel Study	BCR/HR	8	0.48
14	53	RRP, Salvage RT, Leuprolide Acetate	LR/HR	8	0.94
15	57	RRP, Salvage RT, Leuprolide Acetate, Bicalutamide	BCR	9	0.29
16	64	RRP	BCR	7	15.27
17	68	RT, Testosterone Gel/Leuprolide or Goserelin Acetate/Docetaxel Study	BCR	8	4.61
18	74	RRP	BCR	6	40.25
19	67	RRP, Salvage RT	BCR	7	5.02
20	63	RRP, Leuprolide Acetate, Bicalutamide	BCR	7	2.15
21	59	RRP, Salvage RT	BCR	7	0.62
22	70	RRP, Salvage RT	BCR	7	1.75
23	55	RRP, Salvage RT	BCR	7	0.44
24	68	RRP, Salvage RT	BCR	7	1.27

Previous Treatments: RRP = Radical Retropubic Prostatectomy, RT = Radiation Therapy. Clinical Status: CM = Clinical Metastasis, HR = Hormone Refractory, BCR = Biochemical Relapse, LR = Local Recurrence.

### DC vaccine characteristics

**Figure 2:**
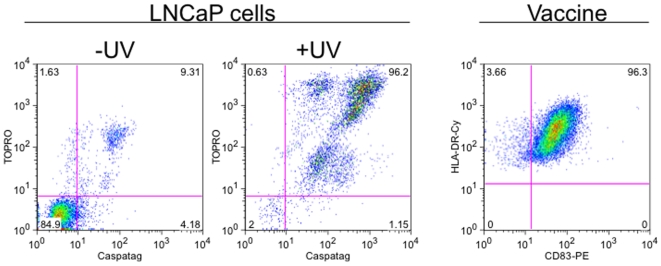
Preparation of Vaccine. UV irradiation (+UV) specifically induced apoptosis in LNCaP cells as indicated by 96% Caspatag+ TOPRO+ staining 38 hours post UV. DC cocultured with apoptotic LNCaP cells (Vaccine) are mature, with >96% CD83 positive cells. Data shown is representative of all 24 vaccines prepared.

DCs were cocultured with LNCaP or LNCaP-M1 cells that were >90% apoptotic (Figure 2). All DC vaccine preparations administered met criteria for viability and maturity (Table 2 and Figure 2). DC function was monitored by allo-MLR; DCs stimulated 2×10^5^ allogeneic T cells to incorporate ≥10^5^ CPM of ^3^H-thymidine on day 5 after an 18-hour ^3^H thymidine pulse (data not shown).

**Table 2:** Dendritic cell characteristics.

Vaccine Groups	% CD14 + Median (Interquartile Range)	% CD83 + Median (Interquartile Range)	% PI + Median (Interquartile Range)
DC/LNCaP	0.13 (0.83)	86.94 (8.86)	4.14 (5.87)
DC/LNCaP-M1	0.09 (0.48)	87.82 (6.86)	4.37 (4.69)
DC/KLH	0.09 (0.41)	96.19 (3.59)	1.25 (1.72)
DCs alone	0.17 (0.45)	96.41 (4.94)	1.52 (1.94)

Vaccine groups were prepared from patient monocytes as described. DCs were assessed for maturation by staining for expression of surface markers, CD14 and CD83, and for viability using PI stain. Cells were assessed by flow cytometry on Day 8 prior to vaccine release. All vaccine groups administered met release criteria.

### Safety

The incidence of injection-site and systemic reactions to vaccine are presented in Table 3. No vaccine related serious adverse events were observed. Only 1 toxicity was significantly different between the placebo and vaccine groups in the single blind phase of the study: grade 1 or 2 injections site reactions that occurred in 11 of 12 patients in the vaccine group (p<0.001), attributable to vaccine (but not placebo) generating DTH-like responses. There was no symptomatic evidence of autoimmune disease in any patient, including vasculitis, thyroiditis, colitis, neurologic disease, endocrinopathy or cardiomyopathy.

**Table 3:** Adverse events.

	Single Blind		Unblinded	
	Arm 2 Placebo Phase	Arm 1 Vaccine Phase	Arm 2 Vaccine Phase	Arm 1 and 2 Post-Vaccine Phase
**Total number of Adverse Events in each phase**	80	120	90	76
**No. Pts with at least 1 AE** *No. of pts (Total No. of pts)*	12 (12)	12 (12)	12 (12)	22 (24)
**RELATED TO VACCINE:**				
**Adverse Events** *No. of events (No. of pts)*				
injection site reaction	2 (2)	22 (11)	29 (12)	0
injection site reaction (grade 2)	0	4 (2)	0	0
**Serious Adverse Events** *No. of events (No. of pts)*	0	0	0	0
**NOT RELATED TO VACCINE:**				
**Frequently Occurring Adverse Events (occurring ≥5 times in study)** *No. of events (No. of pts)*				
albumin, serum, low	0	1 (1)	4 (3)	0
albumin, urine, high	2 (1)	3 (3)	0	3 (3)
ALT, serum, high	3 (3)	4 (2)	0	2 (2)
ALT, serum, high (grade 2)	0	0	1 (1)	0
ANA, high	1 (1)	1 (1)	3 (3)	4 (4)
BUN, serum, high	1 (1)	4 (3)	2 (2)	1 (1)
chloride, serum, high	4 (4)	1 (1)	0	2 (2)
CO2, serum, low	4 (4)	3 (2)	2 (2)	1 (1)
creatinine, serum, high	1 (1)	3 (2)	2 (2)	2 (2)
diarrhea/loose stools	1 (1)	5 (3)	0	0
edema, lower extremities	2 (2)	0	3 (1)	1 (1)
eosinophils, high	3 (3)	1 (1)	1 (1)	1 (1)
fatigue	5 (5)	6 (5)	5 (4)	0
glucose, serum, high, non-fasting	5 (5)	6 (6)	2 (1)	7 (6)
ketones, urine, high	2 (2)	1 (1)	1 (1)	3 (3)
potassium, serum, high	3 (3)	0	6 (5)	1 (1)
potassium, serum, high (grade 2)	1 (1)	0	0	0
rash	2 (2)	2 (1)	0	3 (2)
URI	3 (3)	3 (3)	1 (1)	0
**Serious Adverse Events** *No. of Events (No. of pts)*	0	1 (1)	0	3 (1)
Hospitalization: Cardioversion for atrial fibrillation	0	1 (1)	0	0
Hospitalization: Urinary retention	0	0	0	1 (1)
Elective Hospitalization: Cholecystectomy	0	0	0	1 (1)
Elective Hospitalization: Inguinal hernia repair	0	0	0	1 (1)

Study visits during the placebo and vaccine phases are identical. Placebo injections consisted of vaccine vehicle only. All adverse events are Grade 1 unless otherwise specified. There were no vaccine related serious adverse events. The only statistically significant adverse event was the number of patients having injection site reactions between Arm 1 vaccine phase and Arm 2 pre-vaccine placebo phase (p = 0.001, Fisher's exact test). All other adverse events listed are not statistically significant (p≥0.2 in all cases, Fisher's exact test) between Arm 1 vaccine phase and Arm 2 pre-vaccine placebo phase.

### Immune response

**Figure 3:**
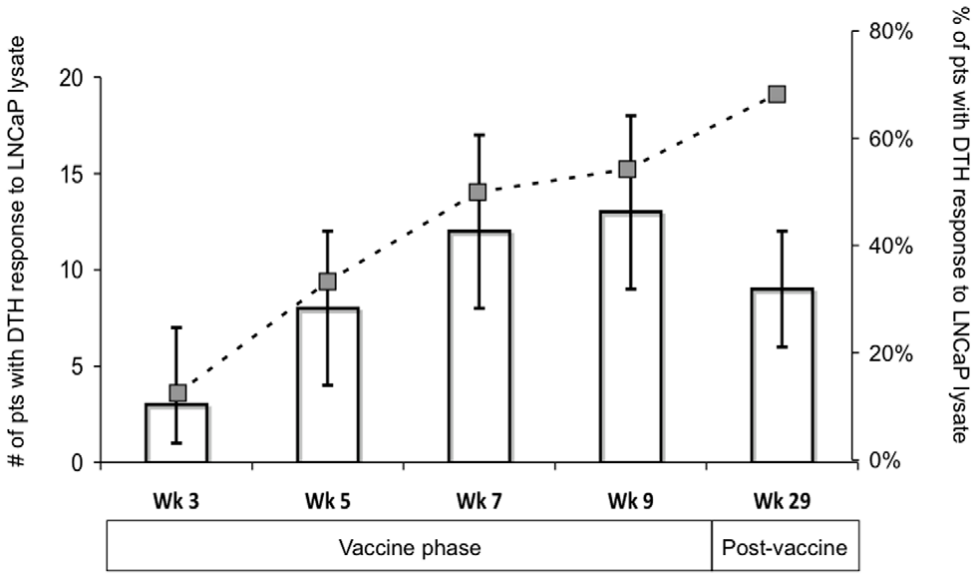
DTH Responses. Vaccine induced DTH response to LNCaP cell lysates injected intradermally were measured at the indicated times. Week 1 was baseline, at which time no patients had DTH responses (data not shown). DTH responses were considered positive at ≥5 mm erythema read at 48 hours after placement. Bars indicate the number of patients with positive responses at each time point. Error bars represent 95% confidence intervals. Dotted line represents trend of percentage of patients with positive responses at each time point. Statistically significant positive DTH responses to LNCaP cell lysate appeared at Week 3 (first time point after baseline) and responses were still present in 9 of 13 patients (69%) at 22 weeks after the last booster dose (Week 29).

All patients who received the DC/keyhole limpet hemocyanin (KLH) vaccine had a positive DTH response to KLH. None of the 24 patients had a response to LNCaP lysate at baseline (week 0). All 24 patients were given LNCaP lysate as part of DTH panels at weeks 3, 5, 7, and 9. Sixteen of 24 patients (67%) had a DTH response to LNCaP lysate in at least 1 of these 4 time points, with the highest proportion of patients (54.2%) responding to LNCaP lysate at 2 weeks after the last booster (week 9). Responses were maintained in 9 of 13 patients (69%) at 22 weeks after the last booster (week 29, Figure 3). The responses to LNCaP lysate were statistically significant at all time points with a 95% confidence interval. Normal saline, given as a control, was negative at all time points in all patients.

**Figure 4:**
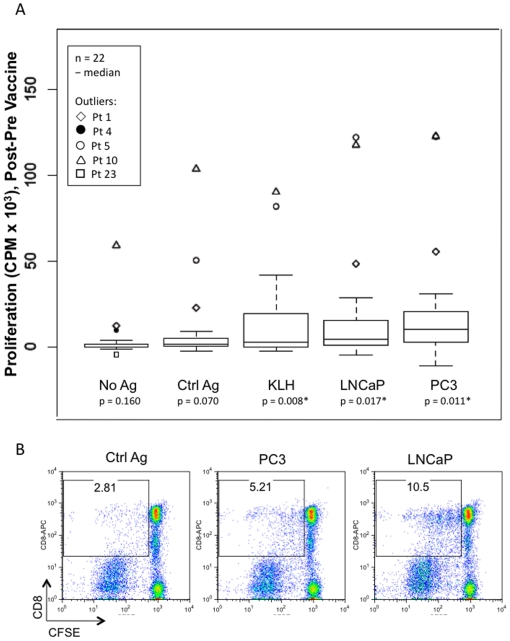
T cell proliferation response in vitro. Comparison of pre- and post-vaccine bulk T cell responses to prostate antigen. a. Apoptotic tumor cells (LNCaP and PC3, or an irrelevant cell line (3T3)) or KLH protein were co-cultured with patient peripheral blood monocytes and syngeneic bulk T cells obtained from patients pre- or post-vaccination. Monocytes without exogenous antigen (No Ag) or apoptotic 3T3 cells (Ctrl Ag), served as negative controls. Proliferation was assessed on day 5 after an 18-hour ^3^H thymidine pulse. Data is presented for 22 of 24 patients. The difference in proliferation (post- minus pre-vaccine) for each antigen group is shown in box plots. Values reported are average counts per minute (CPM) of triplicate wells. The median difference for each antigen group is shown by the line in the box. Each patient who is an outlier is indicated by a unique symbol. Statistically significant differences in pre-vaccine vs. post-vaccine T cell proliferative responses were found for KLH (p = 0.008), LNCaP (p = 0.017) and PC3 (p = 0.011). b. Bulk T cells obtained post-vaccination were stained with CFSE and cultured with DCs cross-presenting prostate antigens, LNCaP and PC3, or an irrelevant cell line (293 cells, Ctrl Ag). Cell proliferation on day 5, assessed by CFSE dye dilution, is shown on the x-axis and CD8 expression is shown on the y-axis. Percentages shown represent CD8+ cells that have divided within the bulk T cell population. Four of five additional patients tested showed similar CD8+ responses; data shown are for patient #15.

A ^3^H thymidine proliferation assay was used to assess the reactivity of T cells to KLH protein and to prostate tumor cells (either those used in vaccination (LNCaP) or to another prostate tumor cell line (PC3)). Negative control antigens included autologous monocytes and an irrelevant cell line (3T3). 3T3 infected with influenza was used as positive control. T cells were collected at week 0 (pre-vaccine) leukapheresis and week 13 (post-vaccine) leukapheresis (Figure 1). To be valid, each individual proliferation assay must have had a detectable influenza response pre- and post-vaccine. Two of 24 patients had no detectable influenza response and thus were excluded from analysis. The 22 evaluable patients, considered as a group, had no statistically significant difference in T cell response to influenza, pre- versus post-vaccine (p = 0.310, data not shown). There was a statistically significant T cell response to KLH post-vaccine vs pre-vaccine (p = 0.008), as well as to apoptotic LNCaP (p = 0.017) and apoptotic PC3 tumor cells (p = 0.011) (Figure 4a). There were no statistically significant differences in pre- vs post-vaccine T cell responses to antigen presenting cells (APCs) alone (p = 0.160), to control antigens, 3T3 (p = 0.070) or autologous monocytes (p = 0.156) (Table 4, Figure 4a and data not shown).

**Table 4:** Confidence intervals for T cell proliferation assay and Foxp3+ cell analysis.

Group:	p-value	95% CI
No Ag	0.160	(−1703.275, 9649.413)
KLH	0.008*	(4681.672, 27308.539)
LNCaP	0.017*	(3759.725, 34618.214)
PC3	0.011*	(5835.155, 38744.036)
3T3	0.070	(−862.319, 20294.713)
Foxp3+ cells	0.160	(−0.978, 0.893)

95% confidence intervals and p-values for T cell proliferation response (^3^H thymidine incorporation assay) and %Foxp3+ cells (FACS analysis) comparing cells collected pre-vaccine and post-vaccine using the paired t-test.

Based on the DTH data (Figure 3) and the ^3^H thymidine proliferation data (Figure 4a), we calculated and ranked a DTH index and a proliferation index to determine if there was an association between the two. A positive correlation was found (0.55 using the Spearman rank correlation test (p = 0.008)), indicating that patients who had positive DTH responses to LNCaP lysate also tended to have T cell proliferation responses to apoptotic LNCaP cells *in vitro*.

The proliferation response was also assessed by CFSE dye dilution assay[30]. In 5 of 6 patients, a CD8+ T cell response specific for prostate antigens was observed (Figure 4b and data not shown). We confirmed that all six patients tested also had a CD4+ T cell response to prostate antigens.

**Figure 5:**
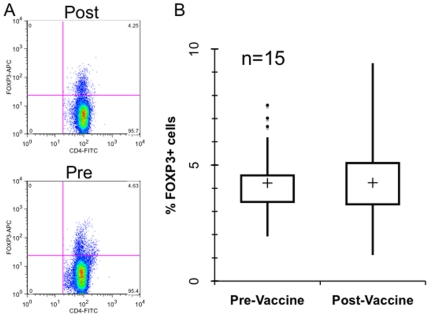
Foxp3 expression pre- and post-vaccination. a. FACS profile of Foxp3 expression in pre- and post-vaccinated peripheral blood gated on CD4+ T cells. A representative patient (#13) is shown. b. Box plots of the percent Foxp3+ cells (gated on CD4+ T cells) pre and post-vaccination in 15 representative patients, including those across the whole range of proliferative responses and changes in PSA slope. The median is shown by the +. Outliers are indicated by •. No difference in pre-vaccine vs. post-vaccine T cell expression of Foxp3 (p = 0.924) was observed.

We considered the possibility that the increased T cell proliferation response post vaccination could relate to a decline in regulatory T cells in circulation. To determine the percent of CD4+ T cells that are T regulatory cells in pre- and post-vaccinated blood samples, PBMCs were stained and assessed for Foxp3 expression (Figure 5). No difference (p = 0.924) was found in Foxp3 expression comparing pre- and post-vaccination CD4+ T cells from 15 patients (Table 4).

### PSA response

The prostate specific antigen doubling time (PSADT) was calculated for each patient during each phase of study. The median doubling time during the pre-vaccine phase was 4.5 months, and this increased to 5.4 and 8.9 months during the vaccine and post-vaccine phases respectively. There were statistically significant differences in PSADT between the pre- and post-vaccine phases (p = 0.003) and between the vaccine and post-vaccine phases (p<0.001) but not between the pre-vaccine and vaccine phases (p = 0.915).

**Figure 6:**
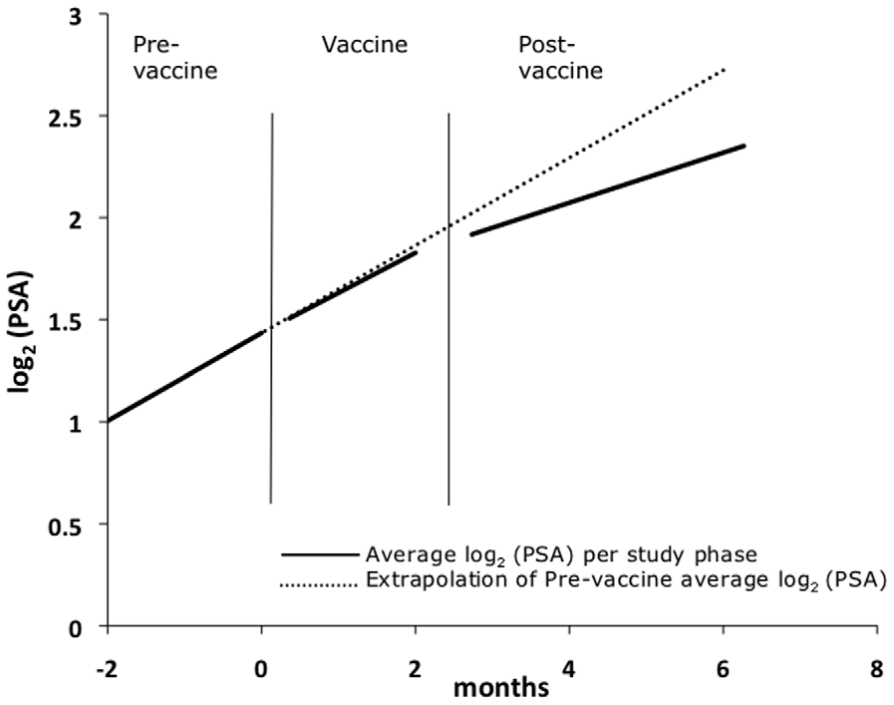
Change in PSA slope pre- to post-vaccine. Graph of the average log_2_ (PSA) slope per study phase (solid line); for comparison, an extrapolation of the pre-vaccine average log_2_ (PSA) slope is shown (dotted line). Based on the linear spline model, the average change in PSA slope of 23 patients from pre- to post-vaccine phases is −0.093/month (p = 0.016). One patient's PSA values were not included in the analysis as his pre-vaccine values were affected by other treatment near the start of study participation. Three other patients started other treatment either during or after vaccination; PSA values obtained after this point were not included in the analysis.

We also compared the slope of the PSA rise between the 3 study phases. Eighteen of 23 (78%) evaluable patients had a decrease in PSA slope between the pre- and post-vaccine phases. When considering all 23 patients, there was a statistically significant decrease in the PSA slope between the pre-vaccine and post-vaccine phases of −0.093/month (p = 0.016, Figure 6 and Table 5). There was no statistical difference in PSA slope between the pre-vaccine and vaccine phases (−0.018/month, p = 0.681) or the vaccine and post-vaccine phases (−0.075/month, p = 0.098). We evaluated whether this PSA slope change might result from the development of serum anti-PSA antibodies. Pre- and post-vaccine serum was assessed by measuring serum antibody reactivity to purified PSA protein on Western blot, using PSA monoclonal antibodies as a positive control. We found no evidence for antibody reactivity to PSA (data not shown). Nonetheless, we cannot rule out that antibodies are not detectable due to antigen/antibody complexes being formed and that these somehow aided in clearing PSA from the serum.

**Table 5:** Confidence intervals for PSA slopes.

PSA Slopes by Study Phases:	p-value	95% CI
Pre- vs Post	0.016	(−0.1694, −0.0166)
Pre- vs Vaccine	0.681	(−0.1044, 0.0682)
Vaccine vs Post	0.098	(−0.1613, 0.0134)

95% confidence intervals and p-values for PSA slopes comparing pre- vs vaccine, vaccine vs post-vaccine, and pre- vs post-vaccine phases from the linear mixed model.

We then stratified the study population with two more fixed effects on a mixed model and conducted a likelihood ratio test; the patients that had a DTH response to LNCaP lysate had a significantly different PSA slope change when compared to those that had no DTH response to LNCAP lysate (p = 0.004). Sixteen of 24 patients had DTH responses to LNCaP in at least one time point. This group of patients had a statistically significant change in PSA slope between the pre-vaccine and post-vaccine phases (−0.105/month, p = 0.020, Table 6). Eight of 24 patients had no response to LNCaP lysate at any time point, and these patients had no statistically significant change in slope between the pre-and post-vaccine phases (−0.033/month, p = 0.631). When we stratified the study population into 2 groups based on PSA value at study entry, the likelihood ratio test indicated a significantly different PSA slope change between these two groups (p<0.001). With the mixed model, we found that those who had a PSA ≥1 ng/ml at study entry (15 patients) had a significant PSA slope change between pre- to post-vaccine phase (−0.099/month, p = 0.031, Table 6) while the patient subgroup that had a PSA <1 ng/ml at study entry (8 patients) did not (−0.078/month, p = 0.279). Taken together, these data demonstrate that the study patients taken as a group had a significant reduction in the rate of rise of PSA after vaccination, and that this effect was particularly evident in those with immunologic response and more readily measurable disease.

**Table 6:** Confidence intervals for PSA slopes upon stratification.

Stratified by:		p-value	95% CI
Whole group		0.016	(−0.1694, −0.0166)
Response by DTH to LNCaP lysate	Non-Responders	0.631	(−0.1682, 0.1022)
	Responders	0.020	(−0.1932, −0.0168)
PSA at start of study	<1 ng/ml	0.279	(0.2191, 0.0631)
	≥1 ng/ml	0.031	(−0.1872, −0.0108)

95% confidence intervals and p-values for PSA slopes comparing pre- vs vaccine, upon stratification by DTH response or no response to LNCaP lysate or upon stratification by PSA <1 ng/ml or 1 ng/ml at start of study.

## Discussion

Patients with PND develop effective tumor suppression of common cancer types[2] that are likely to be triggered by immune recognition of ectopic expression of neuronal proteins by those cancers. To trigger such immune responses, we hypothesized and demonstrated that apoptotic tumor serves as a potent source of antigen for presentation by APCs[7], [29]. The focus of this study was to evaluate the safety and immunogenicity of mimicking this means of triggering tumor immunity by using prostate cancer patients' DCs cross-presenting apoptotic tumor cells as a cancer vaccine.

Despite the conceptual link between the development of apoptotic cells as a vaccine and tumor immunity in PND, we found no evidence that this approach triggered autoimmune disease in our patients. We did note small ANA elevations post vaccine in 5 patients (titer of 1∶160 in one patient, ≤1∶80 in four patients) which resolved over time in 4/5 patients. However, we also noted that of 7 patients with detectable ANA levels pre-vaccination, 5 became lower after vaccination. Statistical analysis of ANA changes pre versus post vaccine/placebo in Arm 1 versus Arm 2 revealed no significant differences (Fisher's exact test), and we conclude that ANA changes were not clinically meaningful; moreover, such transient responses have commonly been seen in DC based vaccines[26], [31], [32]. One reason that we chose prostate cancer as an initial tumor for study using this vaccine approach is that these tumors are rarely associated with PND. Despite the safety of the vaccine here, caution is warranted in extending this approach to patients harboring tumors known to be associated with PND (for example gynecologic tumors expressing the cdr2 or Nova antigens and small cell lung cancers expressing the Hu antigen)[1], [2].

Our dendritic cell/apoptotic tumor vaccine was immunogenic. Sixty-seven percent of patients developed DTH responses to LNCaP antigens. Furthermore, these DTH responses were positively correlated with post-vaccine bulk T cell proliferation responses. This high level of immunogenicity was similar to that reported in other studies of peptide-pulsed or tumor cell associated DC vaccines. Importantly, these responses included CD8+ T cell responses to prostate tumor cells (Figure 4b). This is significant, as a critical determinant of successful tumor vaccines is likely to be induction of CD8+ T cell responses[33]. The ability to detect such responses here is consistent with the observation that cross-presentation of apoptotic cells are able to stimulate naïve and memory CD8+ T cell responses to tumor cells[3] or to virally infected cells[8] *ex vivo*. Due to the nature of the disease, autologous tumor cells were not available for testing T cell responses. However, both the CD4 and CD8 proliferation responses were detected to prostate tumor cell lines, despite the high background responses seen in T cells post vaccination (Figure 4a). Such background responses have been seen before in DC-based vaccines and are of uncertain etiology[34], and may be reflected in transient increases in ANA seen in some patients. It is likely that patients had variable immune responses to tumor vaccination, either resulting from intrinsic differences in immune repertoire, or from actions of the tumor itself to modify patient immune responses[35].

Significantly, we found that PSA slopes decreased and PSADT increased after vaccination in our patient population as a whole (p = 0.016). We hypothesized that if this correlation was related to the immunogenicity of the vaccine, PSA changes should be present in the subset of patients showing immunogenic response to vaccine but not in those who do not. In fact, patients who had DTH responses to LNCaP after vaccination had significant decreases in PSA slope (p = 0.020), compared to patients who did not have DTH responses (p = 0.631). Taken together, our data suggests that the changes seen in PSA slope represent an immune response to patient tumor cells *in vivo*.

Although variable immunologic and clinical responses have been reported to vaccines using dead tumor cells as a source of antigen, these studies have not focused on using pure, well-defined populations of apoptotic tumor cells. We used UV-B irradiation to induce apoptotic (not necrotic) death in >90% of the prostate cell line used for the vaccine; nonetheless, our side-effect profile was very low, similar to other tumor vaccines. Most other studies have used gamma irradiation or freeze-thawing, generating variable mixtures of apoptotic and necrotic cells, which may underlie differences in immunogenic potential[28].

Taken together, the results presented in this study provide initial safety and immunogenicity data for a vaccine mimicking what we believe is a critical trigger for naturally occurring effective tumor immune responses seen in PND patients. These responses correlate with a clinically relevant response to patient tumor, as assessed by highly statistically significant effects on PSA slope and doubling time. These observations suggest that this vaccination approach warrants further exploration as a safe and potent means of triggering tumor immune response in the general population of cancer patients. Future vaccine modifications to be considered are the addition of immune adjuvants during vaccine preparation ex vivo or in conjunction with vaccine administration in vivo[9], [36], or the use of autologous tumor as a source of apoptotic antigen. In addition, a safe means of vaccinating against prostate (or other) cancers may serve in a synergistic manner with other immune-stimulating agents, such as CTLA4-Ig, which are showing promise in combined immunotherapies in prostate[37] and other cancers[38].

## Methods

The protocol for this trial and supporting CONSORT checklist are available as supporting information; see Checklist S1 and Protocol S1.

### Patients and Study Design

#### Ethics Statement

The study was approved by The Rockefeller University Institutional Review Board (RDA-0466) and the FDA (IND 10710). Written consent was obtained from all patients. No *de novo* cell lines were generated in this study.

#### Study Design

The study was conducted at the Rockefeller University in New York; twenty-four patients aged 53 to 81 were enrolled between November 2003 and February 2006. All authors vouch for the completeness and accuracy of the data and its analysis and participated in writing the article.

Twenty-four patients were randomly assigned to one of two arms for the purpose of assessing vaccine safety, our primary endpoint (Figure 1). All patients were blinded. Twelve patients assigned to Arm 1 received vaccine followed by 3 vaccine boosts at 2-week intervals, for a total of four injections over eight weeks, and were unblinded after the last booster. Twelve patients assigned to Arm 2 received placebo (vehicle (5% DMSO in saline)) for each of four injections, were unblinded, crossed over to the vaccine phase, and received vaccine followed by 3 boosts at 2-week intervals. After the last booster, patients in both groups were followed for up to 22 weeks. All time points in both arms up to and including the day of the first vaccination with DC vaccine were considered pre-vaccine phase. All time points from the first booster through the first follow up visit after the final vaccination (week 9) was considered vaccine phase. All remaining time points in the study were considered to be post-vaccine phase. Safety data was compared between the 2 arms of the study, while immune and PSA data were assessed by comparisons made between pre- and post-vaccine phases in all 24 patients.

#### Patient Selection and Vaccination

Patients were eligible to participate if they provided informed consent, had biopsy proven prostate cancer and progressive disease: PSA documented to be rising on 3 occasions, either despite castrate testosterone levels (below 50 ng/dl) or despite definitive local therapy (prostatectomy, radiation, etc.). Exclusion criteria included prior biologic therapy with dendritic cells, autoimmune disease, or significant major organ disease.

DC vaccines were given together with DTH panels and patients were closely observed for one hour. Patients returned to clinic at 48 hours, at which time they were examined clinically and their DTH responses were read. Vaccines were administered at a cell dose ranging from 2−10×10^6^ DCs/vaccination, given subcutaneously in the inner aspect of the upper arm, approximately 6–8 cm from the axillary lymph nodes. Patients received one priming and three biweekly booster vaccinations. During the first two injections, patients also received 2−10×10^6^ DC/KLH.

### Vaccine

Vaccine was manufactured in a BSL-2 facility maintained and independently audited to meet Good Tissue Practice specifications. To prepare autologous DCs, peripheral blood mononuclear cells (PBMCs) were obtained by leukapheresis, adhered to endotoxin free tissue-culture dishes (Falcon), and differentiated *in vitro* to immature DCs over six days in RPMI-1640 supplemented with 1% autologous plasma, GM-CSF (180 ng/ml; Bayer HealthCare Pharmaceuticals) and IL-4 (10 mg/ml; R&D Systems) as described[29], [39]. LNCaP prostate tumor cells were obtained from the American Type Cell Culture and a master cell bank was made and screened by BioReliance Corporation (Rockville, MD) to rule out contamination or adventitious agents. LNCaP cells from the master stock (frozen in Aim V media (Invitrogen) plus BSE-free fetal bovine serum (20% FBS; HyClone) and 5% DMSO (Edward Life Sciences)) were expanded in Aim V media/1% FBS, and cells were treated with UV-B irradiation such that >90% of cells underwent apoptotic death (Caspatag positive, as described[29]). Immature DCs and apoptotic LNCaP were co-cultured at a ratio of 1∶1 for 36–48 hours in the presence of PGE_2_ (20 mM; Sigma) and TNF-α (150 ng/ml; R&D Systems) to mature DCs as described[40]. DCs pulsed with KLH (2.5 µg/ml, biosyn) provided a positive control for DC function. All patients were also immunized with 2−10×10^6^ DCs cross-presenting apoptotic LNCaP producing the M1 antigen as a potential positive control (DC/LNCaP-M1), but only four patients were HLA A2.1^+^ and tetramer analysis did not show boosted M1 responses post vaccination in these patients (data not shown). The quality and viability of the vaccine was monitored by flow cytometry; to pass release criteria the HLA-DR (DC) cell fraction had to be >70% CD83+, <15% CD14+ and <20% Propidium Iodide (PI) + (Serologicals). All antibodies were purchased from BD Pharmingen. Sterility was tested by gram stain, by culture for bacterial and fungal contamination (BacT/ALERT, Biomereux), and by DNA fluorochrome for mycoplasma (Bionique Testing Laboratories, Inc.). The LAL method (Associates of Cape Cod) was used to test for endotoxin. The final product was divided into 4 equal aliquots, each containing 2−10×10^6^ DCs each. The first aliquot was given as a “fresh” dose; all other aliquots were frozen and thawed prior to administration.

### Clinical and Immunomonitoring

#### Clinical Monitoring

Patients were monitored at each study visit by history, physical examination, and by laboratory evaluations including CBC, chemistries, and urinalysis, for any adverse effects. The National Cancer Institute Common Toxicity Criteria version 3 was used to grade toxicities. Patients were also asked to maintain a diary of local injection site reactions and systemic adverse events for one week following each vaccination.

To assess for clinical response, PSA levels were measured in the serum using MSA Bayer Immuno I or IEA Tosoh Nexia assays at each study visit. The PSA slope was calculated for each phase of the study (pre-vaccine, vaccine, and post-vaccine phases) using a linear mixed model with two knots representing the change in slope in each phase of the study. In addition, for each phase of study for each patient, a PSA doubling time (PSADT) was calculated using the formula: 1 divided by the slope log_2_ PSA derived from this linear spline model.

#### Immunomonitoring

A DTH panel was placed intradermally at weeks 1 (vaccine), 3 (Boost #1), 5 (Boost #2), 7 (Boost #3), 9, 17 and 29 to assess for T cell responses. The panel included lysate of 10^5^ LNCaP cells (in 0.1 ml normal saline), 0.05 mg KLH, candida or tetanus toxoid (whichever the patient had responses to at baseline, as positive control), and saline (as negative control). Responses were considered positive if erythema was equal to or greater than 5 mm at 48 hours post implantation.

We evaluated immunogenicity outcomes against apoptotic LNCaP and PC3 (another prostate cancer tumor cell line) by ^3^H thymidine or CFSE proliferation assays as described[29] with the following modifications. In the ^3^H thymidine assay, autologous monocytes (CD14+ cells) were used as APCs. 3T3 cells, an irrelevant cell line, were used as the negative control antigen and influenza infected (strain A/P/R8) 3T3 cells were used as a positive control for both pre-vaccine and post-vaccine T cell responses. 3T3 cells were obtained from the American Type Cell Culture. In the CFSE assay, DCs cross-presenting prostate tumor antigens or 293 cells (irrelevant cell line used as negative control antigen) were used as APCs and cultured with T cells stained with CFSE (Vibrant CFDA SE Cell Tracer Kit, Invitrogen). On day 5, T cells were stained with anti-CD8 conjugated antibody (Becton Dickinson) and analyzed using FlowJo software. All cell lines were UV-B irradiated to induce apoptotic death three hours prior to co-culture with APCs[29].

To assess for correlations between patients who had a DTH response to LNCaP lysate and those who had T cell proliferation responses to apoptotic LNCaP *in vitro,* patients were ranked by a DTH index and a T cell (^3^H thymidine) proliferation index. The DTH index was calculated as the number of positive DTH responses to LNCaP lysate divided by the total number of DTH time points for each patient. The T cell proliferation index was calculated as the post-vaccine LNCaP response (CPM) minus 2 standard deviations, divided by post-vaccine 3T3 response (CPM) plus 2 standard deviations.

FACS staining of regulatory T cells was done by surface staining with anti-CD4-FITC (Becton Dickinson) followed by intracellular staining with anti-Foxp3-APC (eBioscience, clone PCH101) per manufacturers instructions. For analysis, PBMCs were gated on CD4+ T cells and the percentage of Foxp3+ cells was determined (FlowJo).

### Statistical Analysis

The protocol called for 12 patients to be recruited to each of two arms, which would have 90% power to detect an increase in cumulative serious adverse events (SAEs) from 5% in the placebo group to 50% in the vaccine group at the 10% level. All adverse events were analyzed using Fisher's exact test to compare placebo and vaccine phase. Significance of DTH responses to LNCaP cell lysate was determined by the exact binomial test with 95% confidence intervals. To analyze pre- to post- T cell proliferation data (per antigen) for study subjects as a group, a two-sampled paired t-test was used with 95% confidence intervals. The Spearman rank correlation test was used to determine if those that had DTH responses to LNCaP lysate also had T cell proliferation responses to apoptotic LNCaP *in vitro*. The paired t-test was used to compare the percentage of pre- vs post-vaccine Foxp3+ cells in PBMCs with 95% confidence intervals. In all of the above tests, the differences or correlation were deemed statistically significant if the p-value was less than 0.05.

To model the evolution of PSA (in log-scale) during the three study phases (pre-vaccine, vaccine, and post-vaccine phases), a mixed linear spline model was used. Two knots (one at the start of the vaccine phase and the other at the start of the post-vaccine phase) were used to directly quantify the differences in slopes between each phase. To account for the heterogeneous treatment effect and the repeated measures structure, random effects are incorporated into the model. For the general model, random effects for the intercept, slope and the first knot were considered. To determine if there was any difference in PSA slopes between those that did have an immunologic response (DTH responders to LNCaP lysate) and those that did not (DTH non-responders to LNCaP lysate), a second model, stratified by these response indicators (with separate knots for the two groups), was fitted and the likelihood ratio test was conducted. Similarly, a third model was fitted, this time, stratified by PSA at study start of <1 ng/ml or ≥1 ng/ml. The models with stratification were then compared to the general model. All models were fitted and hypotheses were tested using the *lme* package from R (www.R-project.org). The Wilcoxon matched-paired signed rank test was used to determine statistical significance of the difference in PSADT between phases (www.fon.hum.uva.nl/service/statistics/signed_rank_test.html). A PSADT can be a positive number as a result of rising PSA levels or a negative number as a result of declining PSA levels. For the purpose of statistical analysis, negative PSADT (indicating declining PSAs) were replaced with 9999 months to represent an infinitely long doubling time so that they rank higher than any value for which doubling time was a positive number. Differences were deemed significant if the p-value was less than 0.05.

## Supporting Information

Checklist S1 Consort checklist.
(0.13 MB DOC)

Protocol S1 FDA-approved IND 10710 protocol.
(0.58 MB DOC)
