# Supplementary material for: Harnessing Naturally Occurring Tumor Immunity: A Clinical Vaccine Trial in Prostate Cancer
Source: PLoS One. 2010 Sep 1;5(9):e12367. doi: 10.1371/journal.pone.0012367 (PMC2931687; doi:10.1371/journal.pone.0012367)
Supplement: Checklist S1 — Consort checklist. (0.13 MB DOC) [file pone.0012367.s001.doc]

# CONSORT Statement 2001 - Checklist
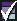


**Items to include when reporting a randomized trial**

| ***PAPER SECTION* And topic** | Item | **Descriptor** | **Reported in Section** |
| --- | --- | --- | --- |
| TITLE & ABSTRACT | 1 | [How participants were allocated to interventions](http://www.consort-statement.org/index.aspx?o=1107) (*e.g*., "random allocation", "randomized", or "randomly assigned"). | Title page and Abstract |
| *INTRODUCTION* Background | 2 | [Scientific background and explanation of rationale](http://www.consort-statement.org/index.aspx?o=1016). | Introduction |
| *METHODS* Participants | 3 | [Eligibility criteria for participants](http://www.consort-statement.org/index.aspx?o=1017#3a) and the [settings and locations where the data were collected](http://www.consort-statement.org/index.aspx?o=1017#3b). | Methods &  see below |
| Interventions | 4 | [Precise details of the interventions intended for each group and how and when they were actually administered](http://www.consort-statement.org/index.aspx?o=1021). | Methods |
| Objectives | 5 | [Specific objectives and hypotheses](http://www.consort-statement.org/index.aspx?o=1022). | Introduction & see below |
| Outcomes | 6 | [Clearly defined primary and secondary outcome measures](http://www.consort-statement.org/index.aspx?o=1023#6a) and, when applicable, any [methods used to enhance the quality of measurements](http://www.consort-statement.org/index.aspx?o=1023#6b) (*e.g.*, multiple observations, training of assessors). | Introduction& see below |
| Sample size | 7 | [How sample size was determined](http://www.consort-statement.org/index.aspx?o=1024#7a) and, when applicable, [explanation of any interim analyses and stopping rules](http://www.consort-statement.org/index.aspx?o=1024#7b). | Methods & see below |
| Randomization -- Sequence generation | 8 | [Method used to generate the random allocation sequence, including details of any restrictions](http://www.consort-statement.org/index.aspx?o=1025) (*e.g*., blocking, stratification) | See below |
| Randomization -- Allocation concealment | 9 | [Method used to implement the random allocation sequence](http://www.consort-statement.org/index.aspx?o=1026) (*e.g*., numbered containers or central telephone), clarifying whether the sequence was concealed until interventions were assigned. | See below |
| Randomization -- Implementation | 10 | [Who generated the allocation sequence, who enrolled participants, and who assigned participants to their groups](http://www.consort-statement.org/index.aspx?o=1027). | See below |
| Blinding (masking) | 11 | [Whether or not participants, those administering the interventions, and those assessing the outcomes were blinded to group assignment](http://www.consort-statement.org/index.aspx?o=1028#11a). If done, [how the success of blinding was evaluated](http://www.consort-statement.org/index.aspx?o=1028#11b). | See below |
| Statistical methods | 12 | [Statistical methods used to compare groups for primary outcome(s)](http://www.consort-statement.org/index.aspx?o=1029#12a); [Methods for additional analyses](http://www.consort-statement.org/index.aspx?o=1029#12b), such as subgroup analyses and adjusted analyses. | Methods |
| RESULTS Participant flow | 13 | [Flow of participants through each stage](http://www.consort-statement.org/index.aspx?o=1018) (a diagram is strongly recommended). Specifically, for each group report the numbers of participants randomly assigned, receiving intended treatment, completing the study protocol, and analyzed for the primary outcome. [Describe protocol deviations from study as planned, together with reasons](http://www.consort-statement.org/index.aspx?o=1086). | Results & see below |
| Recruitment | 14 | [Dates defining the periods of recruitment and follow-up](http://www.consort-statement.org/index.aspx?o=1087). | Methods & see below |
| Baseline data | 15 | [Baseline demographic and clinical characteristics of each group](http://www.consort-statement.org/index.aspx?o=1088). | Results & see below |
| Numbers analyzed | 16 | [Number of participants (denominator) in each group included in each analysis and whether the analysis was by "intention-to-treat"](http://www.consort-statement.org/index.aspx?o=1089). State the results in absolute numbers when feasible (*e.g*., 10/20, not 50%). | Results |
| Outcomes and estimation | 17 | [For each primary and secondary outcome, a summary of results for each group, and the estimated effect size and its precision](http://www.consort-statement.org/index.aspx?o=1090) (*e.g.*, 95% confidence interval). | Results |
| Ancillary analyses | 18 | [Address multiplicity by reporting any other analyses performed](http://www.consort-statement.org/index.aspx?o=1091), including subgroup analyses and adjusted analyses, indicating those pre-specified and those exploratory. | Results |
| Adverse events | 19 | [All important adverse events or side effects in each intervention group](http://www.consort-statement.org/index.aspx?o=1092). | Results |
| *DISCUSSION* Interpretation | 20 | [Interpretation of the results](http://www.consort-statement.org/index.aspx?o=1019), taking into account study hypotheses, sources of potential bias or imprecision and the dangers associated with multiplicity of analyses and outcomes. | Discussion |
| Generalizability | 21 | [Generalizability (external validity) of the trial findings](http://www.consort-statement.org/index.aspx?o=1094). | See below |
| Overall evidence | 22 | [General interpretation of the results in the context of current evidence](http://www.consort-statement.org/index.aspx?o=1095). | Discussion |

**Item #3: Eligibility criteria for participants**

##### --Disease Characteristics--

1. Histologically confirmed prostate carcinoma

2. Progressive, disease required, i.e.: elevated PSA documented to be rising on 3 occasions, either despite castrate testosterone levels (below 50 ng/dl), or after definitive local therapy (prostatectomy or radiation).

| Extent of disease | Min # of values | Interval |
| --- | --- | --- |
| Rising PSA within 2 years of prostatectomy | 3 | >2 weeks |
| Rising PSA post-radiation^*^ | 3 | >2 weeks |
| Rising PSA despite castrate testosterone | 3 | >2 weeks |

^*^Baseline is defined as the first of three rising values confirmed after definitive radiation treatment.

3. No active CNS metastases

##### --Prior/Concurrent Therapy--

1. Recovered from toxicity of any prior therapy

2. Biologic therapy:

No prior autologous or allogeneic tumor vaccines

No concurrent other immunotherapy

3. Chemotherapy:

At least 4 weeks since chemotherapy

Not previously treated with more than 2 chemotherapy regimens

No concurrent chemotherapy

4. Endocrine evaluation/therapy:

3 rising PSA values at least 2 weeks apart

At least 2 weeks since concurrent corticosteroids (other than for replacement therapy for adrenal insufficiency)

Medical hormonal therapy to maintain castrate testosterone levels permitted

5. Radiotherapy:

At least 4 weeks since radiotherapy

No concurrent radiotherapy

6. Surgery:

Prior surgery allowed

##### --Patient Characteristics--

1. Age: 18 and over, able to give written informed consent. Individuals unable to provide informed consent must have consent provided by the legal guardian, or person designated by the subject to give consent on his behalf.

2. Performance status: Karnofsky 70-100%

3. Life expectancy: At least 1 year

4. Hematopoietic: obtained twice, once within 45 days prior to study entry, and again within 72 hours of study entry.

WBC greater than 3,800

Absolute neutrophils greater than 1,500

Absolute lymphocytes greater than 500

Platelets greater than 120,000

Hb at least 10 g/dl

5. Hepatic:

Bilirubin less than 2.0 mg/dl OR

SGOT less than 2 x ULN

6. Renal:

Creatinine no greater than 2.0 mg/dl OR

Creatinine clearance at least 40 ml/min

7. Rheumatologic:

ANA no greater than upper limit of normal, or ANA abnormal in absence of clinical signs of autoimmunity.

Rheumatoid factor (RF) no greater than upper limit of normal, or RF abnormal in absence of clinical signs of autoimmunity.

Anti-ds DNA no greater than upper limit of normal, or anti ds DNA abnormal in absence of clinical signs of autoimmunity.

1. Immunologic:

Influenza serology (assessment made at time of screening).

Assessment of DTH response to a standard anergy panel (to include candida, trichophyton and tetanus) or to a Multitest CMI (a disposable kit for DTH testing with standardized preloaded antigens).

1. Cardiovascular:

No NYHA class III/IV status

No active angina, clinically significant cardiac arrythmia, recent (6 months) myocardial infarction

1. Pulmonary:

No severe debilitating pulmonary disease

1. Endocrine:

TSH, T3, and T4 no greater than upper limit of normal

1. Radiographic:

Baseline bone scan

Baseline CT or MRI of abdomen and pelvis

1. Other:

No active infection requiring antibiotics

No active pain requiring chronic opioid analgesics.

Not HIV, hepatitis B or hepatitis C virus positive; anti-HIV, HbsAg and Hep C antibody negative

No history of hypersensitivity to vaccine components

No serious uncontrolled medical illness

No currently active second malignancy other than non-melanoma skin cancer (note: a patient is NOT considered to have currently active malignancy if they have completed therapy and are now considered by their physician to be at less than 30% risk for relapse)

No history of total lymph node irradiation

No history of vasculitis, including but not limited to systemic necrotizing vasculitides (polyarteritis nodosa group), hypersensitivity vasculitis, Wegener’s granulomatosis.

No history of autoimmune disease.

No use of hydroxyurea within 45 days of study entry

No receipt of immune modulators or suppressors within 30 days prior to study entry, including but not limited to interferons and thalidomide. No active requirement for corticosteroids; prior use is acceptable.

No psychiatric illness or social condition that, in the opinion of the investigator, would interfere with adherence to study requirements.

No alcohol or drug use or dependence that, in the opinion of the investigator, would interfere with adherence to study requirements.

**Item #5 Specific objectives and hypotheses**

The primary objective in study is to ascertain the safety of the DC/LNCaP vaccine. More precisely, the study monitored the safety of employing *ex vivo* generated autologous dendritic cells (DCs) pulsed with apoptotic prostate cancer cells as a therapeutic in prostate cancer patients.

An additional objective of this study is to evaluate the immunogenicity of autologous DCs pulsed with apoptotic prostate cancer cells.

Secondary objectives in this study are to evaluate the clinical course of immunized patients by measuring PSAs before, during, and after vaccine.

**Item #6: Clearly defined primary and secondary outcome measures**

The primary outcomes are the evaluation of adverse events related to the vaccine and evaluation of the immunogenicity of the vaccine by T cell proliferation assays and ELISPOT assays.

Secondary outcome measures are change in PSA slopes and PSA doubling times before, during, and after vaccination

**Methods used to enhance the quality of measurements**

Self-Assessment Questionnaires were completed by the patient to document adverse events daily for 7 days after each injection to minimize unreported adverse events or inaccurately reported adverse events by patients.

Proliferation assays and FACS analysis were performed by trained personnel using written SOP to ensure quality control.

PSAs were measured in a CLIA certified laboratory.

**Item #7: Explanation of any interim analyses and stopping rules**

Preliminary safety analysis was done after each vaccination.

Criteria for Subject Study Discontinuation

- Development of an exclusionary condition as outlined in “eligibility criteria”, prior to or during vaccination, with the following exceptions:
  - a course of palliative radiation treatment during the study
- platelet counts that drop > 50% from baseline.
  - hemoglobin that drops sufficiently to require transfusion.
  - if the patient develops an infection requiring treatment as an inpatient.
- Failure by the subject to attend three consecutive clinical study visits.
- Request by the subject to withdraw.
- Clinical reasons judged by the study physician to be life threatening, even if not addressed in the toxicity management of the protocol.
- Completion of week 17 or in some patients, week 29 in Arm 1.
- Completion of week 25 or in some patients week 37 in Arm 2.
- Any dose limiting toxicity. A dose limiting toxicity is defined as:
  1. any grade 2 or greater allergic toxicity or asymptomatic bronchospasm or generalized urticiaria
  2. any grade 3 or greater toxicity (hematologic or non-hematologic)
  3. any grade 2 or greater autoimmune reactions

The study will be stopped if 1 of 3, 2 of 6, 3 of 9, or 4 of 12 subjects experience dose limiting toxicity as defined under Criteria for Subject Study Discontinuation.

**Item #8: Method used to generate the random allocation sequence**

Randomization was done using a computer-generated randomization list in blocks of 4 participants.

**Item #9: Method used to implement the random allocation sequence**

Patients were screened and were ready for enrollment if eligibility criteria were met. The patient was then assigned to one of two arms according to the randomization list. The assignment was made by a person who had no other study related responsibilities.

**Item #10: Who generated the allocation sequence, who enrolled participants, and who assigned participants to their groups**

The study nurse practitioner screened the participants and determined eligibility. A person who had no other study related responsibilities generated the allocation sequence and assigned eligible participants to their groups according to this sequence. However, as this was a single-blind study, after assignment to an arm, the study nurse practitioner knew which arm the patient is in during their participation.

**Item #11: Whether or not participants, those administering the interventions, and those assessing the outcomes were blinded to group assignment**

The participants were blinded. As this was a single blind study, those administering the interventions and those assessing the outcomes were not blinded.

**How success of blinding was evaluated**

Success of blinding was not evaluated.

**Item #13: Describe protocol deviations from study as planned, together with reasons**

| **No.** | **Pt.** | **Date** | **Deviation** |
| --- | --- | --- | --- |
| 1 | 02 | 5/3/04 | Pt will not have 100cc of research blood drawn. He was slightly anemic at baseline, and is worsening as we draw blood, though no change in CTC grade. As he is still in the placebo phase, this will not result in a major data collection compromise. |
| 2 | 04 | 5/4/04 | Pt is re-scheduled for Day 7 instead of Day 8 post leukapheresis as Day 8 falls on a day that the hospital is having an all day fire alarm. Pt is to receive placebo injections on Day 7. This change does not worsen any risks to patient or compromise data collection. |
| 3 | 04 | 5/4/04 | On the Week 1 visit, 100 cc research bloods were ordered and drawn, when only safety bloods should have been ordered and drawn at this visit. |
| 4 | 02 | 6/14/04 | No research bloods (100cc) drawn for week 13 per protocol. He had his week 12 visit only 3 days before and had 100cc drawn then. Not drawn because of the anemia and the close time frame. |
| 5 | 04 | 7/27/04 | Booster #1 given 2 days out of +/-3 days window due to travel plans that the patient had |
| 6 | 07 | 10/4/04 | Booster #2 given 5 days out of +/- 3 days window due to patient’s schedule. |
| 7 | 12 | 10/4/04 | Pt is having BS/CT done ON Day 0, AFTER pre treatment leukapheresis, instead of before due to scheduling issues. This will not affect outcome as this is still prior to first treatment. |
| 8 | 06 | 7/30/04 | Pt lost Post Vaccine Questionnaire. Any symptoms that occurred documented as part of history at next visit. |
| 9 | 11 | 10/18/04 | Post Vaccine Questionnaire not given out to patient in error. Any symptoms that occurred documented as part of history at next visit. |
| 10 | 10 | 10/13/04 | Due to short time between Placebo 2 and 3, no week 4 visit done. |
| 11 | 09 | 10/7/04 | Microscopic with UA ordered but not done. |
| 12 | 12 | 9/13/04 | Baseline ANA ordered at screen but not done. Drawn on 10/4/04, Day 0, prior to leukapheresis. |
| 13 | 12 | 10/14/04 | No doses of KLH left. No baseline KLH DTH given. |
| 14 | 07 | 10/14/04 | Post Vaccine Questionnaire not given out to patient. Any symptoms that occurred documented as part of history at next visit. |
| 15 | 09 | 10/26/04 | Repeat leukapheresis due to contamination of first leukopack. |
| 16 | 12 | 11/15/04 | Due to patient travel schedule, all of the DTHs are read at 24 instead of 48 hours post planting. |
| 17 | 16 | 1/10/05 | Post Vaccine Questionnaire not given out to patient. Any symptoms that occurred documented as part of history at next visit. |
| 18 | 14 | 2/17/05 | Baseline BS and CT done one day after 1^st^ vaccine injection, due to travel and scheduling problems |
| 19 | 16 | 3/10/05 | Pt missed Placebo 4 DTH reading appointment |
| 20 | 17 | 4/25/05 | Week 5 research blood not ordered and not drawn |
| 21 | 17 | 4/27/05 | Patient did not complete Self Assessment Questionnaire. Verbal history only |
| 22 | 17 | 4/20/05 | Week 4 visit not scheduled due to difficultly in travel from Kentucky |

**Item #14: Dates defining periods of follow-up**

The last patient completed the study in August 2006.

**Item #15: Baseline demographic and clinical characteristics of each group**

| **Characteristics** | **Arm 1 Vaccine (n=12)** | **Arm 2 Placebo, then Vaccine (n=12)** |
| --- | --- | --- |
| Mean age ± SD, years | 62.5 ± 6.7 | 65.7 ± 9.2 |
| Mean PSA at start of study ± SD, ng/ml | 4.7 ± 11.3 | 4.3 ± 4.5 |
| No. pts with biochemical relapse only at start of study | 11 | 10 |

Note: Because this is a cross over study, all patients in Arm 2 who initially had placebo eventually received vaccine in the same way that those in Arm 1 did. The immunological and clinical results are therefore based on data generated from all 24 patients put together. We compare the placebo and vaccine groups only in determining what are vaccine related adverse events.

**Item #21: Generalizability (external validity) of the trial findings**

Although not a requirement for enrollment, most of the participants had a relative low PSA at the start of the study and only had biochemical relapse and low tumor burden. These results do not adequately address patients with higher PSAs and who have evidence of clinical metastasis.
