# Supplementary material for: Harnessing Naturally Occurring Tumor Immunity: A Clinical Vaccine Trial in Prostate Cancer
Source: PLoS One. 2010 Sep 1;5(9):e12367. doi: 10.1371/journal.pone.0012367 (PMC2931687; doi:10.1371/journal.pone.0012367)
Supplement: Protocol S1 — FDA-approved IND 10710 protocol. (0.58 MB DOC) [file pone.0012367.s002.doc]

# Cover sheet (From FDA-1571)

See attached FDA-1571

# Table of Contents

(1) Cover sheet (From FDA-1571) [1](#__RefHeading___Toc430860998)

(2) Table of Contents [1](#__RefHeading___Toc430860999)

(3) Introductory statement [4](#__RefHeading___Toc430861000)

i. Introduction to Proposal [4](#__RefHeading___Toc430861001)

ii. Drug Information [4](#__RefHeading___Toc430861002)

iii. Broad Objectives and Planned Duration of Clinical Investigation [5](#__RefHeading___Toc430861003)

a. Broad Objectives [5](#__RefHeading___Toc430861004)

b. Planned Duration [6](#__RefHeading___Toc430861005)

c. Clinical development plan [6](#__RefHeading___Toc430861006)

iv. Previous human experience/other INDs [7](#__RefHeading___Toc430861007)

a. Dendritic cell vaccines [7](#__RefHeading___Toc430861008)

b. KLH vaccines [7](#__RefHeading___Toc430861009)

v. Previous drug withdrawal. [7](#__RefHeading___Toc430861010)

(4) General investigational plan [8](#__RefHeading___Toc430861011)

i. Overall Plan for following year [8](#__RefHeading___Toc430861012)

ii. Rationale for drug/research study [8](#__RefHeading___Toc430861013)

iii. Indications to be studied [8](#__RefHeading___Toc430861014)

iv. General approach in evaluating the drug [8](#__RefHeading___Toc430861015)

v. Kind of clinical trial: Project Phase [8](#__RefHeading___Toc430861016)

vi. Estimated number of patients [9](#__RefHeading___Toc430861017)

vii. Risks of particular severity based on animal/prior human studies [9](#__RefHeading___Toc430861018)

(5) Investigator’s Brochure. [9](#__RefHeading___Toc430861019)

(6) Protocols [10](#__RefHeading___Toc430861020)

i. Study Protocol [10](#__RefHeading___Toc430861021)

a. Face Sheet [10](#__RefHeading___Toc430861022)

b. Précis [12](#__RefHeading___Toc430861023)

c. Background [12](#__RefHeading___Toc430861024)

d. Objectives [14](#__RefHeading___Toc430861025)

e. Subjects [15](#__RefHeading___Toc430861026)

f. Vaccines [18](#__RefHeading___Toc430861027)

g. Study Design [19](#__RefHeading___Toc430861028)

h. Outcome Parameters [25](#__RefHeading___Toc430861029)

i. Monitoring and follow-up. [30](#__RefHeading___Toc430861030)

j. Benefits [33](#__RefHeading___Toc430861031)

k. Risks and complications [33](#__RefHeading___Toc430861032)

l. Withdrawal from study [35](#__RefHeading___Toc430861033)

m. Stopping rules [35](#__RefHeading___Toc430861034)

n. Data analysis [38](#__RefHeading___Toc430861035)

o. Medical care [41](#__RefHeading___Toc430861036)

p. Data accuracy and protocol compliance. [41](#__RefHeading___Toc430861037)

q. Consent form [43](#__RefHeading___Toc430861038)

ii. Investigator Data [43](#__RefHeading___Toc430861039)

iii. Facilities Data [43](#__RefHeading___Toc430861040)

iv. Institutional Review Board Data [43](#__RefHeading___Toc430861041)

(7) Chemistry, manufacture & control [44](#__RefHeading___Toc430861042)

i. Chemistry, Manufacturing and Control Data [44](#__RefHeading___Toc430861043)

a. Drug Name [44](#__RefHeading___Toc430861044)

b. Quality/Ingredients [44](#__RefHeading___Toc430861045)

c. Biologic characteristics [44](#__RefHeading___Toc430861046)

ii. Flow diagram—Vaccine Preparation [45](#__RefHeading___Toc430861047)

iii. Drug substance [46](#__RefHeading___Toc430861048)

a. LNCaP Tumor Cells [46](#__RefHeading___Toc430861049)

b. LNCaP-M1 Master Cell Bank [48](#__RefHeading___Toc430861050)

c. Autologous DCs [49](#__RefHeading___Toc430861051)

d. Acceptable limits and analytical methods to assure identity, strength, quality, purity [50](#__RefHeading___Toc430861052)

iv. General method of vaccine preparation [52](#__RefHeading___Toc430861053)

a. DC/LNCaP [52](#__RefHeading___Toc430861054)

b. DC/LNCaP-M1 [53](#__RefHeading___Toc430861055)

v. Drug product [53](#__RefHeading___Toc430861056)

a. Components [53](#__RefHeading___Toc430861057)

b. Quantitative composition of Drug Product [53](#__RefHeading___Toc430861058)

c. Qualitative composition of Drug Product [54](#__RefHeading___Toc430861059)

d. Manufacturer [54](#__RefHeading___Toc430861060)

e. Manufacturing/packaging procedure [54](#__RefHeading___Toc430861061)

f. Acceptable limits and analytical methods to assure identity, strength, quality, purity [55](#__RefHeading___Toc430861062)

vi. Lot release summary. [56](#__RefHeading___Toc430861063)

a. Lot release tests. [56](#__RefHeading___Toc430861064)

vii. Placebo Identity [58](#__RefHeading___Toc430861065)

a. Description of placebo [58](#__RefHeading___Toc430861066)

viii. Labeling [58](#__RefHeading___Toc430861067)

ix. Environmental analysis requirement [59](#__RefHeading___Toc430861068)

(8) Pharmacology/Toxicology information [59](#__RefHeading___Toc430861069)

i. Safety studies [59](#__RefHeading___Toc430861070)

a. Animal studies to evaluate drug toxicity [59](#__RefHeading___Toc430861071)

b. In vitro studies to evaluate drug toxicity [62](#__RefHeading___Toc430861072)

c. Additional pre-clinical toxicity studies. [62](#__RefHeading___Toc430861073)

ii. Activity studies [63](#__RefHeading___Toc430861074)

a. Animal efficacy data. [63](#__RefHeading___Toc430861075)

b. Human efficacy data. [63](#__RefHeading___Toc430861076)

iii. Tabulation of data suitable for detailed review [64](#__RefHeading___Toc430861077)

iv. Statement that study is conducted in compliance with good lab practice [65](#__RefHeading___Toc430861078)

(9) Previous human experience with the investigational drug [65](#__RefHeading___Toc430861079)

i. Previous investigation/marketing [65](#__RefHeading___Toc430861080)

ii. Previous experience with components [65](#__RefHeading___Toc430861081)

a. Safety and efficacy of DC and tumor cell vaccines. [65](#__RefHeading___Toc430861082)

b. Prostate cancer: safety and efficacy of DC and tumor vaccines [66](#__RefHeading___Toc430861083)

c. Detailed information on previous trial relevant to assessment of effectiveness. [66](#__RefHeading___Toc430861084)

d. Detailed Bibliography [67](#__RefHeading___Toc430861085)

(10) Appendices [72](#__RefHeading___Toc430861086)

Appendix I. Drug formus [72](#__RefHeading___Toc430861087)

**List of Figures**

Figure 1. Flow diagram for Vaccine production and testing. [45](#__RefHeading___Toc430925920)

Figure 2. Validation of LNCaP cell identity. [46](#__RefHeading___Toc430925921)

Figure 3. Lack of detectable viability in apoptotic LNCaP cells. [47](#__RefHeading___Toc430925922)

Figure 4. Quality assessment of DCs grown from a prostate cancer patient. [50](#__RefHeading___Toc430925923)

Figure 5. Apoptotic LNCaP cells are replication incompetent. [62](#__RefHeading___Toc430925924)

Figure 6. Mouse DCs cross-presenting apoptotic antigen block tumor growth. [63](#__RefHeading___Toc430925925)

**List of Tables**

Table 1. Comparison of immunization strategies [14](#__RefHeading___Toc430926519)

Table 2. Schedule of Events. [22](#__RefHeading___Toc430926520)

Table 3. Grading and Attribution of Local Reactions to Vaccine [37](#__RefHeading___Toc430926521)

Table 4: Lot Release Summary [57](#__RefHeading___Toc430926522)

Table 5. Animal Histology Summary [60](#__RefHeading___Toc430926523)

Table 6. Tabulation of Human Efficacy Data. [64](#__RefHeading___Toc430926524)

# Introductory statement

## Introduction to Proposal

Our laboratory has been studying how naturally occurring, effective tumor immunity works in a rare set of human cancer patients, and now proposes studies aimed at generalizing our findings to prostate cancer patients. We have found that patients with a rare set of neurologic disorders (the paraneoplastic neurologic degenerations; PNDs) have clinically effective tumor immunity and harbor tumor antigen-specific killer T cells. We have extended this observation to identify a new physiologic pathway by which these killer T cells become activated. In this pathway, apoptotic tumor cells are phagocytosed by dendritic cells (DCs), leading to efficient tumor antigen presentation on DCs and the activation of potent tumor-specific CD8^+^ and CD4^+^ T cells.

The approach is particularly relevant to prostate cancer because both hormonal therapy and chemotherapy result in the clearance of only a proportion of the most actively dividing tumor cells, while the majority of the remaining cells persist in a growth arrested state that we hypothesize will be sensitive to immunotherapy.

We propose a clinical immunotherapy trial to apply our findings to patients with advanced prostate cancer. Prostate cancer is the second leading cause of cancer-related death in men, and a large body of work has identified excellent markers for both tumor burden and T cell responses to common prostate cancer antigens.

In this Phase I/II clinical trial, we will use apoptotic material derived from prostate cancer cell lines to transfer prostate cancer antigens *ex vivo* to autologous DCs obtained from patients. These antigen-loaded DCs will be injected subcutaneously, and patients will be monitored for both clinical and immunologic responses.

We have developed assays that allow us to monitor both CD8^+^ and CD4^+^ T cell responses to our immunizations in patients of both HLA A2.1 and other HLA haplotypes.

**Hypothesis**

Patients with advanced prostate cancer can be safely immunized with autologous DCs that have been pulsed with apoptotic prostate tumor cells.

## Drug Information

**Name of Drug**

**DC/LNCaP:** Autologous dendritic cells that have been co-cultured with apoptotic LNCAP cells.

**DC/LNCAP-M1:** Autologous dendritic cells that have been co-cultured with apoptotic LNCAP cells that are stably transfected with a plasmid cDNA expressing the influenza matrix antigen.

**DC/KLH:** Autologous dendritic cells that have been exposed to KLH antigen.

**Pharmacological class**: Biologic.

**Formulation**: Cells will be washed and resuspended in 5% DMSO and 25% autologous serum in sterile saline for injection.

**Route of administration**: *Ex vivo* generated autologous cells that have been pulsed with apoptotic tumor cells will be injected into the subcutaneous tissue of the upper arm or thigh with a tuberculin syringe.

## Broad Objectives and Planned Duration of Clinical Investigation

### Broad Objectives

There is currently no standard therapy for patients with prostate cancer who have completed conventional treatment modalities and then develop rising prostate-specific antigen (PSA) levels. We will leukapherese this group of prostate cancer patients to generate autologous dendritic cells (DCs). These will be incubated with apoptotic material from prostate cancer cell lines, and then re-introduced to the patients by subcutaneous injection to stimulate prostate tumor immune responses mediated by tumor antigen-specific killer T cells. We will monitor the patients’ immune and clinical status, including assays to determine whether this protocol leads to any adverse allergic or autoimmune reaction. To determine that the immunization was active, we will monitor delayed type hypersensitivity (DTH) skin tests. As an additional assay for the activity of our vaccine approach, patients will also be immunized with DCs that have been co-cultured with prostate tumor cell lines stably transfected with the influenza matrix antigen (LNCaP-M1 cells). This will allow us to monitor responses to a built-in “positive control” antigen by examining T cell reactivity to the influenza matrix antigen. By determining vaccine activity, we will be able to make robust conclusions regarding vaccine toxicity.

To help control for events related to the natural history of prostate cancer patients during the course of the trial, half of our patients will randomly be placed into a placebo arm of the study, in which they will receive either saline or the vaccine vehicle only injections (5% DMSO in sterile saline) subcutaneously during the first 8 weeks of the trial, and then cross-over to be immunized on a schedule identical to patients who receive the autologous DC vaccine up front. This trial design will aid us in assessing whether adverse events are treatment related in our cohort of cancer patients.

#### Schema

A Phase I/II Study to evaluate the toxicity and activity of the subcutaneous administration of autologous DCs pulsed with apoptotic prostate tumor cells in prostate cancer patients

##### Design

A phase I/II, randomized, single blind study. Subjects with prostate cancer with rising PSA levels who have failed conventional therapy are eligible.

A total of 24 patients will be enrolled into one of two Arms of 12 patients each. Subjects will be randomly assigned (1:1) at entry to one of the two vaccination groups.

Patients in Arm 1 will receive one immunization and 3 booster immunizations over the course of the first 7 weeks of study. Patients in Arm 2 will receive a placebo (either saline or vaccine vehicle only (5% DMSO in normal saline) immunization and 3 placebo booster immunizations, and will switch after week 7 to the vaccination arm, where they will receive one immunization and 3 booster immunizations exactly as for patients in Arm 1.

##### Regimen

Arm 1: Subcutaneous (SQ) vaccination with DCs co-cultured with apoptotic LNCaP and LNCaP-M1 tumor cells at weeks 1 and 3, 5 and 7, DCs exposed to keyhole limpet hemocyanin (KLH) at weeks 1 and 3, and either saline or vaccine vehicle only (5% DMSO in normal saline ) at weeks 1, 3, 5 and 7.

Arm 2: SQ vaccination of placebo (saline or vaccine vehicle only (5% DMSOin normal saline)) at weeks 1, 3, 5 and 7, followed by SQ vaccination with DCs co-cultured with apoptotic LNCaP and LNCaP-M1 tumor cells at weeks 9, 11, 13 and 15, DCs exposed to keyhole limpet hemocyanin (KLH) at weeks 9 and 11, and saline or vaccine vehicle only (5% DMSO andin normal saline ) at weeks 9, 11, 13, and 15.

Subjects in Arm 1 will undergo leukapheresis at entry and 6 weeks following the final DC vaccination. Subjects in Arm 2 will undergo leukapheresis at entry, after the placebo injections at Week 8 and 6 weeks following the final DC vaccination.

### Planned Duration

Immunizations will be given over the course of 7 weeks. The longest duration of follow-up will be at 41 weeks after study entry.

### Clinical development plan

The current studies are planned as a basic investigation into the toxicity and activity of apoptotic LNCaP cells pulsed on to autologous DCs. This vaccine is prepared in an academic laboratory, and our plans are to limit studies of this vaccine approach to small Phase I/II trials. If the results are to be extended in larger studies, it would be after transfer of this technology, for example to an interested pharmaceutical or biotechnology company.

Upon a positive outcome of the proposed protocol, a long term planned extension of this vaccination approach will be to examine whether antigen spread can be documented during such immunizations, i.e. reactivity initiated against tumor vaccine spreading to reactivity against antigen expressed by the patient’s tumor. One means of examining this question will be to undertake studies similar to those proposed here, but using the prostate tumor cell line PC3. This cell line, similar in many respects to LNCaP cells, does not express the prostate tumor antigens PSA or PSMA. Thus evidence of immunogenicity against these antigens following immunization with PC3/DC vaccine would provide evidence for antigenic spread to endogenous tumor antigens.

Another extension of these studies is to compare the efficacy of a tumor cell line/DC vaccine vs. an autologous tumor specimen/DC vaccine. Immunologic arguments for and against either strategy may only be settled by clinical trial. For such studies, we would extend the current IRB to include a glioblastoma cell line, which would be exposed to apoptotic stimulus and pulsed onto autologous DCs. This would be directly compared to immunization with autologous apoptotic glioblastoma tumor tissue pulsed onto autologous DCs.

## Previous human experience/other INDs

### Dendritic cell vaccines

We have extensive experience at the Rockefeller University Hospital with the use of autologous DCs charged with a variety of peptide antigens, under Rockefeller University IND #6756 (Principle Investigator: Nina Bhardwaj). There have been no serious adverse events or other significant safety issues arising during these studies, which bear many similarities to the current study. Autologous DCs were generated following leukapheresis and peripheral blood monocytes were isolated as planned here. These cells were isolated and differentiated into immature DCs in vitro (Dhodapkar et al., 2000; Dhodapkar et al., 1999). There has been no prior human experience with DC/LNCAP or DC/LNCAP-M1.

Prior studies of the use of DCs pulsed with tumor cells for the immunization of cancer patients used different means of killing tumor cells, which were intended to induce necrotic rather than apoptotic death. These studies thus differ in a substantive way from our current plans, most importantly differing in their ability to transfer antigens to DCs for the activation of T cells. Nonetheless, such studies have demonstrated the feasibility and safety of using tumor cells together with DCs for immunization. For example, in one study, autologous renal cancer cells were killed and incubated with allogeneic DCs prior to subcutaneous injection (Kugler et al., 2000), and in another study killed autologous glioblastoma cells have been used together with autologous DCs for immunization (University of Pittsburgh IND #8918) (Okada et al., 2001; Yu et al., 2001). Finally, children with solid tumors have been treated with tumor-lysate-pulsed dendritic cells (University of Michigan IND #BB6958) (Geiger et al., 2000; Geiger et al., 2001). In all reported cases, no serious acute toxicity has been noted in these studies.

### KLH vaccines

KLH, a copper-containing glycoprotein from the keyhole limpet *Megathura crenulata*, has been frequently used to stimulate potent immune responses in humans and animals to a neo-antigen. KLH has induced no toxicity in single injections in rodents in doses of 12.5 mg in the bladder, 0.1mg subcutaneously, or 5 mg submucosally (Klippel et al., 1977). Priming with lower doses followed by challenge with 5 mg KLH into the bladder mucosa led to inflammatory changes, without ulceration or necrotizing lesions, which resolved within 10 weeks.

In humans, KLH has been widely used to induce DTH and antibody responses. No adverse systemic or local reactions were reported in healthy humans immunized with 0.1 to 5 mg of KLH (Curtis et al., 1971). All 14 subjects immunized intradermally or subcutaneously with a single 0.1 mg or 5 mg KLH dose developed detectable antibodies by day 7. In another study, 13 of 19 subjects given a sensitizing dose of 5 mg KLH SQ plus an intradermal injection of 100 mg 5 to 7 days later developed DTH reactions to the protein, and no serious adverse reactions were reported (Olsson et al., 1972). Thus the use of KLH has a significant safety record in human vaccine trials.

## Previous drug withdrawal.

None.

# General investigational plan

## Overall Plan for following year

In the first 12 months of this study we expect to accrue 16 patients, approximately 8 in each Arm of the study.

## Rationale for drug/research study

Prostate cancer patients with high-grade tumors who develop rising PSA levels despite castrate hormone levels, or who develop biochemical relapse early after initial therapyhave a poor clinical prognosis (Pound et al., 1999; Scher, 1999; Scher, 2003; Slovin and Scher, 1999). Our laboratory has demonstrated that effective tumor immunity in humans is associated with, and likely mediated at least in part by tumor antigen-specific killer T cells (Albert et al., 1998a; Darnell, 1999; Darnell and Posner, 2003). Moreover, we have demonstrated that apoptotic material derived from dying tumor cells are a potent means of delivering antigen to DCs and subsequently triggering tumor antigen-specific T cell responses ex vivo (Albert et al., 1998a; Albert et al., 1998c).

We believe that these observations should be carefully followed up, testing safety and immunogenicity of using this pathway of antigen presentation to stimulate tumor immune responses in a clinical Phase I trial. We propose to generate apoptotic prostate tumor cells *in vitro*. We will obtain DCs from prostate cancer patients, co-culture them with apoptotic prostate tumor cells to allow antigen capture, processing and presentation on MHC. The antigen loaded DCs will then be injected back into patients to allow activation of tumor antigen-specific killer T cells.

## Indications to be studied

Patients with pathologically confirmed prostate cancer, progressive disease, who have no active CNS metastases or vital organ compromise, ages 18 years of age and older, available for up to 41 weeks of follow-up from the time of screening.

## General approach in evaluating the drug

The primary outcomes to be evaluated are toxicity and activity. Safety measures will include evaluation of both local and systemic toxicity. Immunogenicity will include evaluation of a number of standard parameters of immune response to the vaccine, such as DTH response, as well as a number of studies specific to this trial. Standard evaluation will include responses to the positive control immunogen KLH, and responses to the non-modulated antigen tetanus. Responses specific to this trial include responses to a positive control antigen expressed in LNCaP tumor cells, the influenza M1 antigen, and to prostate tumor antigens such as prostate-specific membrane antigen (PSMA) and prostatic acid phosphatase (PAP). Clinical responses will include monitoring of adverse events, PSA levels, and measurable disease.

## Kind of clinical trial: Project Phase

Phase I/II prospective randomized, single-blinded, placebo-controlled trial with 2 arms describing the safety, immunogenicity and activity of vaccination with DC/LNCaP, DC/LNCaP-M1, and DC/KLH.

## Estimated number of patients

24 patients with pathologically confirmed prostate cancer, progressive disease, who have no active CNS metastases or vital organ compromise, ages 18 years of age and older.

Group 1. 12 patients, receiving DC/LNCaP, DC/LNCaP-M1, DC/KLH, and either vaccine vehicle only (5% DMSO andin normal saline ).

Group 2. 12 patients, receiving placebo (saline or vaccine vehicle only (5% DMSO in normal saline )) for 8 weeks followed by DC/LNCaP, DC/LNCaP-M1, DC/KLH and either saline or vaccine vehicle only (5% DMSOin normal saline ).

## Risks of particular severity based on animal/prior human studies

None (see anticipated risks, below).

# Investigator’s Brochure.

Not applicable (PI is sponsor-investigator).

# Protocols

## Study Protocol #1

### Face Sheet

**Title of Study**

A Phase I/II Study of Autologous Dendritic Cells Pulsed with Apoptotic Tumor Cells (DC/LNCaP) Administered Subcutaneously to Prostate Cancer Patients.

**Product to be evaluated**

DC/LNCaP vaccine in prostate cancer. DC/LNCaP-M1, DC/KLH, and either saline or vaccine vechile alone will be administered as vaccination controls.

Principal Investigator and Sponsor (Study IND Holder)

Robert B. Darnell, MD, PhD

Head, Laboratory of Molecular Neuro-Oncology

The Rockefeller University

Box 226

1230 York Avenue

New York, NY 10021-6399

Tel: 212-327-7460

Fax: 212-327-7147

darnelr@rockvax.rockefeller.edu

**Project Assurance Number**:

**IND number**:

BB-IND 10710

**Clinical Site**: The Rockefeller University Hospital, 1230 York Avenue, New York, NY 10021

**Clinical Study Coordinator**: Mayu Frank, MS, ANP

**GCRC Clinical Research Officer**: Rhonda Kost, MD

**Clinical Investigators:**

Robert B. Darnell, M.D., Ph.D.

Rockefeller University

1230 York Avenue

New York, NY 10021

212-327-7460

Howard Scher, MD

Memorial Sloan Kettering Cancer Center

1275 York Avenue

New York, NY 10021

646-422-4323

Mayu Frank, MS, ANP

Rockefeller University

1230 York Avenue

New York, NY 10021

212-327-7443

Susan Slovin, MD PhD

Memorial Sloan Kettering Cancer Center

1275 York Avenue

New York, NY 10021

646-422-4323

Laboratory Investigators:

Julia Kaufman, Ph.D.

Rockefeller University

1230 York Avenue

New York, NY 10021

212-327-7453

Olga Diaz-Pulido

Rockefeller University

1230 York Avenue

New York, NY 10021

212-327-7453

**IRB name/address**

Institutional Review Board

Rockefeller University Hospital

1230 York Avenue

New York, N.Y. 10021-6399

### Précis

The objectives of this protocol are to assess the acute toxicity and immunogenicity of a new autologous dendritic cell-based vaccine. This vaccine consists of autologous DCs generated ex vivo that are cultured with apoptotic-killed tumor cells. The tumor cells are the prostate cancer cell line LNCaP. The vaccine is termed DC/LNCaP.

The population to be enrolled will be prostate cancer patients who have failed conventional therapy and who have rising PSA levels, indicative of a poor prognosis. The design of the trial will be a Phase I/II prospective randomized, single blind, placebo controlled trial. There will be two arms to the trial. In the first arm, patients will be injected subcutaneously with the DC/LNCaP vaccine as well as the DC/LNCaP-M1 and DC/KLH vaccine controls. In the second arm, patients will initially be given subcutaneous injections of placebo, and then crossed over to receive the DC/LNCaP vaccine and DC/LNCaP-M1 and DC/KLH vaccine controls over the same time schedule as patients in the first arm.

Assessment of immunogenicity will be aided by the use of a positive control antigen introduced into LNCaP cells. This antigen will be the influenza matrix antigen, M1, which has been stably introduced into LNCaP tumor cells, and will be delivered as a second vaccine, termed DC/LNCaP-M1.

Toxicity will be assessed by objective clinical measures. These will include both local and systemic clinical evaluations. Activity will be assessed by measuring PSA levels and immune response to antigen, including delayed type hypersensitivity (DTH) reactions to control antigen (KLH) and to the experimental vaccine.

### Background

It is estimated that about 8 million men in the United States harbor foci of cancer in their prostate (Scardino et al., 1992). Prostate cancer is the second leading cause of cancer-related death in men, killing ~37,000 individuals per year (Landis et al., 1999). Prostate cancer patients with metastatic disease; and newly diagnosed patients with rising levels of tumor markers post- surgical debulking or radiotherapy, present difficult clinical problems and special opportunities for exploring new methods of treatment. For example, a patient with a high-grade tumor whose levels of prostate tumor markers, e.g. prostate-specific antigen (PSA), rise within 2 years (presumably a manifestation of microscopic disease), has a nearly 50% chance of developing clinically evident metastatic disease within 3 years (Pound et al., 1999; Scher, 1999; Slovin and Scher, 1999). No therapy, including hormone ablation or chemotherapy has been shown in randomized trials to improve survival in these groups (Scher, 2003). In the large study of Pound and colleagues, all patients who developed metastatic disease died from their prostate cancer, with a mean survival of slightly less than 5 years (Pound et al., 1999).

These observations emphasize that new treatment approaches are desperately needed for high risk prostate cancer patients. At the same time, these patients offer several advantages for evaluating the efficacy of new treatments. Many high risk prostate cancer patients are typically highly functional, particularly the subset of patients whose only risk is high blood levels of PSA. This clinical marker also offers the possibility of a simple and reliable means of monitoring treatment efficacy. Additionally, since chemotherapy is of no proven benefit for these patients, it is not a confounding factor in evaluating new treatments. Note this latter point is critical in the testing of immunotherapy protocols.

Our work has demonstrated that effective tumor immunity in humans is associated with, and likely mediated at least in part by tumor antigen-specific killer T cells (Albert et al., 1998a; Darnell, 1999; Darnell and Posner, 2003). This observation is consistent with analogous findings in animal models of tumor immunity (Markiewicz and Gajewski, 1999). Although significant progress is being made toward understanding ways in which to stimulate antigen-specific T cell responses to specific antigens in humans, such studies have yet to demonstrate the development of functional tumor-specific T cell responses.

Bhardwaj and colleagues have done perhaps the most careful analysis of the therapeutic use of autologous human dendritic cells (DCs), an antigen presenting cell known to be responsible for the activation of T cell *in vivo*. Autologous *ex vivo* generated DCs pulsed with the MHC class I restricted influenza peptide, as well as cells pulsed with neo-antigens (e.g. KLH), have been shown to (i) be safe for human use and (ii) be efficacious in their ability to stimulate and boost influenza-specific CD8^+^ T cell responses as demonstrated in cytotoxicity assays (Dhodapkar et al., 2000; Dhodapkar et al., 1999). Reassuringly, these studies have shown a remarkable safety profile, with no significant acute toxicity reported in over 100 DC injections (Dhodapkar and Bhardwaj, 2000).

However, experiments using peptide-pulsed DCs have yielded somewhat low CTL responses, as compared, for example, to *in vivo* levels of influenza-specific CD8^+^ T cells detectable following viral infection. Moreover, MHC I-restricted 9 amino acid peptide epitopes pulsed onto the MHC class I molecules of DCs provides no obvious means to activate CD4^+^ helper cells, cells critical for memory responses and possibly important for the generation of effective anti-tumor immune responses. Thus, immune stimulation by peptide pulsed DCs may be a sub-optimal means of generating effective *in vivo* cellular immunity.

Apoptotic material derived from dying cells have been shown to be a potent means of delivering antigen to DCs and subsequently triggering antigen-specific T cell responses (Albert et al., 1998b; Albert et al., 1998c; Inaba et al., 1998). The apoptotic material is effectively phagocytosed by DCs, at least in part through a receptor mediated process involving the α_v_β_5_ integrin (Albert et al., 1998b). The internalized material is then processed within DCs for the generation of both MHC I and MHC II peptide epitopes, in turn, permitting the activation of both CD4^+^ and CD8^+^ T cells. In addition to the priming of the CD4^+^ T cell compartment, apoptotic cells offer several advantages over peptides when delivering antigens: (i) multiple epitopes are delivered simultaneously to the DC, as opposed to the single epitope employed in peptide-based therapies; (ii) the utilization of all MHC alleles, in comparison to the one MHC allele targeted by peptide-based therapies; and (iii) the high efficiency of antigen delivery via this methodology—10,000 to 50,000 times more efficient than the utilization of peptide (Inaba et al., 1998; Li et al., 2001). By employing this physiologically relevant pathway for the delivery of antigen to DCs we may effectively trigger the development of potent prostate cancer-specific immune responses. The differences between the strategy proposed and typical DC-based immunization strategies are outlined in Table 1.

Table 1. Comparison of immunization strategies

| **DC/LNCaP** | **Peptide-pulsed DC** |
| --- | --- |
| Multiple cellular proteins are processed by apoptotic machinery | Individual protein chosen by investigator used |
| Multiple epitopes from each protein are processed for MHC presentation | Single epitope from a single protein used |
| Proteins processed for MHC I and MHC II presentation, allowing CD8 and CD4 T cell stimulation | Peptides designed for MHC I |
| All (typically 6) different MHC I alleles used to present peptides from each protein | Peptides designed for one (typically HLA A*0201) MHC I allele |
| High efficiency (estimate 10,000-50,000 greater than peptide) protein presentation | Low efficiency |
| Activation of T cells with physiologic T cell receptor (TCR)/MHC I affninity | Activation of T cells with low affinity TCR |

### Objectives

The primary objective in this Phase 1 study will be to ascertain the safety of the DC/LNCaP vaccine. More precisely, the study will monitor the safety of employing *ex vivo* generated autologous dendritic cells (DCs) pulsed with apoptotic prostate cancer cells as a therapeutic in prostate cancer patients.

An additional objective of this study is to evaluate the immunogenicity of autologous DCs pulsed with apoptotic prostate cancer cells. The primary immunogen in this study, the DC/LNCaP vaccine, does not harbor known antigens to which predictable immune responses can be studied and definitively be attributed to the vaccination. For this reason, patients will be immunized with the DC/LNCaP-M1 vaccine in separate subcutaneous inoculations at the time of immunization with the DC/LNCaP vaccine. This positive control immunogen harbors an antigen, the influenza matrix antigen, to which most patients have been exposed, and to which baseline and post-immunization responses can be monitored. In this way, the study is designed to yield definitive information regarding immunogenicity of autologous DCs presenting antigen derived from apoptotic tumor cells. Because of the theoretical concern that the overexpressed M1 epitopes might displace endogenous prostate tumor epitpoes, the vaccination will be split between “pure” DC/LNCaP cells and the DC/LNCaP-M1 vaccine control.

Secondary objectives in this study will be the evaluation of the clinical course of immunized patients. This will include both subjective analysis of patient symptoms, and objective analysis of clinical tests performed before and after vaccination. Any analysis of clinical or immunologic data obtained from patients who do receive conventional therapy after vaccination will need to be evaluated with this variable taken into consideration.

### Subjects

24 patients with pathologically confirmed prostate cancer, progressive disease, who have no active CNS metastases or vital organ compromise, ages 18 years of age and older, available for at least 41 weeks of follow-up from the time of screening.

Arm 1. 12 patients, receiving DC/LNCaP vaccine and the DC/LNCaP-M1 and DC/KLH immunizations over 8 weeks.

Arm 2. 12 patients in the placebo Arm for 8 weeks followed by DC/LNCAP vaccine and the DC/LNCaP-M1 and DC/KLH immunizations over 8 weeks.

#### Proposed enrollment

24 patients will be enrolled. Implementation of enrollment is contingent upon approval of the protocol and consent for by the Rockefeller University institutional review board (IRB). Candidates identified for study entry will have the details of the study carefully discussed, prior to asking the individual to read and sign the consent form. Patients choosing to enter the study will be randomized according to a randomization schedule provided by the Biostatistician.

#### Inclusion/exclusion criteria

Screening to determine eligibility (with the exception of HLA haplotyping) will be completed within 45 days of study entry.

##### --Disease Characteristics--

1. Histologically confirmed prostate carcinoma

2. Progressive, disease required, i.e.: elevated PSA documented to be rising on 3 occasions, either despite castrate testosterone levels (below 50 ng/dl), or after definitive local therapy (prostatectomy or radiation).

| Extent of disease | Min # of values | Interval |
| --- | --- | --- |
| Rising PSA within 2 years of prostatectomy | 3 | >2 weeks |
| Rising PSA post-radiation^*^ | 3 | >2 weeks |
| Rising PSA despite castrate testosterone | 3 | >2 weeks |

^*^Baseline is defined as the first of three rising values confirmed after definitive radiation treatment.

3. No active CNS metastases

##### --Prior/Concurrent Therapy--

1. Recovered from toxicity of any prior therapy

2. Biologic therapy:

No prior autologous or allogeneic tumor vaccines

No concurrent other immunotherapy

3. Chemotherapy:

At least 4 weeks since chemotherapy

Not previously treated with more than 2 chemotherapy regimens

No concurrent chemotherapy

4. Endocrine evaluation/therapy:

3 rising PSA values at least 2 weeks apart

At least 2 weeks since concurrent corticosteroids (other than for replacement therapy for adrenal insufficiency)

Medical hormonal therapy to maintain castrate testosterone levels permitted

5. Radiotherapy:

At least 4 weeks since radiotherapy

No concurrent radiotherapy

6. Surgery:

Prior surgery allowed

##### --Patient Characteristics--

1. Age: 18 and over, able to give written informed consent. Individuals unable to provide informed consent must have consent provided by the legal guardian, or person designated by the subject to give consent on his behalf.

2. Performance status: Karnofsky 70-100%

3. Life expectancy: At least 1 year

4. Hematopoietic: obtained twice, once within 45 days prior to study entry, and again within 72 hours of study entry.

WBC greater than 3,800

Absolute neutrophils greater than 1,500

Absolute lymphocytes greater than 500

Platelets greater than 120,000

Hb at least 10 g/dl

5. Hepatic:

Bilirubin less than 2.0 mg/dl OR

SGOT less than 2 x ULN

6. Renal:

Creatinine no greater than 2.0 mg/dl OR

Creatinine clearance at least 40 ml/min

7. Rheumatologic:

ANA no greater than upper limit of normal, or ANA abnormal in absence of clinical signs of autoimmunity.

Rheumatoid factor (RF) no greater than upper limit of normal, or RF abnormal in absence of clinical signs of autoimmunity.

Anti-ds DNA no greater than upper limit of normal, or anti ds DNA abnormal in absence of clinical signs of autoimmunity.

1. Immunologic:

Influenza serology (assessment made at time of screening).

Assessment of DTH response to a tandard anergy panel (to include candida, trichophyton and tetanus) or to a Multitest CMI (a disposable kit for DTH testing with standardized preloaded antigens).

1. Cardiovascular:

No NYHA class III/IV status

No active angina, clinically significant cardiac arrythmia, recent (6 months) myocardial infarction

1. Pulmonary:

No severe debilitating pulmonary disease

1. Endocrine:

TSH, T3, and T4 no greater than upper limit of normal

1. Radiographic:

Baseline bone scan

Baseline CT or MRI of abdomen and pelvis

1. Other:

No active infection requiring antibiotics

No active pain requiring chronic opioid analgesics.

Not HIV, hepatitis B or hepatitis C virus positive; anti-HIV, HbsAg and Hep C antibody negative

No history of hypersensitivity to vaccine components

No serious uncontrolled medical illness

No currently active second malignancy other than non-melanoma skin cancer (note: a patient is NOT considered to have currently active malignancy if they have completed therapy and are now considered by their physician to be at less than 30% risk for relapse)

No history of total lymph node irradiation

No history of vasculitis, including but not limited to systemic necrotizing vasculitides (polyarteritis nodosa group), hypersensitivity vasculitis, Wegener’s granulomatosis.

No history of autoimmune disease.

No use of hydroxyurea within 45 days of study entry

No receipt of immune modulators or suppressors within 30 days prior to study entry, including but not limited to interferons and thalidomide. No active requirement for corticosteroids; prior use is acceptable.

No psychiatric illness or social condition that, in the opinion of the investigator, would interfere with adherence to study requirements.

No alcohol or drug use or dependence that, in the opinion of the investigator, would interfere with adherence to study requirements.

##### Gender and Minority Inclusion

|  | American Indian or Alaskan Native | Asian or Pacific Islander | Black, not of Hispanic Origin | Hispanic | White, not of Hispanic Origin |
| --- | --- | --- | --- | --- | --- |
| Female | 0 | 0 | 0 | 0 | 0 |
| Male | 2 | 1 | 3 | 1 | 17 |
| Unknown | 0 | 0 | 0 | 0 | 0 |
| Total | 2 | 1 | 3 | 1 | 17 |

##### Participation of Children

Prostate cancer is not a disease of children. The age-specific mortality rate for invasive prostate cancer in the years 1992-1996 was 0/100,000 for individuals under age 34 (NCI SEER Cancer Statistics Review, 1973-1996). However, since rare instances may occur in young adults, the study will be open to eligible individuals age 18 or older.

### Vaccines

**DC/LNCAP:** Autologous dendritic cells that have been co-cultured with apoptotic LNCAP cells.

**DC/LNCAP-M1:** Autologous dendritic cells that have been co-cultured with apoptotic LNCAP-M1 cells that are stably transfected with a plasmid cDNA expressing the influenza matrix antigen.

**DC/KLH:** Autologous dendritic cells that have been exposed to KLH.

**Lot numbers**: 02-A1 to 03-A24

**Dose concentration**: 2-10 x 10^6^ DC/LNCaP per injectate, 2-10 x 10^6^ DC/LNCaP-M1 or 2-10 x 10^6^ DC/KLH per injectate. Cells are resuspended in 0.5 ml 5% DMSO and 25% autologous serum in sterile saline for injection.

**Route of administration**: Cells will be injected into the subcutaneous tissue of the upper arm or thigh with a tuberculin syringe.

**Placebo**: either saline or vaccine vehicle only (5%DMSO in sterile saline.

### Study Design

Phase I/II prospective randomized, single-blinded, placebo-controlled trial with 2 arms describing the toxicity and activity of immunization with DC-LNCaP. Study participants in Arm 2, but not the investigator, study personnel, or those study participants in Arm 1 will be blinded with respect to up-front treatment (vaccine or placebo). At week 8, all patients will be made aware of which treatment arm they are in.

Arm 1. Subcutaneous injection of *ex vivo* generated autologous dendritic cells pulsed with apoptotic LNCaP cells, pulsed with apoptotic LNCaP-M1 cells (control apoptotic cells) and pulsed with KLH (control antigen). In addition, subcutaneous injection of either saline or vaccine vehicle only (5% DMSO in normal saline ).

Arm 2. Subcutaneous injection of either saline or vaccine vehicle only (5% DMSO in normal saline ), followed by cross-over to Arm 1 design.

#### Dosing Plan

All prostate cancer patients will undergo two leukapheresis procedures, one prior to vaccination and a second 6 weeks after the final vaccination. Those in Arm 2 will undergo a third leukaphereesis, prior to the placebo phase. Their cells will be cryopreserved in aliquots and will serve as a source of (prevaccination or postvaccination) cells throughout the study and will reduce the number and volume of blood draws.

The study is divided into 2 Arms of 12 patients each.

Arm 1 will have autologous DCs generated from pre-vaccination leukapheresis cells. Laboratory manipulation of the cells will be done according to Good Laboratory Practice guidelines. Cells will be co-cultured with apoptotic LNCaP or LNCaP-M1 tumor cells for an average of 36 hours (range 30-48 hours) prior to immunization. Vaccinations will occur on weeks 1, 3, 5 and 7. On weeks 1 and 3 patients will also be immunized with mature DCs exposed to KLH. For each subject, the maximal number of available DC will be re-injected with an anticipated range of 2 x 10^6^ to 10 x 10^6^ cells, depending on cell availability, in 0.5 ml volume. Patients will also be injected with either saline or vaccine vehicle only (5% DMSOin normal saline) at weeks 1, 3, 5 and7.

Arm 2 will have placebo (either saline or vaccine vehicle only (5% DMSOin normal saline )) injected on week 1, 3, 5 and 7, and will then switch over to DC/LNCaP and DC/LNCaP-M1 immunization on weeks 9, 11, 13 and 15. On weeks 9 and 11 patients will also be immunized with mature DCs exposed to KLH. Patients will also be injected with either saline or vaccine vehicle only (5% DMSO in normal saline ) at weeks 9, 11, 13 and15.

#### Duration

Immunizations will be given over the course of 7 weeks. All patients will be followed to Week 17 (Arm 1) or Week 25 (Arm 2). The longest duration of follow-up will be blood draws 29 (Arm 1) or 37 (Arm 2) weeks after study entry in selected patients with either clinical or immunological responses or adverse events. Patients who have completed at least 13 weeks (Arm 1) or 21 weeks (Arm 2) of the study will be considered to have completed the study, although all patients will be encouraged to remain in the study for the full 17 (Arm 1) or 25 (Arm 2) weeks.

#### Schedule of Events

##### Clinical and Laboratory Evaluations.

The Schedule of Events listed below (Table 2) includes all study evaluations. The definitions in the following section define the evaluations, provide timelines, and include special considerations or instructions.

##### Definitions for Schedule of Events

###### Screening. Screening will generally be completed within 45 days prior to study entry.

Medical History: The Principal Investigator or his designee will perform a thorough medical history.

Complete Physical Examination: The Principal Investigator or his designee will perform a complete physical exam at the indicated times.

Tests listed below will be conducted on samples collected and analyzed by standard procedures.

If any laboratory result is outside the reference range and is considered to be clinically relevant by the Investigator, the test will be repeated at appropriate time intervals until it returns to baseline or becomes a clinically insignificant finding.

Complete Blood Count: including differential white blood cell count, absolute neutrophil count, hemoglobin and platelet count.

Hematologic Tests: PT/PTT.

Blood Chemistries: Including total electrolytes (Na, K, Cl and bicarbonate, glucose, serum BUN and creatinine, alkaline phosphatase, and albumin.

Liver Function Tests: AST, ALT and total bilirubin.

Urinalysis: Including microscopic analysis.

Viral screens: HCV antibodies, Hepatitis B surface antigen, HIV antibodies, Influenza serology.

Rheumatologic screens: ANA, RF, dsDNA.

Thyroid screens: TSH, T3, T4.

HLA haplotype: complete HLA haplotype is obtained as part of the patient database, but is not used as a clinical screening parameter. Results of HLA haplotyping do not need to be available prior to leukapheresis at week 0.

Immunologic: DTH testing to standard anergy panel, including candida, tryichophyton and tetanus.

EKG

Radiologic Tests will be performed at Memorial Sloan-Kettering Cancer Center (preferably) or New York Weill Medical College. All tests will be read by a professional radiologist and compared with prior studies whenever these are available.

Chest X-Ray: Standard PA and lateral chest X-Ray, to be completed during screening, and, for follow-up imaging during the study, within 10 days of the dates indicated in the Schedule of Events.

Bone Scan: To be completed during screening, and, for follow-up imaging during the study, within 10 days of the dates indicated in the Schedule of Events.

CT/MRI: For indicated patients, standard imaging of the abdomen and pelvis, without and with contrast, unless contrast is medically contraindicated. Either CT or MRI scans are acceptable. For follow-up imaging during the study, within 10 days of the dates indicated in the Schedule of Events.

Table 2. Schedule of Events.

Figure 1. Study flow chart.

###### On-study evaluations

Entry (Week 0)

Entry day is defined as the day of leukapheresis, which is performed 8-9 days (preferably 8 days) prior to the first immunization (which occurs on “Week 1”).

Directed History and Physical: The Principal Investigator or his designee will perform a directed history and physical, with the aim of assessing any new or unresolved signs and symptoms experienced since the last visit.

At baseline (week 0) all signs and symptoms will be recorded on the CRF. For post-baseline assessments (week 1 and subsequently), all Grade 2 or higher signs/symptoms will be recorded on the CRF. Please see Table 3 for grading of local reactions to vaccines.

Any signs or symptoms that lead to a change in treatment, regardless of grade, must be recorded on the CRFs.

Leukapheresis. All subjects in Arm 1 undergo leukapheresis at entry (“Week 0”) and all subjects in Arm 2 undergo leukapheresis at Week 0 and 8. Post treatment, all patients will undergo another leukapheresis. In the event we cannot use a leukapheresate, such as due to a poor cell yield or a contamination, the procedure may be repeated. Alternatively, in such an event, previously frozen down monocytes if available may be used to prepare the vaccine.

This procedure will be performed at The Rockefeller University GCRC.

Note: Hematology evaluations are required within 1 week prior to the leukapheresis.

100 cc of whole blood will be drawn for making autologous plasma on Week 0. White blood cells from this blood may be stored and used for additional assessment of baseline immunologic function (e.g. allogeneic mixed lymphocyte reactions, allo-MLR).

Week 1, 3, 5, 7 (Arm 1) and week 1, 3, 5, 7, 9, 11, 13, 15 (Arm 2)

History and physical, and indicated blood draws must occur prior to study vaccination.

Vaccination:

Arm 1 (up-front vaccine):

Weeks 1, 3, 5 and 7 autologous DCs co-cultured with apoptotic irradiated LNCaP cells are given SQ in single injections of 500 μl. At each of these times, autologous DCs co-cultured with apoptotic irradiated LNCaP-M1 cells, as well as either saline or vaccine vehicle alone (5% DMSOin normal saline) are given SQ in single injections of 500 μl. On weeks 1 and 3, autologous DCs exposed to KLH are also given SQ. The SQ injections may be given +/- 3 days from the scheduled visit, with the exception that the first injection, which will be given on day 8 (preferably) or day 9 after the patient completes leukapheresis.

Arm 2 (placebo followed by vaccine):

Weeks 1, 3, 5 and 7 Either saline or vaccine vehicle alone (5% DMSO in sterile saline) is given SQ in single injections of 500 μl. At week 9, 11, 13, and 15 autologous DCs co-cultured with apoptotic irradiated LNCaP cells are given SQ in single injections of 500 μl. At each of these latter times, autologous DCs co-cultured with apoptotic irradiated LNCaP-M1 cells, as well as either saline or vaccine vehicle alone (5% DMSOin normal saline ) are given SQ in single injections of 500 μl. On weeks 9 and 11, autologous DCs exposed to KLH are also given SQ. The SQ injections may be given +/- 3 days from the scheduled visit, with the exception that the first injection, which will be given on day 8 (preferably) or day 9 after the patient completes leukapheresis.

Administration of Vaccine

The DC/LNCaP vaccine is given approximately 6-8 cm from the axilla in the inner aspect of the upper arm. The DC/LNCaP-M1 vaccine is given approximately 6-8 cm from the axilla in the inner aspect of the patients’ contralateral upper arm. The administration site is prepared with betadine and alcohol swabs. Injection is given with a 25-gauge needle by superficial SQ injection (not in the fatty part). DCs exposed to KLH and either saline or vaccine vehicle alone may be given in the anteromedial thigh approximately 12-24 cm distal to the inguinal ligament.

Immunologic Studies

DTH: DTH will be performed at the Rockefeller University GCRC, concurrent with each vaccination and at 2 weeks, 10 weeks and in some patients, at 22 weeks post last vaccination. DTH reactions will be read at the Rockefeller University GCRC between 40-48 hours after implantation. Results will be recorded on the CRF.

Lymphoproliferative responses, ELISPOT and CTL: Assays will be performed at the Rockefeller University.

### Outcome Parameters

#### STUDY ENDPOINTS

##### Toxicity—assessment of safety and tolerability

Toxicity will be assessed by evaluation of Grade 3 or higher adverse events, which upon review are considered to be at least possibly related to study vaccine.

All adverse events noted during the study will be tabulated by body system/organ class. All subjects will be observed in the Rockefeller University Hospital following immunizations for evidence of immediate and subacute local and systemic reactions. They will be instructed to watch for local and systemic reactions for 7 days post-immunization and will be evaluated by an investigator if significant symptoms are reported. This includes evaluation of redness, swelling and pain at the injection site and in draining lymph nodes. Objective measures will include measurement of the area of redness or swelling, and subjective measures will include self-assessment of pain on a scale of 1-10.

Systemic measures will include evaluation of fever, malaise and nausea.

Routine measurements of hematology, serum chemistry, and urinalysis laboratory tests will be performed as outlined in the Schedule of Events.

##### Activity

Activity of vaccination will be measure by monitoring both clinical and immunologic parameters. The primary clinical parameter of activity will be PSA measurement. Secondary clinical parameters include monitoring of measurable disease, assessed by chest X-Ray, bone scan and CT scan/MRI scans.

PSA will be measured in the MSKCC laboratory and serially monitored starting at the screening visit, Day 0, Week 3, 4, 5, 7, 9, 13, and 17, (and possibly 29) for participants in Arm 1 and on screening visit, Week 3, 4, 5, 6, 7, 9, 11, 12, 13, 15, 17, 21, and 25, (and possibly 37) for participants in Arm 2.

Complete response (CR): PSA < 4.0 ng/ml for 3 successive evaluations at least two weeks apart.

Partial Response (PR): Decrease in PSA value by > 50% (without normalization) for three successive evaluations at least two weeks apart.

Stabilization (SD): Patients who do not meet the criteria for PR or PD for at least 90 days on study (post vaccine; week 1 (Arm 1) or week 10 (Arm 2)).

Progression (PD): Three consecutive increase in PSA from baseline or nadir, each measurement at least two weeks apart.

Time to PSA Progression: The first PSA rise after the nadir PSA that is confirmed by two consecutive PSA at least two weeks or more apart.

Measurable disease

Patients will be evaluated at baseline, and at 5 weeks and 17 weeks after completion of therapy. Response and progression of measurable disease will be evaluated by bone scan and soft-tissue CT scan, using criteria proposed by the Response Evaluation Criteria in Solid Tumors (RECIST) Committee (Therasse et al., 2000). Changes in only the largest diameter (uni-dimensional measurement) of the tumor lesions are used in the RECIST criteria. Note: Lesions are either measurable or non-measurable using the criteria provided below.

Measurable disease: Defined as lesions that can be accurately measured in at least one dimension (longest diameter to be recorded) as >20 mm with conventional techniques (PET, CT, MRI, x-ray) or as >10 mm with spiral CT scan.

Non-measurable disease: All other lesions, including small lesions, are considered non-measurable disease. Bone lesions, leptomeningeal disease, ascites, plueral/pericardial effusions, lymphangitic disease, inflammatory disease are all non-measurable.

Target lesions: All measurable lesions up to a maximum of five per organ and 10 in total representative of all involved organs should be identified as target lesions and recorded and measured at baseline. Target lesions should be selected on the basis of their size (lesions with the longest diameter) and their suitability for accurate repeated measurements (either by imaging techniques or clinically). A sum of the longest diameter (LD) for all target lesions will be calculated and reported as the baseline sum LD, which will serve as a reference by which to characterize tumor response.

Non-target lesions: All other lesions should be identified as non-target lesions and should also be recorded at baseline. Non-target lesions include measurable lesions that exceed the maximum numbers per organ or total of all involved organs as well as non-measurable lesions. Measurements of these lesions are not required, but the presence or absence of each should be noted throughout follow-up.

Guidelines for evaluation of measurable disease: All measurements should be taken and recorded in metric notation using a ruler or calipers. All baseline evaluations should be performed as closely as possible to the beginning of treatment and never more than 4 weeks before the beginning of the treatment. Tumor lesions that are situated in a previously irradiated area are not considered measurable. The same method of assessment and the same technique should be used to characterize each identified and reported lesion at baseline and during follow-up. Imaging-based evaluation is preferred to evaluation by clinical examination when both methods have been used to assess the antitumor effect of a treatment.

Radionuclide bone scan

Subjectivity in interpreting serial changes in radionuclide bone scan is well recognized. Thus the primary outcome will be whether the scan is stable or improved, vs. worse or progression. Changes in intensity will not be used as an outcome measure.

Stable or improved: Requires that no new lesions appear, or that new pain has not developed in an area that was previously visualized.

Progression (Non-response): New areas of focal uptake.

Time to Progression: Time to progression will be defined as the time from the first day of treatment with vaccine until the date PD or death is first reported. Patients who die without a reported prior progression will be considered to have progressed on the day of their death.

Chest x-ray

Lesions on chest x-ray are acceptable as measurable lesions when they are clearly defined and surrounded by aerated lung. However, CT is preferable.

CT and MRI

These techniques should be performed with cuts of 10mm or less in slice thickness contiguously. Spiral CT should be performed using a 5 mm contiguous reconstruction algorithm. This applies to tumors of the chest, abdomen, and pelvis. Head and neck tumors and those of extremities usually require specific protocols.

##### Immunogenicity

The primary parameter of immunogenicity will be measurement of DTH responses. Secondary parameters include of assessment of T cell responses by measurement of lymphocyte proliferation, ELISPOT and CTL assays.

###### Delayed type hypersensitivity (DTH) responses

At screening, patients will be assessed for DTH response to positive control antigens (a standard “anergy panel” to include candida, trichopyton and tetanus). Patients with no positive response to the anergy panel are not eligible for trial entry. Responses to one of the anergy panel antigens will be subsequently used as a positive control non-modulated antigen. Each antigen will be placed subcutaneously using a tuberculin syringe to inject a volume of 100 μl.

DTH responses during the study will be to the control non-modulated antigen and to various vaccine components, specifically the control immunizing antigen KLH, and to aliquots of killed LNCaP cells and LNCaP-M1 cells, to DC/LNCaP cells and DC/LNCaP-M1 cells, and as negative controls, to DCs which have not been peptide pulsed and to saline. DTH response will be assessed by measuring the region of induration surrounding the injection site of each antigen. Each antigen will be placed according to a stereotyped grid, and induration will be read between 40 and 48 hours after injection.

###### Lymphocyte proliferation assay (LPA)

Lymphocyte proliferative response to specific vaccine component antigens (which include the influenza M1 antigen) and to the control antigen KLH.

Lymphoproliferative responses of subject PBMC or purified CD4^+^ T cells (MACS columns; Miltenyi Biotech) to antigens and mitogens are measured by incubating 1 x 10^5^ cells per well in 96-well U-bottom plates with serial antigen concentrations of protein, recall antigen (tetanus toxoid, KLH) and in separate plates with serial dilutions of PHA, PWM and ConA. After 3 days of incubation with the mitogens and 6 days with the antigens, cells are pulsed with 1 μCi/well of (^3^H)-thymidine for 6hr and then harvested using the Tomtec (EG&G Wallac, Gaithersburg, MD) and counted in a 1450 microbeta trilux (EG&G Wallac, Gaithersburg, MD). The data are expressed as an LSI (Lymphocyte stimulation Index = (PBMC cpm + antigen or mitogen)/(PBMC cpm + medium)) to define antigen specificity. Individuals are arbitrarily designated as responders or non-responders if their LSI is greater than or equal to 5.

###### ELISPOT analysis

ELISPOT assays for gamma-interferon production from isolated CD4^+^ and CD8^+^ T lymphocytes or peripheral blood monocytes in response to vaccine antigen stimulation (or other relevant antigens as they become available) in an overnight culture system.

Ninety-six well plates (Milititer Millipore, Bedford, MA) are coated overnight at 4^o^C with 5μg/ml anti-IFN-γ Mab (Mabtech, Stockholm, Sweden). The antibody coated plates are washed 4 times with PBS and blocked with RPMI containing 5% pooled human serum (PHS) for 1h at 37 ^o^C.

T cells are added to plates containing anti-IFN-γ antibodies, and antigen-presenting APCs are added for 12-24 hours. Alternatively, PBMCs or CD8^+^ T cells purified by magnetic bead antibody cell sorting (MACS columns; Miltenyi Biotech) can be added to the plates. Antigen-presenting APCs may be DCs pulsed with peptides (e.g. HLA restricted influenza or PSMA1/2 peptides) or may be cross-presenting antigen (e.g. apoptotic EL4 cells stably transfected with influenza matrix antigen). 24-48 hours after co-culture of T cells and antigen-presenting APCs, a second biotinylated anti-IFN-γ antibody is added, followed by an avidin-peroxidase complex. Development with an HRP substrate enumerates IFN-γ producing cells as demonstrated by “spots” and reported as spot forming cells (SFC). A positive response is defined as >100 SFC per 1x10^6^ T cells. If ELISPOT responses are negative, we will perform “recall” ELISPOT assays, in which purified T cells are stimulated with antigen-loaded DCs for 7 days and then tested for antigen specificity in ELISPOT assays. Only spots with a fuzzy border and a brown color are counted. Responses are considered positive if a minimum of 10 SFCs are counted after the control has been subtracted.

###### Cytotoxic T-lymphocyte (CTL) responses

CTL response against vaccine component antigens (or other antigens as they become available) presented on HLA-restricted target cells in standard bulk chromium release assays.

Cytolytic assays will measure ^51^Chromium release from target cells to measure circulating effector or memory CTL activity. Freshly derived circulating CD8+ T cells are assayed for their effector CTL activity by monitoring their ability to lyse ^51^Cr labeled target cells that have been pulsed with HLA-restricted peptides, or which have been virally transduced to express specific antigens via the endogenous or “classical” MHC I antigen presentation pathway. Expansion of central memory T cell responses are assayed by recalling T cells in vitro with antigen-loaded DCs and then assessing CTL activity on labeled targets. We will employ standard means of assaying for HLA-restricted CTLs in patients that are HLA- using ^51^Cr loaded T2 cells pulsed with defined peptides as target cells. HLA A*0201 restricted peptides have been defined for the influenza M1 antigen (GILGFVTL, amino acids 58-66, (Gotch et al., 1987)). A positive response is defined as >15% cytotoxicity at an effector:target (E:T) ratio of 20:1. Percent cytotoxicity is determined by using the average values of triplicates from experimental wells (E) compared with average values of spontaneous (S) and total (T) release as follows: % cytotoxicity = ((E - S)/(T - S)) x 100.

Percent specific killing will be reported as a measure of lysis of antigen loaded target cells as compared to antigen negative target cells, defined as the median counts per minute (CPM) of gamma particles produced by ^51^Cr in the supernatants taken from the respective target cell populations.

#### Interim and Final Analysis

Preliminary safety analysis will be performed after each immunization is given. A final analysis will occur after all visits are completed. Final analysis will include an assessment of the relationship between the total number of dendritic cells administered and PSA levels as well as total number of dendritic cells administered and immunologic responses.

### Monitoring and follow-up.

Patients will be monitored intensively at the Rockefeller University Hospital following each vaccination. Patients will be monitored directly for one hour after each vaccination. Patients may stay longer or may be admitted as necessary or at the discretion of the PI. Antihistamines and epinephrine will be available for treatment, as appropriate, of any allergic reactions. The local injection site and draining lymph nodes will be monitored for inflammation and swelling. Patients receiving placebo will not receive DTH tests (other than saline), so as not to tolerize them to subsequent vaccination.

Possible local reactions that should be recorded include erythema and induration (measured using a flexible ruler and pain/tenderness, or warmth at the injection site). Local or systemic reactions will be noted on the case report forms.

In the event of a local or systemic reaction, patients will be monitored until the reaction has resolved. All grade 3 or more severe reactions will require immediate notification of a study physician. Monitoring will include direct supervision by nursing and physician staff, monitoring of vital signs and physical examination as needed, and other appropriate medical measures as deemed necessary.

During the week after each immunization, the subject will be examined or contacted by phone by the investigator or his designee to obtain local and systemic reaction data and to determine the subject’s clinical status. Instructions to subjects leaving the Rockefeller University GCRC post-vaccination will include directions and telephone numbers to use in the event of any unusual or severe sign or symptoms. Subjects reporting such symptoms should be seen in the Rockefeller University GCRC at the time of maximal symptoms, and will be followed up clinically until resolution of symptoms.

Interim outpatient visits will include history and physical examinations as well as monitoring of clinical laboratory studies, as detailed in the schedule of events. Between outpatient visits, for the first 7 days post immunization, a self-assessment questionnaire will be completed by the patient with instructions to notify the study team by phone if any systemic symptoms or unusual or severe symptoms are experienced.

Longer term follow up will be performed at graded intervals following vaccination (weeks 9, 13, 17 and possibly 29 in Arm 1, and weeks 17, 21, 25 and possibly 37 in Arm 2).

#### Monitoring Adverse Events

Subjects having adverse events will be monitored with relevant clinical assessments and laboratory tests as determined by the Investigator. All adverse events must be followed to satisfactory resolution or stabilization of the event(s).

Any actions taken and follow-up results must be recorded on the appropriate Case Report Form, as well as in the subject’s source documentation. Follow-up laboratory results should be filed with the subject’s source documentation.

For all adverse events that require the subject to be discontinued from the study, relevant clinical assessments and laboratory tests will be repeated on at least a weekly basis, until final resolution or stabilization of the event(s).

##### Adverse Events Data Collection

At each study visit, the investigator or designee will collect all information on changes in signs and/or symptoms from baseline, in the form of a targeted history and physical. Laboratory tests will also be obtained to assess safety as described in “Schedule of Events,” and will be reviewed as results become available. For protocol related problems, patients will either be provided with or referred for any medical follow up as necessary. Patients will be referred to their primary care provider for non-protocol related health problems. This information along with the outcome will be recorded as part of the medical record.

The GCRC nursing staff will also assess each patient during inpatient hospitalization. Prior to the start of the study, the GCRC nursing staff will be given a thorough inservice on the study, so that they are well aware of the expected adverse events. Independently of the study team, these nurses will assess for and record any signs or symptoms deviating from baseline in the medical record. They will also report such occurrences directly to the investigator or designee.

##### Adverse Event Reporting

As described above, adverse events data is collected and documented by both the investigator/designee and the GCRC nursing staff. The study nurse practitioner will keep a log of all such adverse events recorded in the medial record, with a start and stop date. Each adverse event will be graded using NCI’s Common Toxicity Criteria, version 3.0, and the relationship of the adverse event to the study treatment will be described per NCI’s guidelines as definite, probable, possible, unlikely, or unrelated. Any medical follow up and outcome of the adverse event is also logged. All expected adverse events are listed in the Post Injection Assessment Form (attached). Any adverse event that is not listed in this form will be considered to be an unexpected adverse event. The nurse practitioner will log each adverse event as either expected or unexpected.

During all inpatient visits, the GCRC nursing staff reports all adverse events using a secure electronic reporting system maintained by the Systems Administrator (Alex Pechtchanski) of The Rockefeller University Hospital. This report, generated during the work shift in which the adverse event occurred or was reported, describes the adverse event, any interventions given, and the outcome when known. This report is electronically sent to the Medical Director, the Principle Investigator (PI), the GCRC Clinical Research Officer, the Hospitalist, and the GCRC Pharmacist. The Hospitalist determines each adverse event to be serious or not serious. These reports are then signed and dated by the Hospitalist and retained in the Office of Clinical Research. This process of reporting and reviewing adverse events is independent of the investigator reporting and reviewing.

All adverse events will be tabulated from the AE log and reported to the IRB and FDA in the annual progress report. Information to be reported includes all adverse events, the CTC grade assigned, and attribution to the investigational agent. Total enrollment, demographics of enrollment, protocol violations, current status of subjects (completed, ongoing, dropout), reasons for attrition, the number of subjects reaching primary endpoints, and any available research data will also be reported.

#### Serious Adverse Events

All serious adverse events (defined below), whether or not deemed drug-related or expected, must be reported by the Principal Investigator or designee to the IRB within 24 hours (one working day) by telephone, and to the GCRC Clinical Research Officer. Written reports must follow as soon as possible, which include full descriptions of the event and any sequelae. The PI will report any unexpected and serious adverse event within 7 calendar days by phone or fax to the FDA, and follow within 15 calendar days by written form to the FDA. Any actions taken by the FDA or IRB will be reported by the PI to the NCI.

These stipulations includes serious adverse events that occur any time after the inclusion of the subject in the study (defined as the time when the informed consent is signed), up to 30 days after the subject completes or is discontinued from the study. The subject is considered completed after the completion of the last visit, either as documented in the Study Flow Chart of the protocol. Discontinuation is the date a subject and/or Investigator determines that the subject can no longer comply with the requirements for any further study visits or evaluations (eg the subject is prematurely discontinued from the study; see Stopping rules).

A serious adverse event in this study is defined as any event that:

Is fatal,

Is life-threatening (defined as the patient being at immediate risk of death from the AE as it occurred),

Is significantly or permanently disabling,

Requires in-patient hospitalization, or prolongs hospitalization.

Important medical events that may not fit these criteria may also be considered a serious adverse drug experience when, based upon appropriate medical judgment, they may jeopardize the patient and may require medical or surgical intervention to prevent one of the outcomes listed in this definition.

In addition to reporting serious and unexpected adverse events to the FDA, the PI will also submit in a written report any finding from tests in laboratory animals that suggests a significant risk for human subjects including reports of mutagenicity, teratogenecity, or carcinogenicity within 15 calendar days of the PI’s initial receipt of the information.

Any severe adverse reactions (CTC Grade 3) will also be reported by the PI to the GCRC Clinical Research Officer within 48 hours. Medical intervention will be initiated as appropriate and referred for further care if necessary. The PI will not authorize further administration of the vaccine to other patients until consultation with the GCRC Clinical Research Officer and Rockefeller University’s IRB.

### Benefits

This is a research study, and the potential benefit of this vaccine as a cancer treatment is entirely unknown. However, the patient will be involved in the testing of a new therapeutic approach. The patient will receive intensive exposure to medical staff and care. Patients enrolled in this study will be offered the opportunity to enroll in subsequent studies to further evaluate toxicity and efficacy of this vaccination.

### Risks and complications

The most likely risk from participation in this study is pain and swelling at the site of inoculation. There are also risks associated with blood drawing and leukapheresis that all study subjects may anticipate. The risks associated with leukapheresis and immunization may be considered separately.

#### Risks associated with Leukapheresis

Leukapheresis is a means of drawing blood meant to save the subject multiple blood draws and loss of RBCs. Leukapheresis is a procedure very much like blood donation for the subject. A needle is placed in the arm, and blood is collected as in the blood bank. Unlike blood donation, however, the patient’s own red blood cells, platelets and plasma are returned to the patient, which helps to prevent anemia. Only the patient’s white blood cells are kept for clinical study. Less than 1% of an individual’s white blood cells are normally in circulation, and ready pools are available in the spleen and lymph nodes to replenish donated white blood cells, so that the cells removed at leukapheresis are replenished by the patient almost immediately.

The risks involve the use of an invasive needle, which may lead to bruising of the arms where the needle is placed, and which may lead to the introduction of bacteria and infection. There is a minor risk of bleeding from anticoagulants given during the procedure; this anticoagulant (citrate) is broken down by the body within one minute, any bleeding will be stopped by terminating the leukapheresis. The citrate may cause mild, transient hypocalcemia, which may be associated with symptoms of tingling or muscle cramping. Rarely, patients may faint (suffer hypotension due to a vasovagal reaction) during leukapheresis; for this reason patients lie down during the procedure and are accompanied at all times by medical staff. Patients who are taking ACE inhibitors will have their dose held until after the leukapheresis procedure is completed. Patients sometimes feel chilled during leukapheresis; if they do, they will be given blankets to keep warm.

Needles used for leukapheresis may be placed in the veins in the arm. In patients for which insufficient venous access is available in the arms, needles may instead be placed in larger veins, typically the femoral vein located at the top of the leg. These needles entail the same kinds of risks as needles placed in the arm (bleeding, infection), but has a risk of producing large hematomas in the leg, and may be less comfortable than needles placed in the arm.

#### Risks associated with immunization

There is a risk of local vaccine injection site reactions. It is likely that temporary pain, redness, and swelling will develop at the injection sites. If needed, ice packs or oral acetaminophen will be used to treat this.

There is slight risk of infection from the injection of DCs. To minimize this risk, the vaccine will be manufactured in a laboratory governed by Good Laboratory Practice principles and in biosafety cabinets. There are no known alternatives to the protocol described to generate dendritic cells loaded with multiple prostate tumor cell antigens. Although the experiments are novel in design, the risks of an infusion of autologous cells pulsed with apoptotic LNCaP cells should be minimal. This includes an allergic reaction and the induction of autoimmunity. An allergic reaction is unlikely given that the patient’s own cells are being given back. Care is taken in the protocol to prepare the patients’ DCs in autologous plasma to reduce the possibility of an allergic reaction. In the event of an allergic reaction, the patient will be treated with appropriate medications (epinephrine, benadryl) if indicated and watched closely during the hospital stay.

DCs pulsed with individual peptides have been given to over 100 normal volunteers and cancer patients at the Rockefeller University GCRC and at outside institutions, with no evidence of adverse immune responses other than minor pain and redness at the sites of injection.

An immune reaction to LNCaP cells has been attempted in several tumor immunotherapy protocols done by other groups (although not apoptotic LNCaP cells pulsed onto DCs), as a desired means of inducing tumor immunity. The antigens present in LNCaP cells that are substantially different from the patient’s own set of proteins may elicit an immune response. This may lead to a reaction against LNCaP cells, and a reaction to other cells expressing such proteins. It is believed in this protocol that the other cells present in the patient’s body that are expressing such LNCaP antigens are the patient’s prostate cancer cells. Whether a reaction against other cells in the patient’s body may occur is unknown, and constitutes the basis of the risk for developing autoimmunity. The investigators believe this risk to be low. T cells capable of reacting with normal self antigens are believed to be deleted at birth or tolerized (inert) during the course of the patient’s lifetime. Given these considerations and the experience of other physicians with dendritic cell vaccines, the risk of autoimmunity, while unmeasurable, is considered to be small.

While we do not anticipate significant toxicity, the experiments will proceed with the following cautions. To watch for evidence of adverse reactions, including allergic or autoimmune reactions, patients will be closely monitored during the first hour after each vaccination. The local injection site and draining lymph nodes will be monitored for inflammation and swelling. A battery of tests for adverse reactions will be carried out at frequent intervals during the trial.

#### Balance of risk:benefit.

The balance of the risks versus benefits involved in participation are judged by the investigators to be clearly in favor of the benefits derived from participation. The risks are judged overall to be moderate, but are not anticipated to include serious adverse reactions. This is a research study, and the potential benefit of this vaccine as a cancer treatment is entirely unknown. The benefits include the intensive health care monitoring patients will receive, as well as the potential that if this protocol proves safe, patients will be offered the opportunity to enroll in subsequent studies to further evaluate toxicity and efficacy of this vaccination.

### Withdrawal from study

Patients are entitled to withdraw from this study at any time. The investigators may withdraw patients from this study who develop serious adverse reactions to the vaccine. All patients who withdraw from the study will be asked to continue to be followed as specified in the protocol.

### Stopping rules

#### Criteria for Subject Study Discontinuation

- Development of an exclusionary condition as outlined in section (6)i.e.2, prior to or during vaccination, with the following exceptions:
  - a course of palliative radiation treatment during the study
- platelet counts that drop > 50% from baseline.
  - hemoglobin that drops sufficiently to require transfusion.
  - if the patient develops an infection requiring treatment as an inpatient.
- Failure by the subject to attend three consecutive clinical study visits.
- Request by the subject to withdraw.
- Clinical reasons judged by the study physician to be life threatening, even if not addressed in the toxicity management of the protocol.
- Completion of week 17 or in some patients, week 29 in Arm 1.
- Completion of week 25 or in some patients week 37 in Arm 2.
- Any dose limiting toxicity. A dose limiting toxicity is defined as:
  1. any grade 2 or greater allergic toxicity or asymptomatic bronchospasm or generalized urticiaria
  2. any grade 3 or greater toxicity (hematologic or non-hematologic)
  3. any grade 2 or greater autoimmune reactions

If a subject discontinues prior to completion of the study, the reason for and date of the discontinuation will be recorded. The date of the last dose of study vaccine will also be recorded. Subjects who discontinue prior to study completion may be replaced at the discretion of the Investigator.

#### Vaccine Toxicity

The Common toxicity Criteria (CTC) version 3.0 (http://ctep.info.nih.gov) grading system will be used for grading the severity of adverse events. For adverse events not covered by the CTC grading system, the following definitions will be used:

Table 3. Grading and Attribution of Local Reactions to Vaccine

| Grade | Symptoms | Signs |
| --- | --- | --- |
| 0 = None | No pain or tenderness (VAS 0) | N/A |
| 1 = Mild | Minimal pain or tenderness, no limitation of arm use (VAS 1-3), mild warmth | Erythema or induration less than 15 x 15 cm (225 cm^2^) |
| 2 = Moderate | Notable pain or tenderness, some limitation of use of arm (VAS 4-7), moderate warmth | Erythema, induration or edema > 15 x 15 cm (225 cm^2^) |
| 3 = Severe | Extreme pain or tenderness, complete limitation of use of arm (VAS 8-10), hot | Ulceration, superinfection or phlebitis |
| 4 = Potentially life threatening | N/A | Necrosis of the skin |

The Investigator will also asses the relationship of any adverse event to the use of study drug, based on available information, using the following NCI-based attribution guidelines:

| Attribution | Description |
| --- | --- |
| Unrelated | The adverse event is CLEARLY NOT RELATED to the investigational agent. |
| Unlikely | The adverse event is DOUBTFULLY RELATED to the investigational agent. |
| Possible | The adverse event MAY BE RELATED to the investigational agent. |
| Probable | The adverse event is LIKELY RELATED to the investigational agent. |
| Definite | The adverse event is CLEARLY RELATED to the investigational agent. |

Note: it is anticipated that study patients will experience transient tenderness, redness or swelling at the local site of vaccine injection or DTH testing. Subjects should be told prior to administration that these anticipated reactions might occur. The subjects will be closely monitored following each immunization.

Local reactions (Grade 1-2)

Local reactions of mild (Grade 1) or moderate (Grade 2) severity will generally resolve without treatment. If needed, they may be managed with local application of ice packs or oral acetaminophen.

Local reactions (Grade 3-4)

Severe (Grade 3) or potentially life threatening (Grade 4) local reactions, the Rockefeller University GCRC Clinical Research Officer (Dr. Rhonda Kost) must be notified within 48 hours. For grade 4 local reactions, definitive medical and/or surgical intervention should be undertaken as appropriate. Following Grade 3 or Grade 4 local reaction, further vaccines should not be given to other patients prior to consultation with the Rockefeller University GCRC Clinical Research Officer (Dr. Rhonda Kost), and by the PI in conjunction with the Rockefeller Universities Institutional Review Board (IRB).

Systemic reactions

Systemic reactions will be graded according to the CTC version 3.0 (http://ctep.info.nih.gov).

All nonlocal Grade 3 or 4 reactions (for example, elevated temperature following immunization) thought definitely, possibly or probably related to vaccination, will be reported within 48 hours to the Rockefeller University GCRC Clinical Research Officer (Dr. Rhonda Kost). Further vaccines should not be given to other patients prior to consultation with the Rockefeller University GCRC Clinical Research Officer (Dr. Rhonda Kost), and by the PI in conjunction with the Rockefeller Universities Institutional Review Board (IRB).

The study will be stopped if 1 of 3, 2 of 6, 3 of 9, or 4 of 12 subjects experience dose limiting toxicity as defined under 6.i.m. Stopping rules, 1) Criteria for Subject Study Discontinuation. This applies to subjects in both Arm 1 and 2 while he is receiving the investigational product.

If a patient is injected with a DC-vaccine prior to knowing that a contaminant was present (e.g. prior to a 14-day culture becoming positive), the patient will be immediately contacted and undergo a complete history and physical examination. Blood cultures and appropriate laboratory tests will be drawn.

### Data analysis

This study is a phase I/II, single-blinded study, involving the use of an investigational new drug, conducted at The Rockefeller University Hospital. As such, this plan is written in accordance with FDA regulations on IND safety reports and annual reports (title 21 part 312.32 & 33), and ICH’s Good Clinical Practice Guidelines. This plan also meets NIH and NCI policies (NIH Policy for Data and Safety Monitoring, Further Guidance on a Data and Safety Monitoring for Phase I and II Trials, NCI Essential Elements of a Data Safety and Monitoring Plan for Clinical Trials Funded by the NCI), in the event the study receives such funding.

#### Analysis of safety data.

##### Quantitation of toxicity data.

Local reactions are measured in quantitative measures of pain, induration and redness. This data will be tabulated and monitored in order to be able to report on the quantitative extent of discomfort resulting from vaccination.

Systemic reactions are quantitated on a severity scale of grade I-IV. All reactions will be recorded and the percentage of reactions of each grade tabulated relative to the number of patient inoculations to determine frequency of adverse systemic reactions.

##### Quantitation of clinical immunologic data.

Specific measures of autoimmune response will be tabulated and monitored in order to be able to report on the quantitative extent of elevation of autoimmune immunologic parameters. These include serial measurements of ANA, dsDNA, and RF. Severity of reactions will be graded on a scale of I-IV, and results tabulated relative to the number of patient inoculations to determine frequency of elevation of autoimmune immunologic parameters.

#### Analysis of immunogenicity data.

Immunogenicity data will be tabulated and quantitated for each of the quantitative assays planned. These include proliferation assays, ELISPOT assays, and CTL assays. For example, we will correlate positive or negative responses (proliferation index > 5, ELISPOT T cell responses > 100 SFC/10^6^ T cells, and CTL responses with % specific killing > 15%) with positive or negative responses to our positive control antigens (influenza M1) and, where available, to the prostate antigens PSMA and PAP.

For ELISPOT, CTL response, LPA and DTH, the primary analysis will be based on the change from baseline. Changes in baseline will be summarized by the median and 25^th^ and 75^th^ percentiles, and arms compared with the Wilcoxon rank sum test. Additionally, the longitudinal responses will be examined using generalized estimating equation analyses (to incorporate all the responses on a subject).

#### Biostatistical analysis.

The primary specific aim is to compare the clinical status during the first part of the study (before the cross-over) between the placebo and the vaccine group. As defined in CFR, Phase-I/II studies should address both acute toxicity and activity/efficacy. Accordingly, this comparison will be based on two primary outcomes:

(1) activity: relative change in PSA.

We do not use the term “efficacy” here, because we recognize that change in PSA is not a validated “surrogate” for clinical benefit. The primary manifestations of disease progression in prostate cancer are rising PSA values, new lesions on bone scan, new symptoms of disease, and, less frequently, an increase in measurable tumor mass. Several groups have shown the prognostic significance of post-therapy declines in PSA, leading to an NCI consensus group proposal of a set of guidelines for the use of post-therapy PSA that can be used in phase II trials to identify agents for further testing (Fazzari et al., 2000).

(2) acute toxicity: incidence and severity of an adverse events.

Although the variables will be combined to address the overall clinical status, the power calculations below address each of these aspects independently.

Size of relevant differences: For activity, we would like to have sufficient power to detect a 50% decline in PSA in the treatment group vs. no change in the placebo group. For toxicity, we would like to have sufficient power to detect a 50% incidence of severe adverse events (acute toxicity) vs. a 5% incidence.

Power vs. level: If there should be acute toxicity, we do not want to overlook this (false negative result). A similar argument holds for activity: as long as the treatment with apoptotic LNCaP-DCs is well tolerated, we would rather go on with the next trial, even if we would later find out that apoptotic LNCaP-DCs are not active (false positive result), than to risk delaying the development of an effective treatment (false negative result). Thus, we want to guarantee 90% power for the above differences at the 10% level. If we should see lower acute toxicity in the vaccine group (unlikely) or higher activity in the placebo group (also unlikely), our decision would be the same as if we would see no difference: we would reconsider the use of this vaccination in the treatment of advanced prostate cancer. Given this one-sided decision strategy we will further increase power by using one-sided tests.

Statistical model: As the form of the relationship between changes in PSA and clinical success is unknown, we will use a Wilcoxon-Mann-Whitney Ranktest to compare activity. For SAEs, the exact Fisher test will be used.

Sample size: To detect any difference with 90% power at the 10% level (one-sided) the WMW test’s chi-square distribution must have a non-centrality parameter of 8.2 under the alternative. By our inclusion criteria, all patients will have had rising PSA levels prior to study entry. Thus, for the power calculation, we assume that PSA continues to remain elevated or to increase over the 9 week study period for at least two-thirds of the patients in the placebo group. If the vaccination should be active, PSA should decrease in two-thirds of the patients in the vaccine group. If we assume that even the largest decline expected in the placebo group does not exceed 50%, then this corresponds to the consensus agreement that a 50% decline should be considered relevant (Bubley et al., 1999; Fazzari et al., 2000). Conservative scenarios of relevant differences in PSA responses between the vaccine and placebo group, yield sample sizes between 9 and 13. From a Bayes'ian perspective, none of these alternative hypotheses is unlikely. Thus, with a sample size of 12 per group we can expect at least 90% power for detecting relevant activity at the 10% level for changes in PSA.

Regarding toxicity, this sample size also guarantees a 90% power to detect an increase in the cumulative incidence of SAEs from 5% in the placebo group to 50% in the vaccine group at the 10% level (or a 80% power at the 5% level). A 40% cumulative incidence could be detected with 80% power at the 10% level (or a 90% power at the 20% level). If no SAEs are seen in the placebo group, three SAEs in the vaccine group yield a p-value of .109 (one-sided, exact). With one SAE in the placebo group, four and five severe adverse events in the vaccine group yield a p-value of .158 and .077, respectively.

This suggests the following conservative stopping rule for the study: After the third SAE in the vaccine group, the study is stopped if no SAEs have occurred in the placebo group until this time. After the fourth SAE in the vaccine group, the study is stopped unless more than one SAE has already occurred in the placebo group.

Benefit: For the final analysis, PSA markers and adverse event indicators will be combined to assess the overall benefit of the vaccination. We will compute marginal likelihood scores (Susser et al., 1998) based on the relative change in PSA and the highest adverse event category observed in each patient. SAEs will "overwrite" any benefit of the vaccination, while other adverse events will augment the benefit. Thus, if large declines in PSA are observed in patients with more adverse events, the overall benefit of the vaccination will be downgraded. On the other hand, if adverse events are predominantly observed among patients with increase in PSA, this strategy increases power.

### Medical care

Medical care at the Rockefeller University Hospital will be provided as needed to monitor and respond to immune reactions and toxicity to vaccination. Medical care will be provided both during outpatient visits, and, when necessary, as inpatients. In the event of serious adverse reactions necessitating intensive medical care, patients will be transferred to Memorial Sloan-Kettering Cancer Center, or to New York Hospital Presbyterian Medical Center, both institutions where the Principle Investigator is on the adjunct clinical staff. Protocol related care will be given free of charge.

Private physicians referring patients will be responsible for management of unrelated medical illness and management of tumor symptoms and disease progression during the study and at completion of the formal period of study.

### Data accuracy and protocol compliance.

Enrollment forms, case report forms vaccine reaction diaries and data collection forms are included in the Appendices.

#### Records to be kept

Enrollment forms will be filled in at the time of patient entry. Case report forms (CRFs) willl be provided for each subject. Subjects will not be identified by name on CRFs. Subjects will be identified by Patient Code Number (PC#) and Study Identification Number (SID) assigned at the time of patient entry.

#### Record monitoring and availability

Records will be monitored by the Rockefeller GCRC Clinical Research Office or an appropriate monitor not affiliated with the Principle Investigator’s laboratory. Monitoring will include review of the individual subject records, including consent forms, CRFs, supporting data, laboratory specimen records, and medical records, to ensure compliance with the protocol, and accuracy and completeness of records.

The investigator will make study documents and pertinent hospital or clinic records readily available for inspection by the local IRB, the Rockefeller GCRC Clinical Research Officer, the Food and Drug Administration (FDA), and the Office for Human Research Protections (OHRP).

#### Data Accuracy and Protocol Compliance

##### Clinical Data:

Upon completion of screening activities, and prior to enrollment, the PI or his designee will verify that all inclusion criteria are met and no exclusion criteria are met. The PI or his designee will complete the Entry Criteria page of the CRFs to document verification, and upon enrollment, dictate an enrollment note. Clinical data will be collected primarily by the study team but also by the GCRC nursing staff. The data will be collected per protocol and documented in the medical record. The study team will use the CRF as a guide to ensure the completeness of the data collected at each visit. When the GCRC nursing staff is to collect the data, in addition to the inservice at the start of the study, they will be given specific instructions in the “Doctor’s Orders.”

The study coordinator will enter this data from the source document into case report forms (CRFs). Any protocol violations will also be logged as part of the CRFs. Upon completing the screening, a patient identification code will be assigned to that patient. This code will be used to identify each subsequent CRF completed and on all laboratory specimens. The study coordinator will then transcribe all CRF data into the electronic CRF database. This electronic CRF is protected by 2 different passwords for confidentiality. Computers with access to this electronic CRF are kept in locked offices. Those with authority to enter data and/or change data are listed in the Delegation of Authority Log. Only those with such authority have password access to the electronic CRFs. The Laboratory Administrator is the only other person who has access to it as the database administrator. This electronic CRF database maintains an audit trail. Any new or changed data to be added will have its own field and will not destroy the existing data. Each entry will be automatically stamped with a date, time, and the data enterer’s identification. This database is protected from power failures, as some of the computers with access to this database are laptops with battery power. The database is protected from machine and network failures as it is backed up daily onto two different hard drives.

##### Laboratory Data:

All laboratory vaccines, specimens, and data will be labeled with the patient ID number only. No other identifying information will be used. Standard Operating Procedures must be followed for all procedures in this GLP laboratory. Laboratory data will be collected into bound laboratory notebooks. This data will then be transcribed into an electronic database. This database is password protected with access restricted to the PI and his designees.

##### Ongoing Data Monitoring

External monitoring by an independent auditor of the cleanroom and cleanroom procedures will be done on an annual basis in accordance with Good Tissue Practice guidelines and records will be kept.

Ongoing data and safety monitoring will be done by the monitoring team. The team consists of the PI (Dr. Robert B. Darnell) and members of the study team (Mayu Frank, Dr. Julia Kaufman, and Dr. Athanasios Dousmanis), in consultation with the Clinical Research Officer of The Rockefeller University Hospital GCRC Advisory Committee. This team will meet monthly to review the following:

###### Study and participant progression:

Review the number of participants screened and enrolled

Review the number of participants progressing and their status

Review the number of attritions and their reasons

Review any general concerns or recurrent problems

###### AE log:

Review the AE log for completeness (start and stop dates, expected or unexpected, grading, attribution, follow-up and outcome)

Review for appropriate reporting to the IRB, FDA, NCI

###### PI report on new information:

Review any new or additional information that have impact on the safety of the participants or the ethics of the study, such as new scientific or therapeutic developments.

###### Clinical data review:

Medical record charts of up to three patients per month will be randomly pulled. The medical record will be reviewed for protocol compliance (including meeting entry criteria, documentation of the consenting process, complete and timely assessments and obtaining of laboratory tests) accurate transcription into the AE log, accurate transcription into the database (CRFs), and for timeliness in this transcription.

###### Laboratory data review:

Laboratory data for up to three patients per month will be randomly selected for review.

Records of this monthly review will be kept by the nurse practitioner. If it is found that there is a potential or actual deficiency in patient safety or data integrity/validity in any way, the PI (in consultation with the GCRC Clinical Research Officer when appropriate) will recommend actions, and will conduct the monitoring sessions more frequently until resolved.

### Consent form

A signed, dated, IRB-approved consent form is required prior to participation in this study.

The consent form is included in Appendix VI.

## Investigator Data

See form FDA 1572

## Facilities Data

See form FDA 1572

## Institutional Review Board Data

See form FDA 1572

# Chemistry, manufacture & control

## Chemistry, Manufacturing and Control Data

### Drug Name

**DC/LNCaP:** Autologous dendritic cells which have been co-cultured with apoptotic killed LNCaP cells.

**DC/LNCaP-M1:** Autologous dendritic cells, which have been co-cultured with apoptotic, killed LNCaP-M1 cells, which are LNCaP cells stably transfected with a plasmid cDNA expressing the influenza matrix antigen.

**DC/KLH:** Autologous dendritic cells which have been exposed to KLH.

### Quality/Ingredients

Each vaccines used in this study, DC/LNCaP, DC/LNCaP-M1, and DC/KLH will be resuspended in 5% DMSO and 25% autologous serum in sterile saline for subcutaneous administration.

Autologous DCs will be tracked throughout the study in such a way as to ensure identity. Each donor will have their own unique shelf area within the incubator, and be physically isolated from other donors’ cells. A unique patient identification number (PIN) will be used to track each donors cells and ensure the reinjection of autologous DCs.

### Biologic characteristics

A biologic vaccine consisting of autolgous DCs that have been co-cultured with irradiated, apoptotic, LNCaP tumor cells. A control vaccine will consist of autolgous DCs that have been exposed to KLH. Either saline or the vaccine vehicle alone will also be given as a control.

#### Name/address of manufacturer

**DC/LNCaP**

Rockefeller University

1230 York Avenue

New York, New York, 10021

**DC/LNCaP-M1**

Rockefeller University

1230 York Avenue

New York, New York, 10021

**DC/KLH**

Rockefeller University

1230 York Avenue

New York, New York, 10021

## Flow diagram—Vaccine Preparation

Day 6

Mycoplasma Testing

Microbiology Testing after co-culture**

Vaccine Testing

*Results not available until after injection.

**Use 24-48 hr read for release.

Figure 2. Flow diagram for Vaccine production and testing.

## Drug substance

### LNCaP Tumor Cells

#### Validation of LNCaP tumor cell identity

The LNCaP prostate tumor cell line was obtained from the ATCC (clone FCG, catalog No. CRL-1740). These cells were maintained throughout growth in our laboratory in defined media, with careful documentation since their arrival in the laboratory (see below, (7)v.a).

Identification of LNCaP cells was confirmed by two independent assays. First, LNCaP cells are reportedly HLA A*0201, which we confirmed with our LNCaP cells by FACS analysis (Fig. 3). Second, LNCaP cells are reported to synthesize PSMA and PSA, which we confirmed in our LNCaP cells by Western blot (Fig 3 and data not shown). These studies also confirmed that LNCaP-M1 cells express the influenza M1 antigen (Fig 3B).

Figure 3. Validation of LNCaP cell identity.

#### Validation of LNCaP apoptotic death

##### Validation of LNCaP apoptotic cell death protocol.

We analyzed the response of the LNCaP prostate tumor cell line to the apoptotic-inducing stimuli. We quantify early apoptotic death using CaspaTag (Intergen Corp), a newly developed cell-permeable reagent, detects active caspase molecules, and is specific for apoptotic cells. Necrosis is assessed by either staining with propidium iodide, 7AAD, or trypan blue, which identifies cells with permeable membranes. Half maximal apoptotic death is achieved in LNCaP cells following 60-120 mJ/cm^2^/sec UVB exposure. Secondary necrotic death does not occur until 24 hours post UVB exposure. Based on these studies and our functional data reported below, 120 mJ/cm^2^/sec UVB exposure will be used for our study.

##### Validation of LNCaP apoptotic cell capture by DCs.

To demonstrate that apoptotic LNCaP are effectively phagocytosed by human DCs, we performed a FACS-based phagocytosis assay established in the laboratory. LNCaP cells were first labeled with a red colored vital dye, PKH26 (Sigma Immunochemicals) and subsequently exposed to UV irradiation to trigger apoptotic death. After co-culture with green-labeled DCs, achieved using PKH67, two-color FACS analysis was performed to determine the kinetics of uptake of apoptotic material by DCs. DCs efficiently phagocytosed apoptotic LNCaP cells with >50% double positive cells by 4 hours.

##### Validation that LNCaP apoptotic cells are not viable.

We assessed whether the apoptotic LNCaP cells prepared in (1) above are replication incompetent both *in vitro* and *in vivo*. In tissue culture, we found that LNCaP cells show no evidence of replication, as assessed by incorporation of ^3^H-thymidine, 36 hours after irradiated with UV-B (120mJ/cm^2^/sec). *In vivo*, we have assessed the ability of prostate cancer cells to grow in SCID and *nude* mice. These toxicity data are summarized in Fig. 2, and illustrate that we are not able to detect any live cells in an *in vivo* assay in 5 x 10^7^ irradiated cells.

Figure 4. Lack of detectable viability in apoptotic LNCaP cells.

#### LNCaP Master Cell Bank

##### Source of Master Cell Bank LNCaP cells

LNCaP cells obtained from the ATCC and subsequently grown and validated in our laboratory as described in (b) below will be used to produce a LNCaP Master Cell Bank. Cells for the Master Cell Bank will be grown in our GLP facility. These cells will be grown in Aim V media supplemented with 1% USA certified BSE-free fetal calf serum. Between 100-200 lots will be generated and frozen in Aim V media with 1% FCS supplemented with 5% DMSO.

##### Quality of LNCaP Master Cell Bank

LNCaP cells from the Master Cell Bank will be characterized to confirm cell identity and for adventitious agents.

###### Cell identity.

Cell identity will be confirmed using Western blot and FACS analysis to demonstrate HLA haplotype (HLA A*0201), and expression of the prostate antigens PSMA and PSA. Assays will be performed as described in Figure 3. In addition, isoenzyme testing will be performed.

###### Testing for adventitious agents.

Testing will be done in an FDA certified commercial laboratory (BioReliance). Testing will include the following:

routine bacterial, mycoplasma and fungal cultures, and endotoxin (LAL) testing.

in vivo and cell culture inoculation test for adventitous viruses, including specialized tests for the presence of HIV 1/2, HTLV-I and II, CMV, EBV, B-19, Hepatitis B and Hepatitis C., and Retrovirus by PERT assay. These tests will use FDA-approved methods.

### LNCaP-M1 Master Cell Bank

#### Source of Master Cell Bank LNCaP-M1 cells.

An important aspect of our clinical protocol is the use of positive controls to monitor the efficacy of our immunization. As per pre-IND discussions with the FDA, we have constructed a plasmid construct harboring the following elements. The backbone of the plasmid contains a ColE1 origin of replication, a dual Kanamycin resistance gene (for selection in prokaryotes) and Neomycin resistance gene (for selection in eukaryotes), as well as a multi-cloning site for insertion of the M1 gene. A CMV promoter drives the cDNA encoding the influenza matrix antigen. This plasmid construct was validated by DNA sequencing, transfected into LNCaP cells by electroporation, and G418-resistent colonies expanded. We confirmed that these LNCAP-M1 cells make M1 antigen, PSMA and PSA (Fig. 3B and data not shown).

A LNCaP-M1 Master Cell Bank will be grown as described for the LNCaP Master Cell Bank.

#### Quality of LNCaP-M1 Master Cell Bank

Testing of the quality of LNCaP-M1 cells and testing for adventitious agents (by BioReliance) will be performed as described above for preparation of the LNCaP Master Cell Bank.

### Autologous DCs

#### Source of Autologous DCs

Autologous DCs are prepared from patients peripheral blood monocytes. Leukapheresis is used to obtain sufficient cell numbers.

For entry into the clinical study, DCs generated from each prostate cancer patient must meet morphologic, phenotypic and functional criteria (allo-MLR). We have developed assays to assess the quality of DCs obtained from prostate cancer patients. DCs are grown according to a well accepted adherence method (Thurner et al., 1999).

#### Method for Preparation of Immature DCs

Peripheral blood mononuclear cells (PBMCs) are obtained by leukapheresis as detailed in Appendix III. Cells are adhered to endotoxin free tissue-culture dishes (Falcon cat # 3003), thus allowing for the fractionation of monocytes (adherent cells) and a T-cell rich population (non-adherent cells). Immature DCs are differentiated from monocyte precursors over the course of six days using GM-CSF (180 ng/ml) and IL-4 (1000-2000 units/ml) as described by Lanzavecchia (Sallusto and Lanzavecchia, 1994).

#### Method for Preparation of Mature DCs

Immature DCs are matured (either in the presence or absence of apoptotic cells) for 36-72 hours with 20 μM PGE2 and 180 ng/ml TNF-α (Rieser et al., 1997), as detailed in Appendix III.

#### Quality of Autologous DCs

Cells are monitored for the expression of DC markers by FACS analysis, and for function by allo-MLR.

Figure 5 demonstrates the highly pure populations of immature and mature dendritic cell populations with a typical stellate morphology and phenotype from prostate cancer patients. DCs assessed by FACS lacked of expression of CD14 on day 6, and induced surface expression of CD83 following 2-3 days of maturation. In addition, the mature DCs demonstrated high levels of both HLA-DR and the costimulatory molecule CD86 (B7-2).

Figure 5. Quality assessment of DCs grown from a prostate cancer patient.

### Acceptable limits and analytical methods to assure identity, strength, quality, purity

#### Identity and Quality

##### LNCaP tumor cell identity and quality

LNCaP cells are assessed by FACS and Western blot analysis. LNCaP cells will have the following characteristics:

- 100% HLA haplotype, assessed by FACS assay (using antibody Cat # HB-82, ATCC).
- Expression of PSMA positive, assessed by Western blot analysis (using antibody clone 7E11-C5.3, Cytogen).
- Expression of PSA positive, assessed by Western blot analysis (using antibody Cat # V1054, Biomeda).

LNCaP-M1 cells is assessed by FACS and Western blot analysis. LNCaP-M1 cells will have the following characteristics:

- 100% HLA haplotype, assessed by FACS assay (using antibody Cat # HB-82, ATCC).
- Expression of PSMA positive, assessed by Western blot analysis (using antibody clone 7E11-C5.3, Cytogen).
- Expression of PSA positive, assessed by Western blot analysis (using antibody Cat # V1054, Biomeda).
- Expression of influenza M1 antigen positive, assessed by Western blot analysis (using antibody Cat # HB64, ATCC).

##### DC identity and quality

DC purity will be assessed by FACS analysis. DCs will have the following characteristics:

>70% CD83+, assessed by FACS assay (using antibody Cat # 36935X, Pharmingen).

>90% HLA-DR high, assessed by FACS assay (using antibody Cat # 347367, Beckton Dickinson).

<15% CD14+, assessed by FACS assay (using antibody Cat # 347493, Beckton Dickinson).

#### Strength

DC strength is assessed by allo-MLR. DCs will have the following characteristics:

DCs present at a ratio of 100 DCs to one T cell will be able to stimulate 2 x 10^5^ allogeneic T cells to incorporate > 10^5^ CPM of ^3^H-thymidine after 24 hours of co-culture. To measure incorporation of ^3^H-thymidine, T cells are harvested onto a filter card using a 96-well harvester (TomTec) and analyzed for beta-emission using a Wallac Trilux Luminometer. A stimulation index > 10 will indicates active DCs. Stimulation Index (SI) is greater than 10. SI = (DC + T cell)/(T cell + media).

#### Purity

##### Analysis to assure sterility of apoptotic LNCaP tumor cells.

Aliquots of LNCaP and LNCaP-M1 master cell lines will be tested for sterility and adventitious agents by BioReliance. Aliquots of these master cell lines will be thawed for expansion prior to co-culture with autologous DCs. LNCaP or LNCaP-M1 cells are treated with UV irradiation to induce apoptosis and in some cases, gamma irradiation, prior to co-culture with autologous DCs. Sterility of the apoptotic LNCAP or LNCaP-M1 cells will be assessed following co-culture with autologous DCs.

##### Analysis to assure sterility of DCs.

Dendritic cell cultures will be monitored visually on days 2, 4 and 6 for growth of an organism. This will be monitored by microscopic inspection of the samples as well as observation of the media color-the RPMI 1640 has a pH indicator dye and is sensitive to increased metabolic activity in the culture wells. As a more stringent examination of sterility, microbiologic testing will be performed 48 hours prior to final harvest. These cultures will include dendritic cells and apoptotic LNCaP or LNCaP-M1 cells. 100 microliters of the culturing media will be removed by aseptic technique, pooled and sent for analysis of bacterial, mycoplasma and fungal contamination. In addition, since the culture-based mycoplasma test results will not be available before administration of cellular products, PCR–based or DNA fluorochrome mycoplasma testing will be done.

On Day 8 and / or for release analysis sterility testing will be performed, together with gram stain and LAL test for the presence of endotoxin. (See Vaccine flow diagram, Figure 2).

#### Stability of components

DCs will be monitored for stability during the preparation of the DC-LNCaP product. Cells will be examined daily for morphology and health, and prior to co-culturing with apoptotic LNCaP cells DCs will be monitored by trypan blue exclusion for viability.

LNCaP or LNCaP-M1 cells will be monitored for stability during the preparation of the DC-LNCaP product. Cells grown from frozen stocks will be examined daily for morphology and health, and prior to irradiation cells will be monitored by trypan blue exclusion for viability.

A freezing program has been established (see Appendix IV). As these are adherent cell lines, it is possible to remove non-viable cells during the first cell passage. In this way, the LNCaP and LNCaP-M1 cells are 100% viable at time of induction of apoptosis for DC coculture. Initial recovery of DC/LNCaP and DC/LNCaP-M1 product after thawing is approximately 50-85%. Cell debris is removed by washing in sterile saline. In this manner, it is possible to achieve 50-95% viable cells in the final product. Cells prepared in this fashion have been validated in functional assays and have 95-100% potency as compared to the activity achieved with materials prior to storage. Release criteria will be survival > 30% as determined by comparative cell count and viability of final product > 70% as determined by exclusion of typan blue.

## General method of vaccine preparation

Autologous DCs grown from each prostate cancer patient enrolled in this study will be grown in our GLP tissue-culture facility. In process testing will assure the quality of DCs from each patient, as described in the previous section. Immature DCs are co-cultured with apoptotic LNCaP tumor cells in the presence of maturation stimuli. Lot release tests are performed on the bulk product of all DC/LNCaP cell mixtures prior to freezing one patients’ autologous vaccine for repeat vaccinations. Lot release tests are also performed on each sample prior to subcutaneous injection.

### DC/LNCaP

LNCaP cells are expanded in Aim V media/1% FCS from single vials taken from the master cell bank stock. Each vial contains 1-4 x 10^7^ frozen cells, and thawed cells are grown under GLP conditions to generate a total of 5 x 10^8^ viable LNCaP cells. These LNCaP cells are washed extensively in PBS, irradiated with UV-B light to allow apoptotic cell death to occur (Albert et al., 1998a; Albert et al., 1998c), and placed at 37^o^C for 8-12 hours in 1% autologous plasma. Apoptotic LNCaP cells are then co-cultured with autologous DCs, PGE-2 and TNF-α to allow DC maturation. In some cases, these apoptotic LNCaP cells are treated with gamma irradiation, 5,000 rads, prior to co-culture. Co-cultures are established at a ratio of one apoptotic cell to one DC, determined by the numbers of available DC. DCs are then grown in RPMI media containing 1% autologous plasma for an average of 36 additional hours (average 30-48 hours). The autologous DCs, which have now phagocytosed apoptotic LNCaP cell material (DC/LNCaP), are collected by centrifugation, washed once and counted. 2-10 x 10^6^ cells are resuspended in 0.5 ml 5% DMSO and 25% autologous serum in sterile saline for injection. The remaining DC/LNCaP cells are placed in coded tubes, and stored in liquid nitrogen for use in booster immunizations.

### DC/LNCaP-M1

This vaccine is prepared essentially as for DC/LNCaP cells except that LNCaP cells stably transfected with a cDNA expressing the influenza matrix antigen are used instead of LNCaP cells. LNCaP-M1 cells are expanded in Aim V media/1% FCS from a master stock of 1-4 x 10^7^ frozen cells, to generate a total of 5 x 10^8^ viable LNCaP cells. These LNCaP cells are washed in PBS, irradiated with UV-B light, and placed at 37^o^C for 8-12 hours in 1% autologous plasma. Apoptotic LNCAP-M1 cells are then co-cultured with autologous DCs, PGE-2 and TNF-α to allow DC maturation. In some cases, these apoptotic LNCaP cells are treated with gamma irradiation, 5,000 rads, prior to co-culture. Co-cultures are established at a ratio of one apoptotic cell to one DC, determined by the numbers of available DC. DCs are then grown in RPMI media containing 1% autologous plasma for an average of 36 additional hours (average 30-48 hours). The autologous DCs apoptotic LNCaP-M1 cell mixture (DC/LNCaP-M1), are collected by centrifugation, washed and counted. 2-10 x 10^6^ cells are resuspended in 0.5 ml 5% DMSO and 25% autologous serum in sterile saline for injection. The remaining DC/LNCaP cells are placed in coded tubes, and stored in liquid nitrogen for use in booster immunizations.

## Drug product

**DC/LNCAP:** Autologous dendritic cells which have been exposed to apoptotic LNCAP cells.

**DC/LNCAP-M1:** Autologous dendritic cells which have been exposed to apoptotic LNCAP cells that are stably transfected with a plasmid cDNA expressing the influenza matrix antigen.

**DC/KLH:** Autologous dendritic cells which have been exposed to KLH.

### Components

**Autologous DCs:** Autologous DCs are prepared from leukapheresis of prostate cancer patients entered into the clinical trial.

**LNCaP tumor cells:** The LNCaP prostate tumor cell line was obtained from the ATCC (clone FCG, catalog No. CRL-1740). These cells were maintained throughout growth in our laboratory in defined media, with careful documentation since their arrival in the laboratory. This initially consisted of sterile DMEM (Cell-GRO cat # 10-013-CV) plus 10% USA BSE-free fetal calf serum (FCS) (HyClone cat # SH30071.03, lot # ALA13021). Cells were subsequently weaned to the lowest possible dose of BSE-free FCS, which was 1% in Aim V media, and were subsequently maintained in that state. Frozen stocks were made in Aim V media (Gibco-BRL cat # 12055-091, Lot# 1106795) plus 20% BSE-free FCS and 5% DMSO (Sigma Cat # D2650 Lot# 71K2402).

**Other Reagents:** See Appendix I. Certificates of analysis are available for all reagents that will be used in clinical trial.

### Quantitative composition of Drug Product

DC/LNCaP cells from co-cultured purified components (DC and apoptotic LNCaP cells). Co-cultures will be done at a ratio of one apoptotic LNCAP cell to one autologous DC, determined by the numbers of available LNCaP and DCs. Cells are co-cultured for 30-48 hours and frozen in 5% DMSO, 25% autolgous serum. Aliquots are frozen at a cell concentration of 1-4 x 10^7^ cells/ml.

Aliquots will be thawed, and 2-10 x 10^6^ DCs will be injected.

### Qualitative composition of Drug Product

Quality of the DC/LNCAP product was demonstrated in human cells *ex vivo*. DC/LNCAP cells were prepared as above, except that LNCAP cells were infected with influenza virus to deliver the M1 antigen to these cells prior to induction of apoptotic death. Alternatively, DC/LNCAP-M1 cells were used as immunogen ex vivo. Human cells used to produce DCs were either obtained from normal volunteers or prostate cancer patients. In both instances, DCs of ~ 90% purity were generated, as determined by flow cytometric analysis of DC-specific cell markers. Both normal individuals and prostate cancer patients cells were able to mount antigen-specific CD4^+^ and CD8^+^ T cell responses DC/LNCAP and DC/LNCAP-M1 cells, as assessed by ELISPOT or CTL assay (see (8).iii).

### Manufacturer

Name/address of manufacturer

**DC/LNCAP**

Rockefeller University

1230 York Avenue

New York, New York, 10021

**DC/LNCAP-M1**

Rockefeller University

1230 York Avenue

New York, New York, 10021

**DC/KLH**

Rockefeller University

1230 York Avenue

New York, New York, 10021

### Manufacturing/packaging procedure

##### Drug Formulation and Route of Administration

DC/LNCAP or DC-LNCAP/M1 are suspended in 5% DMSO and 25% autologous serumin sterile saline at a concentration of 1-4 x 10^7^ cells (DCs)/ml. 2-10 x 10^6^ cells are injected (0.5 ml) subcutaneously.

### Acceptable limits and analytical methods to assure identity, strength, quality, purity

#### Strength and Quality

Identity of LNCaP and LNCaP-M1 cells will be confirmed on the Master Cell line, by both Western blot and FACS analysis.

We will analyze each Master Cell line for PSA expression by Western blot analysis. Controls will include frozen stocks of LNCaP cells from the ATCC that are PSA positive.

We will analyze each Master Cell line for PSA expression and HLA A*0201 surface expression by FACS analysis. Controls will include frozen stocks of LNCaP cells from the ATCC that are PSA positive and HLA A*0201.

Quality of DC/LNCaP, DC/LNCaP-M1, and DC/KLH cells

We will analyze each lot of DC/LNCaP, DC/LNCaP-M1, and DC/KLH cells by FACS analysis prior to cell freezing. Cells will be assessed for DC markers, and should have the following profile (see also (7)iii.d.1): After gating on HLA DR, >70% CD83^+^, and <15% CD14^+^.

Aliquots of DC/LNCaP, DC/LNCaP-M1, and DC/KLH cells will be stained for viability with trypan blue and/or propidium iodide. >50% of the cells should exclude trypan blue and/or propidum iodide.

As an additional quality control, we will also intermittently assess the functionality of our frozen-thawed DC/LNCaP, DC/LNCaP-M1 cells. Allo-MLR tests, our primary in vitro assay of DC function, will be performed on DC/LNCaP or DC/LNCaP-M1 cells before and after freezing from cells obtained from our 1^st^, 2^nd^, 4^th^, 8^th^ and 16^th^ patients.

#### Stability

Stability is assessed in frozen/thawed DC/LNCaP preparations.

#### Purity

Purity of the drug product will be assessed by microbiologic testing. Prior to drug release, the following criteria will be met:

- 1. Endotoxin free: less than 5 EU/kg/hr endotoxin, assessed by LAL testing.
  2. Bacteria free: no evidence of bacteria by Gram Stain.
  3. Mycoplasma free: no evidence of mycoplasma by PCR test or fluorochrome test.

In addition, priority of the final drug product will be assessed by microbiology testing for sterility. These results will not be available at the time of drug injection, but will be monitored for each injectate.

## Lot release summary.

### Lot release tests.

Aliquots of master cell lines of LNCaP and LNCaP-M1 cells will be assessed for purity by testing for adventitious agents by BioReliance. In addition, an aliquot of LNCaP and LNCaP-M1 master cell lines will be confirmed as PSA and HLA A*0201 positive by FACS, and PSA and PSMA positive by Western blot.

The final product—DC/LNCaP, DC/LNCaP-M1 or DC/KLH cells—are assessed for sterility and purity prior to injection, as summarized in Table 4 below.

Table 4: Lot Release Summary

| **Components** | **Tests Performed** | **Results** | **Acceptable Limit** |
| --- | --- | --- | --- |
| LNCaP | Microbiologic testing  Endotoxin testing | No growth  <1 EU/kg/hr | no bacterial, mycoplasma or fungal growth  < 5 EU/kg/hr |
| DC | Microbiologic testing  Endotoxin testing | No growth  <1 EU/kg/hr | no bacterial, mycoplasma or fungal growth  < 5 EU/kg/hr |
| DC/LNCaP | Microbiologic testing  Endotoxin testing | No growth  <1 EU/kg/hr | no bacterial, mycoplasma or fungal growth  < 5 EU/kg/hr |
|  | Viability | 75-95% | > 50% thawed cells exclude trypan blue or PI |
|  | Identity | 85-98% DCs | > 70% DCs by FACS |
| DC/LNCaP-M1 | Microbiologic testing  Endotoxin testing | No growth  <1 EU/kg/hr | no bacterial, mycoplasma or fungal growth  < 5 EU/kg/hr |
|  | Viability | 75-95% | > 50% thawed cells exclude trypan blue or PI |
|  | Identity | 85-98% DCs | > 70% DCs by FACS |
| DC/KLH | Microbiologic testing  Endotoxin testing | No growth  <1 EU/kg/hr | no bacterial, mycoplasma or fungal growth  < 5 EU/kg/hr |
|  | Viability | 75-95% | 50% thawed cells exclude trypan blue or PI |
|  | Identity | 85-98% DCs | > 70% DCs by FACS |

## Placebo Identity

### Description of placebo

5% DMSO and 25% autologous plasmain sterile saline will be injected (0.5 ml) subcutaneously.

## Labeling

Copies of all labels to be provided to investigators are shown below.

Patient Name:

Autologous DC/LNCaP vaccine in 5% DMSO and 25% autologous serum in sterile saline

2 - 10 x 10^6^ autologous dendritic cells/0.5 ml. Store at 2-8^o^C

CAUTION: NEW DRUG LIMITED BY FEDERAL LAW TO INVESTIGATIONAL USE; NOT EVALUATED FOR INFECTIOUS SUBSTANCES; FOR AUTOLOGOUS USE ONLY

Rockefeller University Hospital GCRC

Lot: _______Patient ID#_________ Investigator:______________

Patient Name:

Autologous DC/LNCaP-M1 vaccine in 5% DMSO and 25% autologous serumin sterile saline

2 - 10 x 10^6^ autologous dendritic cells/0.5 ml. Store at 2-8^o^C

CAUTION: NEW DRUG LIMITED BY FEDERAL LAW TO INVESTIGATIONAL USE; NOT EVALUATED FOR INFECTIOUS SUBSTANCES; FOR AUTOLOGOUS USE ONLY

Rockefeller University Hospital GCRC

Lot: ______ Patient ID#_______ Investigator: _______________

Patient Name:

Autologous DC/KLH vaccine in 5% DMSO and 25% autologous serum in sterile saline

2 - 10 x 10^6^ autologous dendritic cells/0.5 ml. Store at 2-8^o^C

CAUTION: NEW DRUG LIMITED BY FEDERAL LAW TO INVESTIGATIONAL USE; NOT EVALUATED FOR INFECTIOUS SUBSTANCES; FOR AUTOLOGOUS USE ONLY

Rockefeller University Hospital GCRC

Lot: ______Patient ID# ____________ Investigator: _____________

## Environmental analysis requirement

Exempt § 25.31(e).

# Pharmacology/Toxicology information

## Safety studies

### Animal studies to evaluate drug toxicity

Toxicity of LNCAP cells was assessed in animals to assess whether any LNCaP cells remain viable following the protocol we will use to induce apoptotic death.

We assessed the ability of LNCaP prostate cancer cells to grow in SCID and *nude* mice. Both of these strains are immunoincompetent and thereby provide means of monitoring tumor cell survival. Injection of 5 x 10^7^ apoptotic cells in Matrigel into SCID and *nude* mice produced no evidence of tumor growth after 42 days, while as few as 10 living tumor cells grew into detectable tumors within 21 days following injection (see Figure 4).

Toxicity of DC/LNCaP cells was assessed in animals. Live autologous bone-marrow derived DCs were harvested and differentiated in vitro using GM-CSF for seven days. These cells were then co-cultured with apoptotic LNCaP cells (ratio of 1:1) in the presence of TNF-a and PGE2 for 40 hours. These DC/LNCaP cells were injected into the footpad of 6-8 week old C57BL/6 and BALB/c mice at concentrations of 10^4^, 10^5^, and 10^6^. To attempt to provoke dose-limiting toxicities, mice treated with 10^6^ DC/LNCaP cells were also given an extra vaccination (5 instead of 4 injections) in an accelerated schedule (weekly instead of every other week). Following the last vaccination, mice were sacrificed to perform histological and clinical laboratory (complete blood count and either ANA or dsDNA) examinations. Serum chemistries were also assessed in mice that received 10^4^ and 10^5^ cells. Histologic studies were performed blindly on each group by an independent licensed veterinary pathologist (IDEXX). In each case, results were compared with those of control mice that had been injected with PBS.

The DC/LNCaP cell doses used in these studies correspond, on a live DC cell number/body weight basis, to an excess of approximately 3-fold, 30-fold, and 300-fold, for cell doses of 10^4^, 10^5^, and 10^6^ cells, respectively, relative to the maximum dose (10 x 10^6^ DC/LNCaP cells/injection) to be used in clinical study.

There was no clinical toxicity evident in any of the treated or PBS control animals. Only three reproducible differences in the types of histologic changes seen at necropsy were observed between the PBS injected group and the DC/LNCaP cell injected groups. In mice treated with 10^5^ and 10^6^ DC/LNCaP cells, evidence of inflammation (synovitis or granuloma formation) were evident in the footpads of a significant number of DC/LNCaP injected mice compared to PBS injected mice. In mice given the 10^6^ DC/LNCaP vaccine regimen, inguinal lymphadenitis was consistently seen in the lymph nodes that drained the footpad where vaccine was injected. Finally, in given the 10^6^ DC/LNCaP vaccine regimen, there was evidence of vascular inflammation in the lungs of treated mice. These results are summarized in Table 5, and the formal pathology reports on all mice are included in the Appendix.

Analysis of serum ANA levels revealed that mice treated with 10^6^ DC/LNCaP cells showed an increase in very strongly reactive ANA following injection. No significant differences were seen in the serum chemistry or hematologic profiles of PBS versus DC/LNCaP group other than a mild eosinophilia evident in mice treated with 10^6^ DC/LNCaP cells. The results from all mice are included in the Appendix.

Table 5. Animal Histology Summary

| **DC/LNCaP at 10^4^ and 10^5^ cells injected every 2 weeks for 4 injections in right foot pad assessed at 8 weeks.** | PBS  3 total mice | DC/LNCaP 10^4^  3 total mice | DC/LNCaP 10^5^  3 total mice |
| --- | --- | --- | --- |
| BRAIN | | | |
| No significant changes | 2 | 3 | 3 |
| Meningeal inclusion cyst | 1 | 0 | 0 |
| THYMUS | | | |
| No significant changes | 3 | 3 | 3 |
| HEART | | | |
| No significant changes | 2 | 2 | 2 |
| Myocarditis | 1 | 0 | 0 |
| Valvular melanocytic accumulation | 0 | 1 | 1 |
| LUNG | | | |
| No significant changes | 2 | 2 | 2 |
| Hemorrhage | 1 | 0 | 0 |
| Peribronchiolar lymphoid accumulation | 0 | 1 | 0 |
| Pneumonia | 0 | 0 | 1 |
| LIVER | | | |
| No significant changes | 0 | 0 | 0 |
| Hepatitis | 3 | 3 | 3 |
| Extramedullary hematopoisis | 0 | 1 | 1 |
| KIDNEY | | | |
| No significant changes | 2 | 2 | 3 |
| Proteinuria | 1 | 1 | 0 |
| SPLEEN | | | |
| No significant changes | 0 | 0 | 0 |
| Hemosiderosis | 3 | 3 | 3 |
| SMALL INTESTINE | | | |
| No significant changes | 3 | 3 | 3 |
| LARGE INTESTINE | | | |
| No significant changes | 3 | 3 | 3 |
| RIGHT INGUINAL LYMPH NODE | | | |
| No significant changes | 3 | 3 | 2 |
| Lymphadenitis | 0 | 0 | 1 |
| RIGHT FOOT | | | |
| No significant changes | 2 | 2 | 0 |
| Synovitis | 1 | 1 | 3 |
| LEFT FOOT | | | |
| No significant changes | 3 | 2 | 3 |
| Synovitis | 0 | 1 | 0 |

| **DC/LNCaP at 10^6^ cells injected every 1 week for 5 injections in right foot pad assessed at 6 weeks.** | PBS  3 total mice | DC/LNCaP 10^6^  3 total mice |
| --- | --- | --- |
| BRAIN | | |
| No significant changes | 3 | 3 |
| THYMUS | | |
| No significant changes | 3 | 3 |
| HEART | | |
| No significant changes | 3 | 3 |
| LUNG | | |
| No significant changes | 2 | 0 |
| Hemorrhage | 1 | 0 |
| **Vasculitis** | 0 | 3 |
| LIVER | | |
| Mixed cell infiltration | 3 | 3 |
| KIDNEYS | | |
| No significant changes | 3 | 1 |
| Mononuclear cell infiltration | 0 | 1 |
| Protein Casts | 0 | 1 |
| SPLEEN | | |
| No significant changes | 3 | 3 |
| SMALL INTESTINE | | |
| No significant changes | 3 | 3 |
| LARGE INTESTINE | | |
| No significant changes | 3 | 3 |
| RIGHT INGUINAL LYMPH NODE | | |
| No significant changes | 3 | 0 |
| **Lymphadenitis** | 0 | 3 |
| RIGHT FOOT | | |
| No significant changes | 2 | 0 |
| Inflammation | 1 | 0 |
| Granulomatous inflammation | 0 | 3 |
| LEFT FOOT | | |
| No significant changes | 3 | 3 |

### In vitro studies to evaluate drug toxicity

Toxicity of LNCAP cells was assessed *in vitro* to evaluate whether any LNCaP cells remain viable following the protocol we will use to induce apoptotic death. 1 x 10^6^ apoptotic LNCAP cells that had been treated as they will be in cell immunizations (irradiated with UV-B light, and placed at 37^o^C for 8-12 hours in Aim V and antibiotics). Cells were assessed for cell viability in vitro by measuring uptake of ^3^H-thymidine. No cell growth was evident in apoptotic LNCAP cells (Figure 6).

Figure 6. Apoptotic LNCaP cells are replication incompetent.

### Additional pre-clinical toxicity studies.

General safety tests of LNCaP cells to assess contamination of cells with endotoxin and adventitious agents are discussed in (7.iii) and summarized in (7.vi) as part of proposed lot release-testing.

## Activity studies

### Animal efficacy data.

We have also examined the efficacy of using apoptotic tumor cells *in vivo* using a vaccination strategy modeled after our planned clinical trial. We studied the growth of B16 melanoma in syngeneic mice, a tumor that is classified as non-immunogenic in C57/Bl6 mice. Mice were immunized with three injections of DCs co-cultured with either apoptotic B16 melanoma, or, as a control, a fibrosarcoma cell line (MC57). Following immunization, mice were challenged with 10^5^ B16 melanoma cells. Mice immunized with DCs cross-presenting B16 tumors were completely protected from subsequent tumor growth, while those immunized with DCs cross-presenting MC57 were susceptible (Figure 7). To demonstrate that this immunization resulted from cross-presentation of apoptotic tumor by DCs and not direct immunization by the apoptotic tumor cells themselves, the DCs and tumor cells were mixed in the presence of EDTA and injected. Under these conditions, the apoptotic tumor cells were not phagocytosed by the DC and could not be cross-presented. These DC / apoptotic cell mixtures failed to protect mice against subsequent tumor challenge, confirming the need for apoptotic antigen to be phagocytosed and presented by DCs (Figure 7).

Figure 7. Mouse DCs cross-presenting apoptotic antigen block tumor growth.

### Human efficacy data.

We assessed whether DCs derived from prostate cancer patients are capable of cross-presenting antigen derived from apoptotic LNCaP cells for the activation of antigen-specific T cells. Leukapheresates were obtained from prostate cancer patients under the care of Dr. Scher and in accordance with our IRB protocol RDA 0269.

Prostate patient DCs were co-cultured with apoptotic influenza infected and uninfected LNCaP cells and ELISPOT assays were performed. Both influenza-specific CD4^+^ and CD8^+^ T cells were detected in 7 out of 7 patients. In addition, DCs obtained from HLA A*0201 prostate cancer patients were co-cultured with apoptotic LNCaP-M1 and used to stimulate T cells for 7 days in vitro, allowing us to determine effector CTL function. Cytotoxicity was measured using influenza matrix-peptide pulsed T2 cells as targets, and demonstrated the presence of M1-specific CTLs in 3/4 HLA A*0201 prostate cancer patients. These results demonstrate that apoptotic LNCaP cells are able to effectively cross-present antigen for the activation of CD4^+^ and CD8^+^ T cells. This data is summarized in Table 6 below.

## Tabulation of data suitable for detailed review

Table 6. Tabulation of Human Efficacy Data.

| Assay / Patient ID: | PC04 | PC06^*^ | PC08 | PC09 | PC10^*^ | PC11 | PC12^*^ | Buffy |
| --- | --- | --- | --- | --- | --- | --- | --- | --- |
| **Allo-MLR** (Stimulation Index at 100:1 T:DC ratio) | - | 13 | 16 | 72 | - | - | 36 | 12 |
|  |  |  |  |  |  |  |  |  |
| **ELISPOT Data** (SFC/10^6^ cells, st. deviation) |  |  |  |  |  |  |  |  |
| DC cross-presenting apoptotic: |  |  |  |  |  |  |  |  |
| flu-infected LNCaP | 137 (76) | 310 (115) | 403 (45) | 125 (10) | 433 (34) | 25 (5) | 565 (78) | 193 (35) |
| LNCaP alone | 0 | 17 (2.9) | 5 (0) | 5 (5) | 1.7 (2.9) | 0 (0) | 18 (10) | 3.3 (2.9) |
|  |  |  |  |  |  |  |  |  |
| **CTL Data**  (% specific lysis) |  |  |  |  |  |  |  |  |
| DC cross-presenting apoptotic: |  |  |  |  |  |  |  |  |
| flu-infected LNCaP |  | 38 | - | - | 17 | - | 29 |  |
| LNCaP alone |  | 10 |  |  | 5 |  | 1 |  |

For allo-MLR assay, allogeneic T cells were cocultured with patient mature DCs at ratios of 100:1 (as shown), as well as 10:1, 30:1, 300:1, 1000:1, 2700:1 and 8100:1 (data not shown). Proliferation was measure using ^3^H-thymidine. For ELISPOT assays, LNCaP cells were infected with influenza, irradiated with UVB and cultured to allow apoptotic death. Immature DCs were cocultured for 36 hrs with the apoptotic cells (1 DC:5 apoptotic cells) in the presence of a TNF-α and PGE-2. After 40 hours, the number of antigen-reactive T cells expressing IFN-γ were counted. Uninfected cells served as negative controls. CD8^+^ T cell killing was measured by ^51^Cr from M1 peptide pulsed T2 cells. Importantly, DCs cross presenting LNCaP alone did not stimulate T cell responses over background (EL4 data not shown).

^*^Indicates patient HLA haplotype A*0201.

## Statement that study is conducted in compliance with good lab practice

The studies described in this proposal are to be conducted in compliance with good lab practice.

# Previous human experience with the investigational drug

## Previous investigation/marketing

None

## Previous experience with components

There has been no prior clinical study of safety or efficacy of apoptotic material used to charge autologous DCs.

### Safety and efficacy of DC and tumor cell vaccines.

There have been numerous reports of the use of autologous DCs for immunization, with a remarkably robust safety profile, with an extensive published literature, particularly in the cancer therapy field. More than 100 individuals have been injected with DCs safely with promising results often seen in patients with highly treated, advanced cancer (Bhardwaj, 2001; Fong and Engleman, 2000; Sprinzl et al., 2001). Studies in healthy volunteers demonstrated safety and efficacy in inducing T cell responses to influenza matrix protein peptide, a fragment of the M1 antigen to be used in the current study. In those studies using normal volunteers, patients were injected subcutaneously with 5 x 10^6^ autologous DCs whose purity ranged from 19-95% in one study (Dhodapkar et al., 1999), and 39-74% in a follow-up study (Dhodapkar et al., 2000). No significant toxicity was observed in these patients, and the only toxicity that was seen was characterized by minor local reactions (pain and erythema) at the site of injection. Other transient symptoms, present only on the day of injection, included fatigue (1/8 patients) and low grade fever (1/8 patients). No patient developed rash, lymphadenopathy, or any clinical evidence of autoimmunity (Dhodapkar et al., 1999), even with 6-9 months of follow-up (Bhardwaj, 2001; Dhodapkar et al., 2000). One patient developed transient low-titer (1:40) ANA reactivity at 60 days after immunization with no other serologic or clinical evidence of autoimmunity (Dhodapkar et al., 1999).

Cancer patients treated with DC vaccines include those with melanoma, myeloma, lymphoma, prostate, renal cell, ovarian, breast and colon cancer. There have not been reports of significant acute toxicity in these studies, even with doses of up to 1.2 x 10^8^ mature DCs (Bhardwaj, 2001; Fong and Engleman, 2000).

The published literature suggests the efficacy of DC vaccines. A single injection of antigen bearing, monocyte-derived, mature DCs induce rapid and lasting CD4^+^ and CD8^+^ T-cell immunity in normal human volunteers (Dhodapkar et al., 2000; Dhodapkar et al., 1999). The kinetics and durability of this T cell response were studied and showed that a booster injection of DCs elicited functionally superior T cells that recognized lower doses of antigen compared to those elicited after the initial injection. These DC studies in normal human volunteers were well tolerated with minor local reactions (erythema, no induration) and no systemic toxicity with the exception of transient fatigue (~10%) and low grade fever (10%) on the day of injection. The booster DC injections were similarly well tolerated and again restricted to local reactivity. Some studies have shown regression of cutaneous metastases mediated by CD8^+^ T lymphocytes.

Some studies have reported the safe use of tumor cell pulsed DCs. The mode of death of tumor cells (necrotic death) in these instances differs from the active induction of apoptotic death and the related processing of antigen for delivery to DCs in the current proposed study. Nonetheless, studies in which dead (necrotic) tumor cells were pulsed onto DCs have reported no significant acute toxicity (Geiger et al., 2000; Kugler et al., 2000; Yu et al., 2001). These studies have reported occasional partial and complete tumor remissions in some of patients.

### Prostate cancer: safety and efficacy of DC and tumor vaccines

Prostate cancer patients have previously been treated with peptide-pulsed DCs (Lodge et al., 2000; Murphy et al., 1996; Salgaller et al., 1998; Tjoa et al., 1998) or with DCs pulsed with recombinant prostatic acid phosphatase (PAP) (Fong et al., 2001), and none of these studies were associated with significant toxicity. One study has suggested an increased efficacy of intradermal injection over intravenous injection of DC vaccine; only in the former population of prostate cancer patients was IFNγ production induced (Fong et al., 2001). These data are consistent with a number of studies supporting our use of subcutaneous injection of vaccine as both safe and the most efficacious approach to immunization (Bhardwaj, 2001).

Irradiated autologous prostate tumor cells transfected with a gene expressing GM-CSF has been used in clinical trial in prostate cancer patients, with no reported adverse reactions and with evidence of induction of prostate-tumor immune responses in vitro (Simons et al., 1999).

### Detailed information on previous trial relevant to assessment of effectiveness.

None.

### Detailed Bibliography

Albert, M. L., Darnell, J. C., Bender, A., Francisco, L. M., Bhardwaj, N., and Darnell, R. B. (1998a). Tumor-specific killer cells in paraneoplastic cerebellar degeneration. Nat Med 4 , 1321-1324.

Albert, M. L., Pearce, S. F., Francisco, L. M., Sauter, B., Roy, P., Silverstein, R. L., and Bhardwaj, N. (1998b). Immature dendritic cells phagocytose apoptotic cells via alphavbeta5 and CD36, and cross-present antigens to cytotoxic T lymphocytes. J Exp Med 188 , 1359-1368.

Albert, M. L., Sauter, B., and Bhardwaj, N. (1998c). Dendritic cells acquire antigen from apoptotic cells and induce class I- restricted CTLs. Nature 392 , 86-89.

Bhardwaj, N. (2001). Processing and presentation of antigens by dendritic cells: implications for vaccines. Trends Mol Med 7 , 388-394.

Bubley, G. J., Carducci, M., Dahut, W., Dawson, N., Daliani, D., Eisenberger, M., Figg, W. D., Freidlin, B., Halabi, S., Hudes, G., et al. (1999). Eligibility and response guidelines for phase II clinical trials in androgen-independent prostate cancer: recommendations from the Prostate- Specific Antigen Working Group. J Clin Oncol 17 , 3461-3467.

Curtis, J. E., Hersh, E. M., Butler, W. T., and al., e. (1971). Antigen dose in the human immune response. J Lab Clin Med 78 , 61-69.

Darnell, R. B. (1999). The importance of defining the paraneoplastic neurologic disorders. N Engl J Med 340 , 1831-1833.

Darnell, R. B., and Posner, J. B. (2003). Paraneoplastic syndromes involving the nervous system. N Engl J Med 349 , 1543-1554.

Dhodapkar, M. V., and Bhardwaj, N. (2000). Active immunization of humans with dendritic cells. J Clin Immunol 20 , 167-174.

Dhodapkar, M. V., Krasovsky, J., Steinman, R. M., and Bhardwaj, N. (2000). Mature dendritic cells boost functionally superior CD8(+) T-cell in humans without foreign helper epitopes [see comments]. J Clin Invest 105 , R9-R14.

Dhodapkar, M. V., Steinman, R. M., Sapp, M., Desai, H., Fossella, C., Krasovsky, J., Donahoe, S. M., Dunbar, P. R., Cerundolo, V., Nixon, D. F., and Bhardwaj, N. (1999). Rapid generation of broad T-cell immunity in humans after a single injection of mature dendritic cells. J Clin Invest 104 , 173-180.

Fazzari, M., Heller, G., and Scher, H. I. (2000). The phase II/III transition. Toward the proof of efficacy in cancer clinical trials. Control Clin Trials 21 , 360-368.

Fong, L., Brockstedt, D., Benike, C., Wu, L., and Engleman, E. G. (2001). Dendritic cells injected via different routes induce immunity in cancer patients. J Immunol 166 , 4254-4259.

Fong, L., and Engleman, E. G. (2000). Dendritic cells in cancer immunotherapy. Annu Rev Immunol 18 , 245-273.

Geiger, J., Hutchinson, R., Hohenkirk, L., McKenna, E., Chang, A., and Mule, J. (2000). Treatment of solid tumours in children with tumour-lysate-pulsed dendritic cells. Lancet 356 , 1163-1165.

Geiger, J. D., Hutchinson, R. J., Hohenkirk, L. F., McKenna, E. A., Yanik, G. A., Levine, J. E., Chang, A. E., Braun, T. M., and Mule, J. J. (2001). Vaccination of pediatric solid tumor patients with tumor lysate-pulsed dendritic cells can expand specific T cells and mediate tumor regression. Cancer Res 61 , 8513-8519.

Gotch, F., Rothbard, J., Howland, K., Townsend, A., and McMichael, A. (1987). Cytotoxic T lymphocytes recognize a fragment of influenza virus matrix protein in association with HLA-A2. Nature 326 , 881-882.

Inaba, K., Turley, S., Yamaide, F., Iyoda, T., Mahnke, K., Inaba, M., Pack, M., Subklewe, M., Sauter, B., Sheff, D., et al. (1998). Efficient presentation of phagocytosed cellular fragments on the major histocompatibility complex Class II products of dendritic cells. J Exp Med 188 , 2163-2173.

Klippel, K. F., Paulini, G., and Hutschenreiter, G. (1977). The effect of keyhole limpet hemocyanin (KLH) on the rat bladder. Cancer Immunol Immunother 3 , 65-67.

Kugler, A., Stuhler, G., Walden, P., Zoller, G., Zobywalski, A., Brossart, P., Trefzer, U., Ullrich, S., Muller, C. A., Becker, V., et al. (2000). Regression of human metastatic renal cell carcinoma after vaccination with tumor cell-dendritic cell hybrids. Nat Med 6 , 332-336.

Landis, S. H., Murray, T., Bolden, S., and Wingo, P. A. (1999). Cancer statistics, 1999 [see comments]. CA Cancer J Clin 49 , 8-31, 31.

Li, M., Davey, G. M., Sutherland, R. M., Kurts, C., Lew, A. M., Hirst, C., Carbone, F. R., and Heath, W. R. (2001). Cell-associated ovalbumin is cross-presented much more efficiently than soluble ovalbumin in vivo. J Immunol 166 , 6099-6103.

Lodge, P. A., Jones, L. A., Bader, R. A., Murphy, G. P., and Salgaller, M. L. (2000). Dendritic cell-based immunotherapy of prostate cancer: immune monitoring of a phase II clinical trial. Cancer Res 60 , 829-833.

Markiewicz, M. A., and Gajewski, T. F. (1999). The immune system as anti-tumor sentinel: molecular requirements for an anti-tumor immune response. Crit Rev Oncog 10 , 247-260.

Murphy, G., Tjoa, B., Ragde, H., Kenny, G., and Boynton, A. (1996). Phase I clinical trial: T-cell therapy for prostate cancer using autologous dendritic cells pulsed with HLA-A0201-specific peptides from prostate-specific membrane antigen. Prostate 29 , 371-380.

Okada, H., Pollack, I. F., Lieberman, F., Lunsford, L. D., Kondziolka, D., Schiff, D., Attanucci, J., Edington, H., Chambers, W., Kalinski, P., et al. (2001). Gene therapy of malignant gliomas: a pilot study of vaccination with irradiated autologous glioma and dendritic cells admixed with IL-4 transduced fibroblasts to elicit an immune response. Hum Gene Ther 12 , 575-595.

Olsson, C. A., Rao, C. N., Menzoian, J. O., and al., e. (1972). Immunologic unreactivity in bladder cancer patients. J Urol 107 , 607-609.

Pound, C. R., Partin, A. W., Eisenberger, M. A., Chan, D. W., Pearson, J. D., and Walsh, P. C. (1999). Natural history of progression after PSA elevation following radical prostatectomy [see comments]. Jama 281 , 1591-1597.

Rieser, C., Bock, G., Klocker, H., Bartsch, G., and Thurnher, M. (1997). Prostaglandin E2 and tumor necrosis factor alpha cooperate to activate human dendritic cells: synergistic activation of interleukin 12 production. J Exp Med 186 , 1603-1608.

Salgaller, M. L., Tjoa, B. A., Lodge, P. A., Ragde, H., Kenny, G., Boynton, A., and Murphy, G. P. (1998). Dendritic cell-based immunotherapy of prostate cancer. Crit Rev Immunol 18 , 109-119.

Sallusto, F., and Lanzavecchia, A. (1994). Efficient presentation of soluble antigen by cultured human dendritic cells is maintained by granulocyte/macrophage colony-stimulating factor plus interleukin 4 and downregulated by tumor necrosis factor alpha. J Exp Med 179 , 1109-1118.

Scardino, P. T., Weaver, R., and Hudson, M. A. (1992). Early detection of prostate cancer. Hum Pathol 23 , 211-222.

Scher, H. I. (1999). Management of prostate cancer after prostatectomy: treating the patient, not the PSA [editorial; comment]. JAMA 281 , 1642-1645.

Scher, H. I. (2003). Prostate carcinoma: defining therapeutic objectives and improving overall outcomes. Cancer 97 , 758-771.

Simons, J. W., Mikhak, B., Chang, J. F., DeMarzo, A. M., Carducci, M. A., Lim, M., Weber, C. E., Baccala, A. A., Goemann, M. A., Clift, S. M., et al. (1999). Induction of immunity to prostate cancer antigens: results of a clinical trial of vaccination with irradiated autologous prostate tumor cells engineered to secrete granulocyte-macrophage colony-stimulating factor using ex vivo gene transfer. Cancer Res 59 , 5160-5168.

Slovin, S. F., and Scher, H. I. (1999). Rising PSAs after primary therapy: active or passive intervention. Semin Urol Oncol 17 , 164-173.

Sprinzl, G. M., Kacani, L., Schrott-Fischer, A., Romani, N., and Thumfart, W. F. (2001). Dendritic cell vaccines for cancer therapy. Cancer Treat Rev 27 , 247-255.

Susser, E., Desvarieux, M., and Wittkowski, K. M. (1998). Reporting sexual risk behavior for HIV: a practical risk index and a method for improving risk indices. Am J Public Health 88 , 671-674.

Therasse, P., Arbuck, S. G., Eisenhauer, E. A., Wanders, J., Kaplan, R. S., Rubinstein, L., Verweij, J., Van Glabbeke, M., van Oosterom, A. T., Christian, M. C., and Gwyther, S. G. (2000). New guidelines to evaluate the response to treatment in solid tumors. European Organization for Research and Treatment of Cancer, National Cancer Institute of the United States, National Cancer Institute of Canada. J Natl Cancer Inst 92 , 205-216.

Thurner, B., Roder, C., Dieckmann, D., Heuer, M., Kruse, M., Glaser, A., Keikavoussi, P., Kampgen, E., Bender, A., and Schuler, G. (1999). Generation of large numbers of fully mature and stable dendritic cells from leukapheresis products for clinical application. J Immunol Methods 223 , 1-15.

Tjoa, B. A., Simmons, S. J., Bowes, V. A., Ragde, H., Rogers, M., Elgamal, A., Kenny, G. M., Cobb, O. E., Ireton, R. C., Troychak, M. J., et al. (1998). Evaluation of phase I/II clinical trials in prostate cancer with dendritic cells and PSMA peptides. Prostate 36 , 39-44.

Yu, J. S., Wheeler, C. J., Zeltzer, P. M., Ying, H., Finger, D. N., Lee, P. K., Yong, W. H., Incardona, F., Thompson, R. C., Riedinger, M. S., et al. (2001). Vaccination of malignant glioma patients with peptide-pulsed dendritic cells elicits systemic cytotoxicity and intracranial T-cell infiltration. Cancer Res 61 , 842-847.

# Appendices

# Drug formulations

Certificates of Analysis of all drugs used are maintained.

**KLH**

DESCRIPTION: Keyhole limpet hemocyanin (KLH) is a glycoprotein used to access immune responses in clinical trials. This product will be purchased from Intracel Corporation.

HOW SUPPLIED: KLH is supplied in a 1 ml vial, at a concentration of 20 mg/ml in water.

STORAGE: The vialed product is stored at 2-8^o^ C. DO NOT FREEZE.

STABILITY: The vialed product is stable for at least 15 months when stored at 2-8^o^ C.

ORDERING: Additional KLH (same lot #) can be obtained from biosynCorporation.

**INTERLEUKIN 4 (IL-4)**

DESCRIPTION: IL-4 is synthesized by R and D Systems at ex vivo grade.

HOW SUPPLIED: The product is supplied as a lyophilized powder.

STORAGE: -20 to –70^o^C

STABILITY: 18 months

ORDERING: Additional IL-4 (same lot #) can be obtained from R and D Systems.

**GRANULOCYTE/MONOCYTE COLONY STIMULATING FACTOR (GM-CSF)**

DESCRIPTION: GM-CSF is commercially synthesized by Immunex and is used to increase the number and function of white blood cells. It is available in GMP grade, at a concentration of 500 μg/ml/vial.

HOW SUPPLIED: In water.

STORAGE: Stored at at 2-4^0^C

STABILITY: 18 months

ORDERING: Additional GM-CSF (same lot #) can be obtained from Immunex

**rhTNF-α**

DESCRIPTION: Recombinant human TNF-alpha is used to in the ex vivo maturation of dendritic cells. It is available in ex vivo grade from R and D Systems. .

HOW SUPPLIED: 126 μg vials.

STORAGE: Reconstituted at 100μg/ml, 20^o^C

STABILITY: 18 months

ORDERING: Available from R and D Systems.

**PGE-2**

DESCRIPTION: PGE-2 is used to in the ex vivo maturation of dendritic cells. It is available in GMP grade.

HOW SUPPLIED: 1 mg vial

STORAGE: -20 to –70^o^C, 200μM aliquots.

STABILITY: 18 months

ORDERING: Pharmacia,Belgium.

**ALTERNATIVE PGE2**

DESCRIPTION: Research grde.

HOW SUPPLIED: 10 mg vial.

STORAGE: -20 ^o^C

ORDERING: Sigma-Aldrich

**TETANUS TOXOID, USP.**

DESCRIPTION: A protein antigen supplied in clinical grade by Aventis.

HOW SUPPLIED: Lyophilized

STORAGE: 2-8^o^C.

STABILITY: Stored in normal saline. Final 0.2 units/dose (0.1ml).

ORDERING: Additional tetanus toxoid is available from Aventis.

**DMSO (CRYOSERV)**

DESCRIPTION: A membrane stabilizing agent.

GRADE: Sterile, non-pyrogenic, not suitable for injection, not less than 99.0% dimethyl sulfoxide USP

HOW SUPPLIED: 10 ml vials

STORAGE: Room temperature

ORDERING: Edwards Lifesciences

**AIM V**

DESCRIPTION; Serum free media by Invitrogen for LNCaP and LNCaP-M1 cells.

GRADE: therapeutic grade

HOW SUPPLIED: 1 liter bottles

STORAGE: 2-8 ^o^C

STABILITY: 18 months

ORDERING: Additional bottles can be ordered from Invitrogen

**DULBECCO’S MODIFICATION OF EAGLE’S MEDIUM, 1X**

DESCRIPTION; Added to media for LNCaP and LNCaP-M1 cells.

GRADE: in vitro diagnostic grade

HOW SUPPLIED: 100 ml bottles

STORAGE: 2-8 ^o^C, protect from light

STABILITY: 18 months

ORDERING: Cellgro

**FETAL BOVINE SERUM**

DESCRIPTION; Added to media for LNCaP and LNCaP-M1 cells.

GRADE: characterized, USDA inspected, for in vitro diagnostic/cell culture use

HOW SUPPLIED: 500 ml bottles

STORAGE: -20 ^o^C, protect from light

ORDERING: Hyclone

**G418**

DESCRIPTION; Added to media for LNCaP-M1 cells.

GRADE: research grade

HOW SUPPLIED: 50 mg/ml, 20 ml bottles

STORAGE: 2-8 ^o^C, protect from light

ORDERING: GIBCO Invitrogen

**GENTAMICIN**

DESCRIPTION; Added to media for LNCaP and LNCaP-M1 cells and dendritic cells.

GRADE: USP

HOW SUPPLIED: 40 mg/ml, 2 ml vials

STORAGE: room temperature

ORDERING: Abbott Laboratories

**HEPES BUFFER SOLUTION, 1M**

DESCRIPTION; Added to media for LNCaP and LNCaP-M1 cells and dendritic cells.

GRADE: in vitro diagnostic grade

HOW SUPPLIED: 100 ml bottles

STORAGE: room temperature

STABILITY: 24 months

ORDERING: Cellgro

**HUMAN ALBUMIN (ALBUTEIN) 25%**

DESCRIPTION; For reconstituting cytokines and for freezing down dendritic cells

GRADE: USP, suitable for IV administration

HOW SUPPLIED: 50 ml vial (25%)

STORAGE: not to exceed 30 ^o^C, do not freeze

ORDERING: Alpha Therapeutics

**L-GLUTAMINE, 200 mM**

DESCRIPTION; Added to media for LNCaP and LNCaP-M1 cells.

GRADE: for in vitro diagnostic use

HOW SUPPLIED: 100 ml bottles

STORAGE: -5 to -20 ^o^C

STABILITY: 24 months

ORDERING: Cellgro

**LYMPHOCYTE SEPARATION MEDIUM**

DESCRIPTION; Density gradient for cell separation

GRADE: for laboratory use

HOW SUPPLIED: 100 ml bottles

STORAGE: 15-30^o^C

ORDERING: Cellgro

**MEM NON-ESSENTIAL AMINO ACIDS SOLUTION, 10mM**

DESCRIPTION; Added to media for LNCaP and LNCaP-M1 cells.

GRADE: for in vitro diagnostic use

HOW SUPPLIED: 100 ml bottles

STORAGE: 2-8^o^C

ORDERING: Cellgro or GIBCO

**2-MERCAPTOETHANOL**

DESCRIPTION; Added to media for LNCaP and LNCaP-M1 cells.

GRADE: for research use

HOW SUPPLIED: 200 ml bottles

STORAGE: 2-8^o^C

ORDERING: GIBCO

**MEM SODIUM PYRUVATE SOLUTION, 100mM**

DESCRIPTION; Added to media for LNCaP and LNCaP-M1 cells.

GRADE: for in vitro diagnostic use

HOW SUPPLIED: 100 ml bottles

STORAGE: 2-8^o^C

ORDERING: Cellgro or GIBCO

**DULBECCO’S PHOSPHATE BUFFERED SALTS, 1X WITHOUT CALCIUM AND MAGNESIUM**

DESCRIPTION; Used for washing LNCaP and LNCaP-M1 cells.

GRADE: for in vitro diagnostic use

HOW SUPPLIED: 500 ml bottles

STORAGE: room temperature

STABILITY: 36 months

ORDERING: Cellgro

**RPMI-1640**

DESCRIPTION: Added to media for dendritic cells.

GRADE: for in vitro diagnostic use

HOW SUPPLIED: 500 ml bottles

STORAGE: 2-8^o^C

ORDERING: Cellgro

**0.9% SODIUM CHLORIDE INJECTABLE**

DESCRIPTION: For resuspending DC/LNCaP and DC/LNCaP-M1 for injection

GRADE: USP, injectable

HOW SUPPLIED: 20 ml bottles

STORAGE: room temperature

ORDERING: Abbott Laboratories

**TRYPSIN EDTA, 1X**

DESCRIPTION; For dissociation of LNCaP and LNCaP-M1 cells.

GRADE: for in vitro diagnostic use

HOW SUPPLIED: 100 ml bottles

STORAGE: -5 to -20^o^C

STABILITY: 12 months

ORDERING: Cellgro

**LAL REAGENT**

DESCRIPTION; Endotoxin testing as release criteria of vaccine

GRADE: for in vitro diagnostic use

HOW SUPPLIED: single test vials

STORAGE: 2-8^o^C

ORDERING: Associates of Cape Cod

# VENORGeM

DESCRIPTION: Mycoplasma Detection Kit

GRADE: invitro diagnostic use

ORDERING: Sigma Product Code: MP0025

**MYOCOALERT**

DESCRIPTION: Mycoplasma Detection Kit

GRADE: in vitro diagnostic use

ORDERING: Cambrex

# 
